# Design, synthesis and evaluation of novel tetrahydropyridothienopyrimidin-ureas as cytotoxic and anti-angiogenic agents

**DOI:** 10.1038/s41598-022-13515-4

**Published:** 2022-06-11

**Authors:** Rasoul Motahari, Mohammad Amin Boshagh, Setareh Moghimi, Fariba Peytam, Zaman Hasanvand, Tayebeh Oghabi Bakhshaiesh, Roham Foroumadi, Hamidreza Bijanzadeh, Loghman Firoozpour, Ali Khalaj, Rezvan Esmaeili, Alireza Foroumadi

**Affiliations:** 1 Department of Medicinal Chemistry, Faculty of Pharmacy, Tehran University of Medical Sciences, Tehran, Iran; 2 Genetics Department, Breast Cancer Research Center, Motamed Cancer Institute, ACECR, Tehran, Iran; 3 Drug Design and Development Research Center, The Institute of Pharmaceutical Sciences (TIPS), Tehran University of Medical Sciences, Tehran, Iran; 4 Department of Pharmacology, School of Medicine, Tehran University of Medical Sciences, Tehran, Iran; 5 Department of Environmental Sciences, Faculty of Natural Resources and Marine Sciences, Tarbiat Modares University, Tehran, Iran

**Keywords:** Cancer, Drug discovery

## Abstract

The novel derivatives of tetrahydropyridothienopyrimidine-based compounds have been designed and efficiently synthesized with good yields through seven steps reaction. The anticancer activity of compounds **11a-y** has been evaluated against MCF-7, PC-3, HEPG-2, SW-480, and HUVEC cell lines by MTT assay. The target compounds showed IC_50_ values between 2.81–29.6 μg/mL and were compared with sorafenib as a reference drug. Among them, compound **11n** showed high cytotoxic activity against four out of five examined cell lines and was 14 times more selective against MRC5. The flow cytometric analysis confirmed the induction of apoptotic cell death by this compound against HUVEC and MCF-7 cells. In addition, **11n** caused sub-G1 phase arrest in the cell cycle arrest. Besides, this compound induced anti-angiogenesis in CAM assay and increased the level of caspase-3 by 5.2 fold. The western-blot analysis of the most active compound, **11n**, revealed the inhibition of VEGFR-2 phosphorylation. Molecular docking study also showed the important interactions for compound **11n**.

## Introduction

As a normal process controlled by biomolecules, angiogenesis is crucial in activities like embryo development, wound healing, etc. Besides, this process lead to the formation and maintenance of blood vessel structures which is important for the progression of different types of disease like cancer^1^. In this regard, antiangiogenic anticancer therapy has been considered as a treatment approach when Folkman and colleagues revealed that the growth of tumors beyond a limited volume needs neovascularization^2–4^. These blood vessels provide oxygen and nutrients and eliminate waste materials generated from tumor metabolism. In addition, they also help cell dissemination which subsequently results in metastasis^5–7^. Considering as the common regulator of tumor angiogenesis, including endothelial cell activation, proliferation and migration, vascular endothelial growth factor (VEGF) pathway has been validated as a therapeutic approach for patients with several tumor types^8^. The regulation of vascular permeability, the induction of gene expression, and the promotion of cell migration, proliferation, and survival are among the imperative responsibilities of VEGF^9,10^. The VEGF family (vascular endothelial growth factor family-A, B, C, and D) account imperative for angiogenesis with high affinity to tyrosine kinase receptors, namely, VEGFR-1, VEGFR-2, and VEGFR-3. VEGF and its receptors are remarkably overexpressed during cancer progression and development. In light of the importance of VEGFR-2 as a target for anti-angiogenesis therapy, several VEGFR-2 small-molecule inhibitors have been introduced to the market^11,12^. Sorafenib, Sunitinib, Pazopanib, Lenvatinib, Vandetanib, and Cabozanitinib are among the approved drugs^13^. There are evidences that antiangiogenic therapy using VEGF inhibitors resulted in clinical benefits among cancer patients. However, several critical challenges have to be overcome before the field may advance in a notable way. In this work, we inspired from the structure of Sorafenib (a dual Raf kinase/vascular endothelial growth factor receptor inhibitor as a multikinase inhibitor which has the urea moiety with applications in thyroid cancer, hepatocellular carcinoma and metastatic renal cancer.

**Figure 1:**
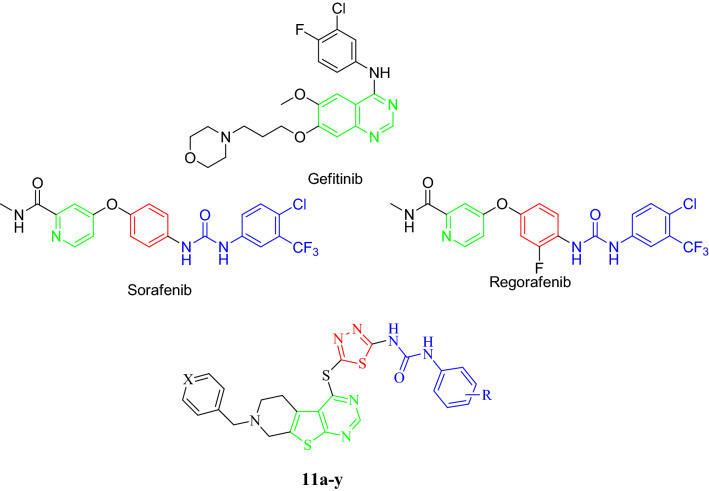
Structure-based design of the target compounds by structural modification of VEGFR-2 inhibitors.

Regarding the high costs of developing anticancer drugs, a hybrid pharmacophore is a useful approach to improve the efficacy of approved drugs and reduce their side effects simultaneously. As a class of nitrogen containing heterocyclic compounds, thienopyrimidine derivatives have been known as tyrosine kinase inhibitors along with remarkable biological activities such as antimicrobial, prolyl hydroxylase antagonist, antimalarial, and anti-inflammatory. Besides, uracil, thymine, and cytosine nucleobases have shown similar structures to pyrimidine^,15–20^. In recent years, urea-based derivatives have attracted attentions in drug design and development, since this functionality caused improved drug potency and selectivity^21^. Furthermore, three out of thirty kinase inhibitors that are approved by FDA, Sorafenib, Regorafenib, and Lenvatinib, have been shown to contain the urea moiety. The presence of a hydrogen bond acceptor meaning urea or amide along with heterocyclic aromatic core are among the most important structural features of these inhibitors. According to our previous findings on thienopyrimidines^22,23^, scaffold hopping and redesign approaches were applied to investigate new series of novel thienopyrimidine derivatives considering the structure of sorafenib and synthesized compounds by Yang in which the hydrophobic tail (occupied the allosteric binding region) is substituted phenyl ring^24^. The bioisoesteric replacement of phenyl in quinazoline core by thiophene ring resulted in our designed compounds (Fig. 1). This study is planned to design and synthesize tetrahydropyridothienopyrimidine derivatives along with the investigation of MTT, apoptosis inducing, cell-cycle arrest, anti-angiogenesis, western-blot and molecular docking studies.

## Results

### Chemistry

**Scheme 1:**
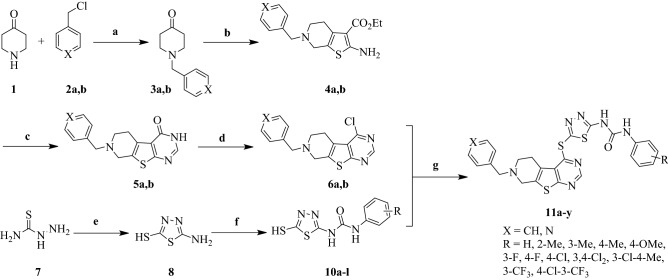
Reagents and conditions: (**a**) K_2_CO_3_, DMF, 80 °C, 5 h; (**b**) sulfur, ethylcyanoacetate, morpholine, EtOH, Reflux, 3 h; (**c**) Formamidine acetate, DMF, 100 °C, 16 h; (**d**) POCl_3_, DBU, 50 °C, 7 h; (**e**) CS_2_, Na_2_CO_3_, EtOH, Reflux, 8 h; (**f**) phenylisocyanate derivatives, DIPEA, DCM (dry), r.t., 5 h; (**g**) DBU, EtOH, 50 °C, 5 h.

The synthetic approach toward targeted tetrahydropyrido[4',3':4,5]thieno[2,3-*d*]pyrimidine-1,3,4-thiadiazole-aryl urea derivatives **11a-y** is outlined in Scheme 1. Initially, 7-substituted-4-chloro-5,6,7,8-tetrahydropyrido[4',3':4,5]thieno[2,3-*d*]pyrimidines **6a,b** was synthesized through a multi-step synthetic routes. This protocol involved the nucleophilic reaction between piperidin-4-one **1** and 4-(chloromethyl)pyridine or benzyl chloride **2a,b** using potassium carbonate (K_2_CO_3_) in dimethylformamide (DMF) at 80 °C to obtain compounds **3a,b**. Afterward, this adduct went through Gewald reaction using ethyl cyanoacetate and sulfur in the presence of morpholine under the reflux condition in EtOH (compounds **4a,b**). The procedure was done according to the reference with modifications^25^. The cyclization reaction using formamidine acetate in DMF carried out at 100 °C to form 7-substituted-5,6,7,8-tetrahydropyrido[4',3':4,5]thieno[2,3-*d*]pyrimidine skeleton **5a,b**. Chlorination of carbonyl group using phosphoryl chloride in the presence of DBU at 50 °C yielded compounds **6a**,**b**.

On the other hand, 1-(5-mercapto-1,3,4-thiadiazol-2-yl)-3-aryl ureas **10a-l** were synthesized through the condensation of thiosemicarbazide **7** and carbon disulfide under the reflux condition in EtOH (compound **8**), followed by addition to various substituted phenyl isocyanates **9a-l** in the presence of *N,N*-diisopropylethylamine (DIPEA) in dry dichloromethane (DCM) at ambient temperature. Finally, chlorine atom in compounds **6a,b** underwent nucleophilic aromatic substitution with synthesized compounds **10a-l** in the presence of 1,8-diazabicyclo(5,4,0)undec-7-ene (DBU) in EtOH at 50 °C to afford our desirable products **11a-y**. The structures of isolated products were deduced on the basis of their IR, ^1^H and ^13^C NMR, as well as mass spectroscopy. Partial assignments of these resonances are given in the experimental part.

### Antiproliferative activity

Two series of new tetrahydropyridothienopyrimidines were evaluated for their cytotoxicity against a panel of cell lines, including MCF-7 (breast cancer), SW480 (colon cancer), PC-3 (prostate cancer), HEPG-2 (liver cancer) and HUVEC (human umbilical vein endothelial cell) using 3-(4,5-dimethyl- thiazol-2-yl)-2,5-diphenyltetrazolium bromide (MTT) assay^26^. Sorafenib was used as a positive control. The results are depicted in Table 1. The cytotoxicity is expressed as the concentration that inhibits 50% of cell viability (IC_50_). As illustrated in Table 1, in general, the target compounds exhibited good cytotoxic activity. The preliminary SAR is evaluated on the basis of cytotoxicity results of phenyl derivatives (**11a–k)** and pyridine derivatives **(11l–11y)**.The mesomeric effect didn’t improve the cytotoxicity of compound **11d** in the first series. Those derivatives with methyl and fluorine substituents at *meta* position are more potent against HUVEC than *para*-substituted counterparts (**11b** *vs* **11c**/**11e** *vs* **11f**/**11q** *vs* **11r**). The presence of fluorine at *para* position of the phenyl ring significantly decreases the activity and the change of this atom with chlorine didn’t improve the activity. The extra chlorine atom at *meta* position generated compound **11h** which shows a significant inhibitory effect on HUVEC (IC_50_ = 7.44 µM). Compounds **11b** and **11h** are more active against MCF-7 in comparison to other derivatives. In the first series, this electronegative atom at *para* position, **11f**, showed weaker activity against SW480 and HUVEC compared to chlorine-substituted analogue, **11g**.

**Table 1:** The in vitro antiproliferative effects of the synthesized compounds.

Compound	R	X	MCF-7	PC-3	SW480	HEPG-2	HUVEC
**11a**	H	C	23.34 ± 0.18	34.44 ± 1.64	24.52 ± 0.72	7.34 ± 0.01	19.06 ± 0.12
**11b**	3-Me	C	8.22 ± 0.09	46.77 ± 1.49	67.65 ± 2.53	48.10 ± 0.22	18.89 ± 0.09
**11c**	4-Me	C	42.87 ± 0.32	32.21 ± 0.30	70.15 ± 2.71	24.57 ± 0.39	26.12 ± 0.15
**11d**	4-OMe	C	51.92 ± 0.47	43.96 ± 1.52	45.07 ± 1.70	43.92 ± 0.16	44.15 ± 0.22
**11e**	3-F	C	13.02 ± 0.18	42.83 ± 1.82	65.60 ± 1.74	9.84 ± 0.06	27.48 ± 0.07
**11f**	4-F	C	45.04 ± 0.41	41.43 ± 0.46	61.29 ± 2.02	30.69 ± 0.44	47.98 ± 0.27
**11g**	4-Cl	C	62.38 ± 2.94	41.60 ± 1.10	56.53 ± 1.28	46.96 ± 0.35	27.29 ± 0.19
**11h**	3,4-*Di*Cl	C	8.52 ± 0.02	70.15 ± 1.92	64.16 ± 2.2	53.71 ± 0.5	7.44 ± 0.06
**11i**	3-Cl-4-Me	C	50.82 ± 0.54	41.76 ± 1.22	69.58 ± 1.88	3.33 ± 0.07	24.63 ± 0.22
**11j**	3-CF_3_	C	68.94 ± 0.63	73.77 ± 2.92	46.79 ± 1.13	62.99 ± 0.64	67.10 ± 1.85
**11k**	3-CF_3-_4-Cl	C	24.66 ± 0.24	23.15 ± 0.21	63.47 ± 1.37	28.46 ± 0.25	37.56 ± 0.31
**11l**	2-Me	N	2.80 ± 0.03	6.11 ± 0.08	14.98 ± 0.38	9.94 ± 0.07	2.53 ± 0.05
**11m**	3-Me	N	3.35 ± 0.01	11.79 ± 0.11	61.23 ± 0.44	27.76 ± 0.13	1.15 ± 0.03
**11n**	4-Me	N	2.67 ± 0.21	11.35 ± 0.09	6.84 ± 0.05	7.20 ± 0.03	2.09 ± 0.08
**11o**	3-OMe	N	14.63 ± 0.07	33.65 ± 1.41	65.80 ± 0.28	69.32 ± 0.52	20.58 ± 0.50
**11p**	4-OMe	N	3.72 ± 0.55	43.49 ± 1.62	29.40 ± 1.01	67.40 ± 0.33	2.86 ± 0.05
**11q**	3-F	N	9.17 ± 0.04	4.50 ± 0.03	21.79 ± 0.72	74.26 ± 2.12	10.13 ± 0.05
**11r**	4-F	N	5.69 ± 0.03	18.98 ± 0.11	66.34 ± 1.24	67.49 ± 1.38	48.24 ± 0.62
**11s**	2-Cl	N	4.69 ± 0.09	8.94 ± 0.03	30.50 ± 0.12	26.26 ± 0.24	6.87 ± 0.03
**11t**	3-Cl	N	12.68 ± 0.05	3.02 ± 1.12	14.58 ± 0.32	84.95 ± 3.40	67.00 ± 2.02
**11u**	4-Cl	N	8.10 ± 0.02	4.96 ± 0.18	60.66 ± 1.13	27.77 ± 1.84	2.08 ± 0.01
**11v**	3,4-*Di*Cl	N	10.37 ± 0.03	55.30 ± 1.82	67.70 ± 1.55	26.37 ± 0.90	68.37 ± 1.44
**11w**	3-Cl-4-Me	N	6.80 ± 0.05	9.42 ± 0.63	71.87 ± 1.10	38.45 ± 0.42	6.23 ± 0.03
**11x**	3-CF_3_	N	9.36 ± 0.06	32.93 ± 0.61	60.55 ± 0.41	44.61 ± 0.52	9.15 ± 0.02
**11y**	3-CF_3-_4-Cl	N	20.48 ± 0.11	4.37 ± 0.06	45.69 ± 0.06	71.60 ± 3.24	57.07 ± 0.61
	Sorafenib		7.06 ± 0.17	5.45 ± 0.11	19.8 ± 0.12	29.6 ± 0.31	2.81 ± 0.71

(IC_50_, µM)^a^ values were determined by MTT assay.

^a^Data are expressed as the mean ± standard deviation from the dose response curves of at least three independent experiments.

In the second series, against the MCF-7 cell line, compounds from pyridine series, including **11m**, **11n**, **11p**, **11r**, **11s**, **11w** exhibit better anticancer activity than that of sorafenib. Among them, Compound **11n** is the most active compound against four cell lines with IC_50_s values of 2.67, 6.84, 7.20 and 2.09 µM in MCF-7, SW-480, HEPG-2, and HUVEC cell lines, respectively. The electronic displacement of compounds **11l**, **11n** and even the presence of methoxy with positive mesomeric effect resulted in similar activity compared to sorafenib against HUVEC. The activity of these compounds against MCF-7 is stronger than positive control. In this series, comparing the inductive effect of halogens revealed that the most electronegative atom, fluorine, at *para* position, **11r**, resulted in less active compound compared to *para* chloro substituted compound, **11u**, except against MCF-7. The movement of methoxy from *meta* to *para* enhances the cytotoxicity of compound **11p** against all cell lines except PC-3. The preferred substituent on the phenyl moiety is methyl as the lipophilic group at the *meta* and *para* position. Compound **11l** exhibits the highest inhibitory activity against four out of five cell lines, including MCF-7, HEPG-2, SW480 and HUVEC. The introduction of chlorine to *meta* position, **11w,** led to the decreased activity compared to **11n** except the activity against PC-3. The movement of chlorine to *para* position (**11t** *vs* **11u**) increases almost 30 folds the cytotoxicity against HUVEC. Compound **11 × **with CF_3_ group at *meta* position shows a lower but not negligible cytotoxicity. From this initial screen, we selected compounds **11l**, **11m**, **11n** from pyridine series and **11b**, **11h** from phenyl series to investigate the mechanism of activity against MCF-7 and HUVEC cells. The cytotoxicity of compounds **11b**, **11h**, **11l**, **11m**, **11n** against the normal cell line (MRC5) were also determined (Table 2). The compound **11n** shows IC_50_ value of 38.1 μM and the highest selectivity index of 14. Selectivity against MCF-7 versus MRC5 range is between 3.9–14. Results indicated that these compounds have acceptable SI values and expected to be safe.

**Table 2:** In vitro antiproliferative effects (IC_50_, µM) of **11b, 11h, 11l, 11m, 11n** against MCF-7 and MRC5 cell lines^a^.

Compound	MRC5	MCF7	SI ^b^
**11b**	32.07 ± 0.92	8.22 ± 0.09	3.9
**11h**	41.21 ± 0.45	8.52 ± 0.02	4.8
**11l**	24.82 ± 0.36	2.80 ± 0.03	8.8
**11m**	33.25 ± 0.44	3.35 ± 0.01	9.9
**11n**	38.10 ± 0.81	2.67 ± 0.21	14.2

^a^Values were the means of three replicates ± standard deviation (SD).

^b^SI = Selectivity Index = IC_50_ value normal cell/IC_50_ value cancer cell.

### Apoptosis-inducing activity

The programmed cell death, apoptosis is essential in important biological processes. It is worth mentioning that disease like cancer is considered as a result of disruption in this process.

**Figure 2:**
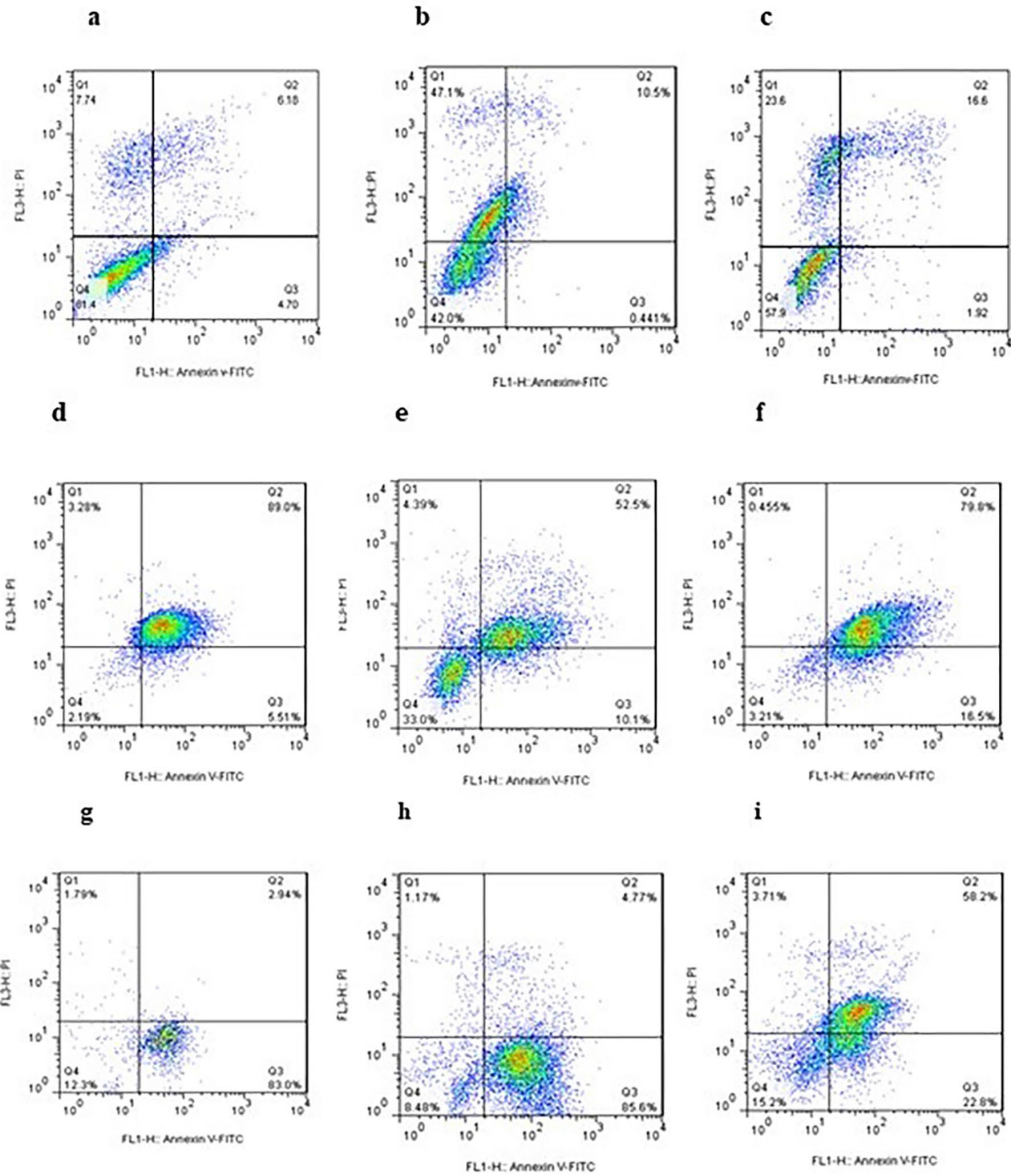
Apoptosis analysis of HUVEC and MCF-7 cells induced by **11l**, **11m**, **11**n. (**a**) DMSO, (**b**,**c**) sorafenib; (**d**–**f**) **11l**, **11m, 11n** against HUVEC; (**g**–**i**) **11l, 11m, 11n** against MCF-7. One time experiment was performed in one replicate X axis shows PI and y axis show ANNEXIN V; Q1: Necrotic cells; Q2: Late apoptotic cells; Q3: Early apoptotic cells; Q4: Live cells.

The ability of compounds **11b**, **11h**, **11l**, **11m,** and **11n** at IC_50_ concentration in inducing apoptosis on MCF-7, HUVEC cell lines was evaluated^27^. The experiment was conducted by flow cytometry analysis of pigmented cells with propidium iodide (PI) and annexin V-fluorescein isothiocyanate (annexine V-FITC). DMSO and sorafenib were used as the negative and positive control, respectively. The results are depicted in Fig. 2 showing the percentages of necrotic, apoptotic and live cells. The higher percentage of total apoptosis in HUVEC cells compared to MCF-7 is observed for compound **11n**. The percentage of early apoptosis for **11l**, **11m**, **11n** are 5.51%, 10.1%, 16.5% and 89.0%, 52.5%, 79.8% of late apoptosis in HUVEC cells. These numbers in MCF-7 cell line are 4.77%, 2.94%, 58.2% and 85.6%, 83.0% and 22.8% for late and early apoptosis, respectively. The apoptosis induced by compound **11n** is much greater than that of sorafenib. While in **11a–k** series, both selected compounds, **11b, 11h**, are unable to induce apoptosis and cells remained live in HUVEC and MCF-7 (The figure is inserted in supporting file). The obtained results and apoptotic cell population confirmed that **11l**, **11m**, **11n** show significant activities in this order **11n > 11l > 11m** against HUVEC cell line while against MCF-7 the order is reversed **11m > 11l > 11n**.

### The effect of compound 11n on the Caspase-3 level

The activity of caspase (cysteinyl-aspartate-specific proteases) as intracellular cell death enzymes has been characterized. So, the inhibition of caspase-3 is considered enough to block or even slow down the process^28–31^. By using ELISA assay kit, the bioluminescent intensity of caspase-3 was assessed time-dependently in MCF-7 cells treated with compound **11n** at 2.67 μM for 24 h. As shown in Table 3, the treatment of MCF-7 cells with this compound boosted the caspase-3 level by 5.2-fold compared to sorafenib.

**Table 3:** The concentration of caspase-3 in MCF-7 cells after treatment with compound **11n** for 24 h.

Compound	Conc. (μΜ)	Casp3 conc. (ng/mL)	FLD
11n	2.67	3.63 ± 0.52	5.2
Sorafenib	7.06	4.54 ± 0.25	6.5
Control	–	0.70 ± 0.14	1

### Cell cycle arrest

**Figure 3:**
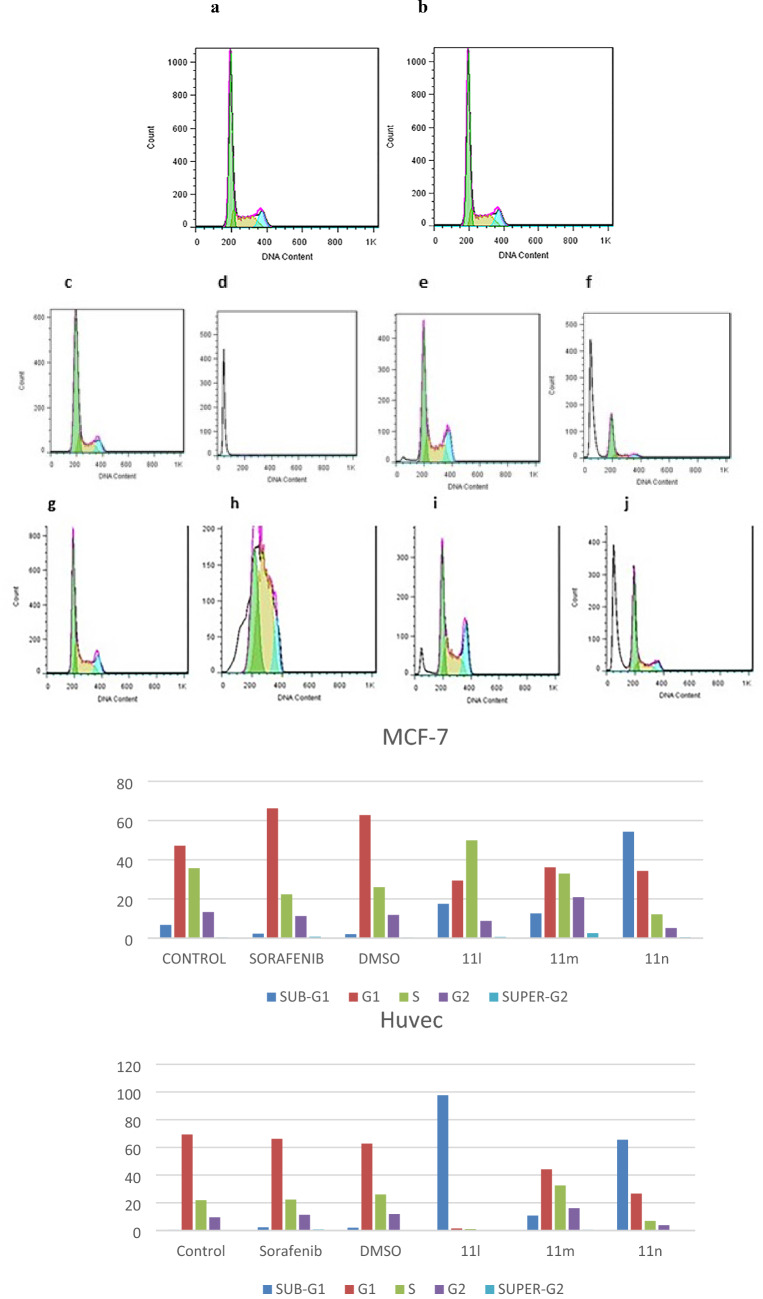
Flow cytometric analysis of cells cycle distribution on HUVEC and MCF-7 cells (x axis: count, y axis: DNA content) G1: Green, S: Yellow, G2: Blue, sub-G1: Without color, One time experiment was performed in one replicate; (**a**,**b**) treated with DMSO, sorafenib; (**c**,**g**) Non-treated cells as control group against HUVEC, MCF-7; (**d**–**f**) treated with **11l, 11m, 11n** in HUVEC; (C) treated with **11l, 11m, 11n** against MCF-7 at IC_50_ concentration.

To further understand the antitumor mechanism of compounds, the impact of the most active compounds on cell cycle progression against HUVEC and MCF-7 were examined at IC_50_ concentration by Annexin V-FITC/PI (Propidium iodide) dual staining assay (The figures of **11b**, **11 h** are inserted in Supporting file)^28^. As shown in Fig. 3, the analysis of cell cycle revealed that compound **11 l** increases % the cells in G0 phase to 97.6%. It was observed that compound **11n** demonstrates elevation in the cell population of the examined cell line, MCF-7, in G0 phase and arrests the cell growth in G0 phase. The percentage of G0 cell is increased to 54.3% and 65.5% in MCF-7 and HUVEC cell lines, respectively. Whereas non-treated cells and sorafenib show 2.0 and 2.2% in this phase. We can conclude that compound **11n** induces cellular apoptosis in G0 Phase. An increase in sub-G1 area is an indicative of apoptosis. Compounds **11m**, **11l** increase the percentage of cell population in the S phase of the cell cycle to 32.9% and 49.8%. These results suggest that they could induce cancer cell cycle arrest (MCF-7) at the S phase.

### Anti-angiogenic activity

**Figure 4:**
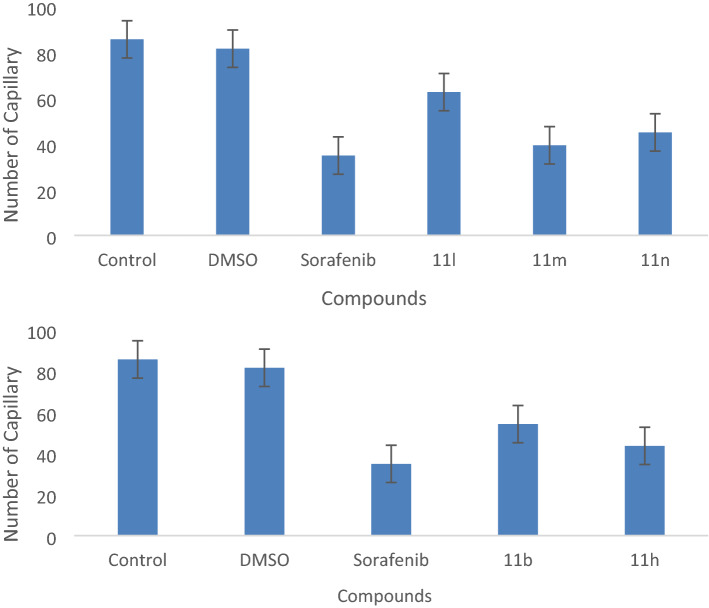
Average number of blood vessels treated with selected compounds at IC_50_ concentration. CAM photographs of selected compounds.**11n**, Sorafenib, and negative control.

**Figure 5:**
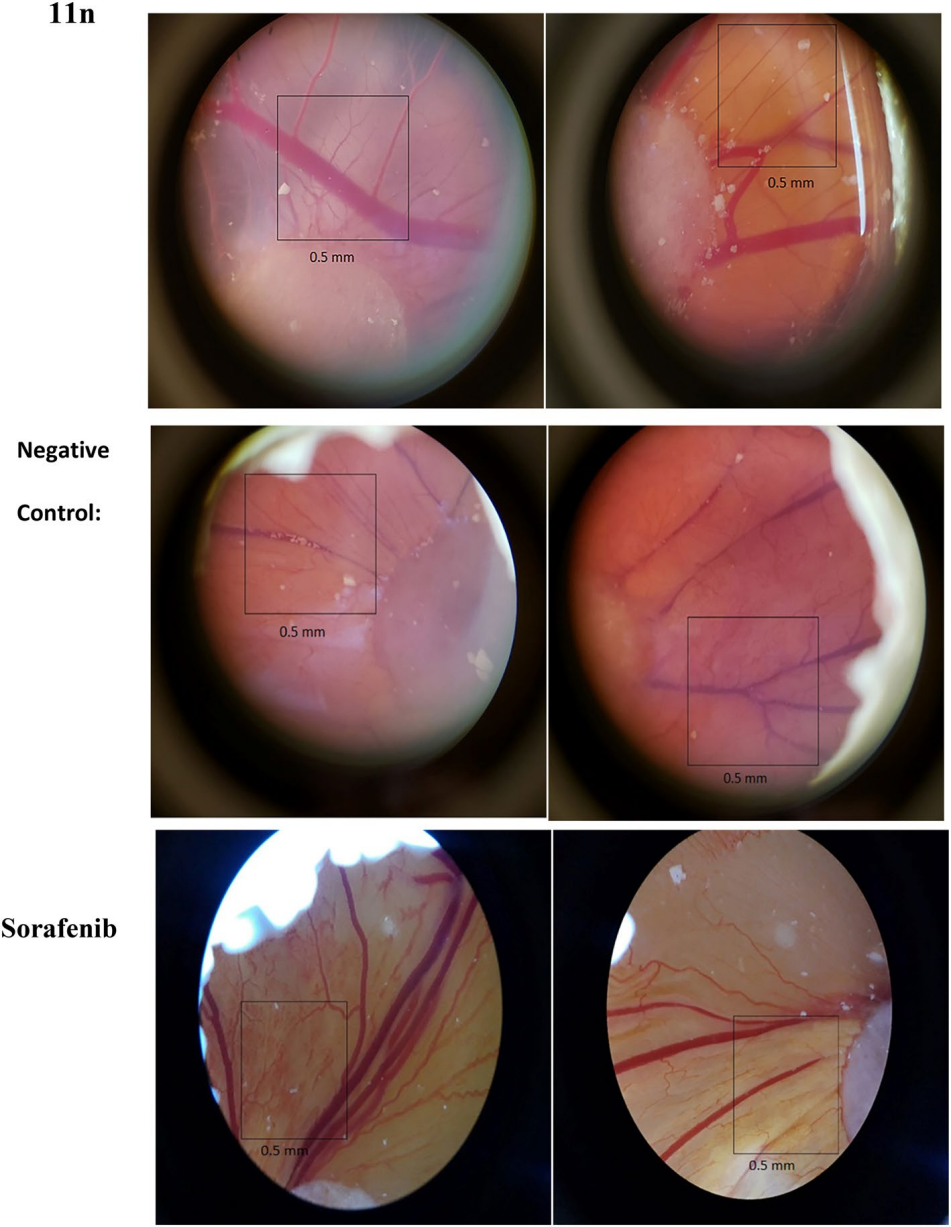
The images of angiogenesis inhibitory effect of **11n** (first row), negative control (second row), Sorafenib (third row) on CAM.

Since VEGF has been shown to be a strong cell proliferation, and migration inducer, angiogenesis inhibition via VEGF-mediated signaling has been considered as a treatment approach. The hydrogen bonding between NH groups of urea and target proteins were known to give rise to better inhibition of an important growth factor receptor (VEGFR-2) involved in the process of angiogenesis. Thus, the chick chorioallantoic membrane (CAM) assay was utilized as an *in-vivo* technique to evaluate the anti-vascular effects. Therefore, the anti-angiogenic strength of compounds were investigated by a cheap and simple CAM assay (Figs. 4, 5)^32,33^. The anti-angiogenic strength was measured and compared with DMSO (control) and Sorafenib as a positive control. Compounds **11h, 11m, 11n** exhibit slightly weaker inhibitory effects on growing CAM compared to the positive control.

### Molecular docking study

**Figure 6:**
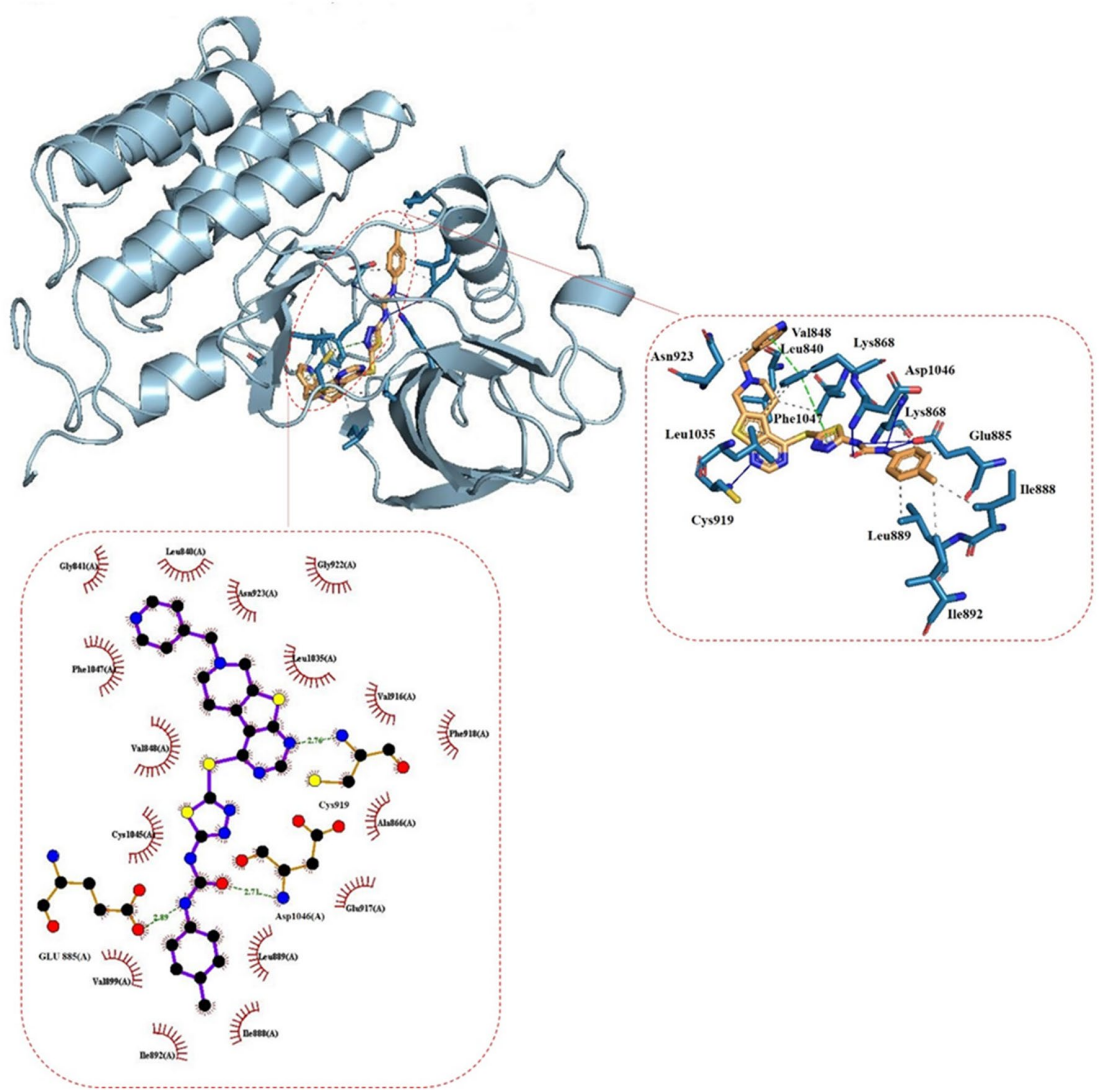
Two-dimensional (2D) and three-dimensional (3D) diagrams of compound **11n** in the active site of VEGFR-2 receptor. The hydrophobic interactions, hydrogen bonds and π-stacking interactions are indicated as black-dotted, blue, and green-dashed lines, respectively. The white spheres indicate center of aromatic rings.

To understand the structure–activity relationship, the compound **11n** was docked into the catalytic site of VEGFR-2 protein (PDB code: 3WZE)^34^. To elucidate the binding interactions of compound **11n** in the active site region of VEGFR-2, molecular docking simulation was carried out. The results are shown in Fig. 6. It can be seen that there are three types of interactions including hydrogen bond interactions, hydrophobic interactions, and π-stacking interactions. Four hydrogen bonds are formed between Lys 868, Glu 885, Asp 1046, and Cys 919 residues and the urea and pyrimidine part of potent compound, respectively. There are several hydrophobic interactions between Leu 840, Val 848, Glu 885, Ile 888, Leu 889, Ile 892, Leu 1035, Asp 1046, Phe 1047, Asn 923 residues and methyl phenyl, fused region and pyridine part of potent compound. There are two π-stacking interactions with Phe 1047 residue through thiadiazole and pyridine rings of potent compound. Thus, the docking results proposed that the potent compound **11n** can be regarded as a potential VEGFR-2 inhibitor.

### Western blot analysis

**Figure 7:**
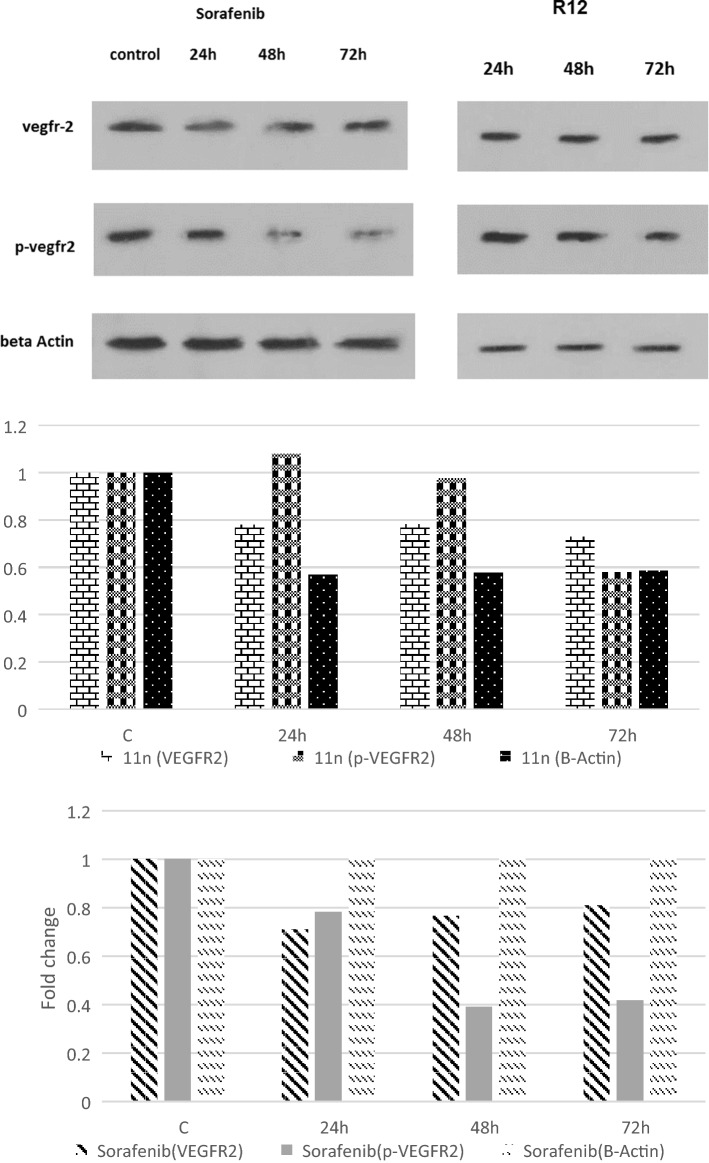
Representative western blot and bar chart analysis showing expression of VEGFR-2, P-VEGFR-2 against Sorafenib, **11n** (samples were run on a separate PVDF for detecting each antibody).

Western blot was performed to investigate the effect of the most potent compound **11n** on VEGFR-2 protein’s phosphorylation in VEGFR-2 overexpressed HUVECs cell membrane^35^. It was is observed that, after 48 and 72 h, the phosphorylation of VEGFR-2 decreases compared to the positive control (Fig. 7).

### Conclusion

In summary, we have successfully synthesized a series of tetrahydropyridothienopyrimdines with antiproliferative and anti-angiogenic properties. Among the synthesized compounds, we found that compound **11n** shows good cytotoxic activities Cellular mechanistic studies of compound **11n** disclosed that it prompts apoptosis, cell growth cessation at G0 phase and it reinforces apoptosis via activation of caspase-3. The anti-angiogenic strength and western-blot analysis were performed to investigate the mechanism of action. Hydrogen bonding with urea and pyrimidine part with key amino acids and π-stacking interactions of thiadiazole with Phe 1047 showed that compound **11n** with proper binding modes can be probably used as principle templates for further investigations. Mechanistic studies and low toxicity profile showed that this core structure has the potential value as a promising candidate for further modification to find novel therapeutic agents.

## Methods

### Chemistry

All chemicals were prepared from Merck and Sigma. Nicolet FT-IR Magna 550 spectrometer was used to record Infrared spectra. The Bruker FT-500, 300 MHz spectrometers using DMSO-*d*_*6*_, CDCl_3_ and TMS as the internal standard were used to capture ^1^H NMR spectra and ^13^C NMR.

### Synthesis of 1-substituted piperidin-4-one (3)

A mixture of piperidin-4-one hydrochloride **1** (15 mmol) with K_2_CO_3_ (20 mmol) in DMF (20 mL) was stirred at 80 °C. After 30 min, 4-(chloromethyl)pyridine or benzylchloride **2** (15 mmol) was added to the mixture which was heated for further 5 h. Upon completion, the mixture was cooled to the ambient temperature, water (30 mL) was added and extracted with EtOAc (3 × 45 mL and brine and the solvent was removed under the reduced pressure. The residue was purified by column chromatography using *n*-hexane/EtOAc (6:1) as eluent to afford compound **3** as yellow oil in 70% yield.

### (Pyridin-4-ylmethyl)piperidin-4-one (3b)

^1^H NMR (500.1 MHz, DMSO-*d*_6_): *δ* 8.53 (d, *J* = 5.3 Hz, 2H, 2CH), 7.38 (d, *J* = 5.3 Hz, 2H, 2CH), 3.66 (s, 2H,CH_2_N), 2.70 (t, *J* = 5.9 Hz, 4H, 2CH_2_CH_2_N), 2.37 (t, *J* = 5.9 Hz, 4H, 2CH_2_CH_2_N).

### Synthesis of 6-substituted ethyl 2-amino-4,5,6,7-tetrahydrothieno[2,3-c]pyridine-3-carboxylate (4a,b)

A solution of previously synthesized compound **3** (15 mmol), ethyl cyanoacetate (16.5 mmol) and morpholine (30 mmol) was heated in EtOH (20 mL) under reflux conditions for 30 min. Then, sulfur (18 mmol) was added gradually over 5 min. The resulting mixture was refluxed for further 3 h. After the consumption of starting materials according to the TLC analysis, the reaction was cooled to the ambient temperature and stirred overnight. The precipitated product was filtered and washed with diethyl ether (5 mL) to afford pure compound **4** as a yellow solid.

**Ethyl 2-amino-6-benzyl-4,5,6,7-tetrahydrothieno[2,3-*c*]pyridine-3-carboxylate (4a):** The compound was characterized according to the literature^36^.

**Ethyl 2-amino-6-(pyridin-4-ylmethyl)-4,5,6,7-tetrahydrothieno[2,3-*c*]pyridine-3-carboxylate (4b): **^1^H NMR (500.1 MHz, DMSO-*d*_6_): *δ* 8.51 (d, *J* = 5.8 Hz, 2H, 2CH), 7.35 (d, *J* = 5.8 Hz, 2H, 2CH), 3.73–3.68 (m, 4H, CH_2_CH_2_N), 3.66 (s, 2H, NH_2_), 3.34 and 3.32 (2 s, 4H, 2CH_2_N), 3.14 (q, *J* = 7.1 Hz, 2H, OC*H*_2_CH_3_), 1.23 (t, *J* = 7.1 Hz, 3H, CH_3_).

### Synthesis of 7-Substituted-5,6,7,8-tetrahydropyrido[4',3':4,5]thieno[2,3-d]pyrimidin-4(3H)-one (5)

To the solution of compound **4** (15 mmol) in DMF (25 mL) was magnetically stirred at 100 °C, formamidine acetate (120 mmol) was added within 30 min. The mixture was heated for further 16 h. Then, it was poured gradually into the iced water (100 mL), and the precipitated solid was separated by filtration to obtain pure compound **5** as the pale yellow solid in 80% yield.

### 7-(Pyridin-4-ylmethyl)-5,6,7,8-tetrahydropyrido[4',3':4,5]thieno[2,3-d]pyrimidin-4(3H)-one (5b)

^1^H NMR (500.1 MHz, DMSO-*d*_6_): *δ* 8.52 (d, *J* = 5.8 Hz, 2H, 2CH), 8.02 (s, 1H, CH), 7.38 (d, *J* = 5.8 Hz, 2H, 2CH), 3.74 and 3.65 (2 s, 4H, 2CH_2_N), 2.95 (t, *J* = 5.2 Hz, 2H, CH_2_C*H*_2_N), 2.76 (t, *J* = 5.4 Hz, 2H, C*H*_2_CH_2_N).

### Synthesis of 7- Substituted-4-chloro-5,6,7,8-tetrahydropyrido[4',3':4,5]thieno[2,3-d]pyrimidine (6)

To the mixture of compound **5** (10 mmol) and DBU (12 mmol) under the ice-bath condition, POCl_3_ (8 mL) was added dropwise. Afterwards, the reaction mixture was warmed to 70 °C and stirred for further 7 h. After the complete consumption of compound **5**, the mixture was neutralized with NaHCO_3_ saturated solution and extracted, and washed with brine. After removal of the solvent undervacuum, the desired product **6** was obtained as a yellow solid in 80% yield.

### Synthesis of 5-amino-1,3,4-thiadiazole-2-thiol (8)

To the mixture of thiosemicarbazide **7** (15 mmol) and Na_2_CO_3_ (15 mmol) in absolute ethanol (15 mL), carbon disulfide (30 mmol) in EtOH (5 mL) was added dropwise and the obtained mixture was heated under reflux conditions for 8 h. As the completion of reaction was confirmed by TLC, the solvent was removed under reduced pressure. The residue was diluted through the addition of water (50 mL), then, concentrated HCl solution was added dropwise to precipitate compound **8** as yellow solid in 80% yield.

### Synthesis of 1-(5-mercapto-1,3,4-thiadiazol-2-yl)-3-arylurea (10)

A mixture of compound **8** (10 mmol), substituted phenyl isocyanate derivative **9** (10 mmol), and DIPEA (12 mmol) was dissolved in dry DCM and stirred under an argon atmosphere. After the completion of reaction indicated by TLC analysis, the solvent was removed under reduced pressure to obtain white precipitate as pure desirable compound **10**.

### General procedure for the synthesis of final compounds (11a-y)

A mixture of desired urea derivative **10** (1 mmol) and DBU (1 mmol) in EtOH (5 mL) was heated under reflux conditions for 10 min, then compound **6** (1 mmol) was added gradually within 5 min. The mixture was refluxed for further 5 h. Afterwards, it was poured gradually into the iced water (10 mL), formed precipitate was filtered and washed with diethyl ether to obtain the desirable product 11 within 70–90% yields.

#### (5-((7-Benzyl-5,6,7,8-tetrahydropyrido[4',3':4,5]thieno[2,3-d]pyrimidin-4-yl)thio)-1,3,4-thiadiazol-2-yl)-3-phenylurea (11a)

C_25_H_21_N_7_OS_3_, White solid, mp 230–232 °C. IR (KBr) (*ν*_max_/cm^–1^): 3318 and 2923 (2NH), 1637 (C = O), 1528, 1494, 1421, 1384, 1312, 1245, 1106, 1073, 1024, 872, 834, 784, 696, 640. ^1^H NMR (300.1 MHz, DMSO-*d*_6_):* δ* 10.71 and 9.50 (2 s, 2H, 2NH), 8.69 (s, 1H, CH), 7.78 (d, *J* = 8.0 Hz, 2H, 2CH), 7.42–7.15 (m, 7H, 7CH), 6.88 (t, *J* = 7.3 Hz, 1H, CH), 3.73 and 3.72 (2 s, 4H, 2CH_2_N), 3.17–3.02 (m, 2H, CH_2_C*H*_2_N), 2.87 (t, *J* = 5.4 Hz, 2H, C*H*_2_CH_2_N). ^13^C NMR (75.5 MHz, DMSO-*d*_6_): *δ* 165.7 (C = O); 165.4, 160.4, 158.1, 151.7, 143.0, 141.1, 138.0, 135.4, 128.8, 128.6, 128.4, 127.2, 126.7, 125.4, 121.0, 117.9; 60.4, 53.4 and 47.9 (3CH_2_N); 23.3 (*C*H_2_CH_2_N).

#### (5-((7-Benzyl-5,6,7,8-tetrahydropyrido[4',3':4,5]thieno[2,3-d]pyrimidin-4-yl)thio)-1,3,4-thiadiazol-2-yl)-3-(m-tolyl)urea (11b)

C_26_H_23_N_7_OS_3_Yellow solid, mp 138–139 °C. IR (KBr) (*ν*_max_/cm^–1^): 3146 and 2922 (2NH), 1634 (C = O), 1598, 1546, 1493, 1422, 1307, 1252, 1202, 1110, 1079, 978, 891, 830, 775, 740, 697, 639. ^1^H NMR (500.1 MHz, DMSO):* δ* 10.33 and 9.55 (2 s, 2H, 2NH), 8.75 and 7.61 (2 s, 2H, 2CH), 7.47 (d, *J* = 7.8 Hz, 1H, CH), 7.41 (d, *J* = 7.4 Hz, 2H, 2CH), 7.37 (t, *J* = 7.5 Hz, 2H, 2CH), 7.30 (t, *J* = 7.2 Hz, 1H, CH), 7.14 (t, *J* = 7.4 Hz, 1H, CH), 6.76 (d, *J* = 7.4 Hz, 1H, CH), 3.79 and 3.77 (2 s, 4H, 2CH_2_N), 3.20–3.09 (m, 2H, CH_2_C*H*_2_N), 2.92 (t, *J* = 4.6 Hz, 2H, C*H*_2_CH_2_N), 2.28 (s, 3H, CH_3_). ^13^C NMR (125.1 MHz, DMSO-*d*_6_): *δ* 166.3 (C = O); 165.9, 160.3, 156.9, 153.5, 152.1, 140.8, 138.5, 138.2, 136.1, 129.3, 128.9, 128.8, 127.6, 127.2, 125.8, 122.7, 119.2, 115.9; 60.8, 53.9 and 48.4 (3CH_2_N); 23.8 (*C*H_2_CH_2_N); 19.3 (CH_3_).

#### (5-((7-Benzyl-5,6,7,8-tetrahydropyrido[4',3':4,5]thieno[2,3-d]pyrimidin-4-yl)thio)-1,3,4-thiadiazol-2-yl)-3-(p-tolyl)urea (11c)

C_26_H_23_N_7_OS_3_, Pale yellow solid, mp 293–295 °C. IR (KBr) (*ν*_max_/cm^–1^): 3375 and 2858 (2NH), 1652 (C = O), 1582, 1530, 1493, 1444, 1409, 1382, 1313, 1236, 1203, 1141, 1115, 1073, 975, 874, 809, 740, 700, 634. ^1^H NMR (300.1 MHz, DMSO-*d*_6_):* δ* 10.55 and 9.71 (2 s, 2H, 2NH), 8.75 (s, 1H, CH), 7.50–7.19 (m, 7H, 7CH), 7.10 (d, *J* = 7.1 Hz, 2H, 2CH), 3.75 and 3.73 (2 s, 4H, 2CH_2_N), 3.20–2.97 (m, 2H, CH_2_C*H*_2_N), 2.96–2.67 (m, 2H, C*H*_2_CH_2_N), 2.23 (s, 3H, CH_3_).

#### (5-((7-Benzyl-5,6,7,8-tetrahydropyrido[4',3':4,5]thieno[2,3-d]pyrimidin-4-yl)thio)-1,3,4-thiadiazol-2-yl)-3-(4-methoxyphenyl)urea (11d)

C_26_H_23_N_7_O_2_S_3_, White solid, mp 280–282 °C. IR (KBr) (*ν*_max_/cm^–1^): 3198 and 2928 (2NH), 1646 (C = O), 1590, 1541, 1510, 1452, 1411, 1386, 1316, 1237, 1180, 1142, 1111, 1081, 1036, 829, 738, 700, 636. ^1^H NMR (300.1 MHz, DMSO-*d*_6_):* δ* 10.36 and 9.50 (2 s, 2H, 2NH), 8.71 (s, 1H, CH), 7.60 (d, *J* = 7.7 Hz, 2H, 2CH), 7.50–7.18 (m, 5H, 5CH), 6.95–6.76 (m, 2H, 2CH), 3.88–3.56 (s, 7H, 2CH_2_N and OCH_3_), 3.19–2.96 (m, 2H, CH_2_C*H*_2_N), 2.94–2.72 (m, 2H, C*H*_2_CH_2_N). ^13^C NMR (75.5 MHz, DMSO-*d*_6_): *δ* 165.8 (C = O); 165.3, 159.0, 154.8, 154.6, 151.5, 137.9, 135.7, 132.5, 128.8, 128.3, 127.1, 126.6, 125.2, 120.4, 120.1, 113.9; 60.4 (CH_2_N); 55.1 (OCH_3_); 53.4 (CH_2_N); 47.8 (CH_2_); 23.3 (*C*H_2_CH_2_N).

#### (5-((7-Benzyl-5,6,7,8-tetrahydropyrido[4',3':4,5]thieno[2,3-d]pyrimidin-4-yl)thio)-1,3,4-thiadiazol-2-yl)-3-(3-fluorophenyl)urea (11e)

C_25_H_20_FN_7_OS_3_, Yellow solid, mp 144–146 °C. IR (KBr) (*ν*_max_/cm^–1^): 3324 and 2926 (2NH), 1615 (C = O), 1532, 1493, 1420, 1386, 1306, 1210, 1140, 1110, 1073, 1026, 970, 944, 867, 831, 742, 699, 641. ^1^H NMR (500.1 MHz, DMSO-*d*_6_ + CDCl_3_):* δ* 11.11 and 9.49 (2 s, 2H, 2NH), 8.72 (s, 1H, CH), 7.73 (d, *J* = 7.2 Hz, 1H, CH), 7.51 (d, *J* = 4.7 Hz, 1H, CH), 7.42–7.15 (m, 6H, 6CH + CDCl_3_), 6.77 (t, *J* = 4.8 Hz, 1H, CH), 3.75 and 3.73 (2 s, 4H, 2CH_2_N), 3.20–3.08 (m, 2H, CH_2_C*H*_2_N), 2.93–2.75 (m, 2H, C*H*_2_CH_2_N).

#### (5-((7-Benzyl-5,6,7,8-tetrahydropyrido[4',3':4,5]thieno[2,3-d]pyrimidin-4-yl)thio)-1,3,4-thiadiazol-2-yl)-3-(4-fluorophenyl)urea (11f)

C_25_H_20_FN_7_OS_3_, Yellow solid, mp 166–168 °C. IR (KBr) (*ν*_max_/cm^–1^): 3165 and 2930 (2NH), 1619 (C = O), 1536, 1550, 1452, 1390, 1299, 1204, 1153, 1116, 1072, 1027, 875, 832, 741, 700, 638. ^1^H NMR (500.1 MHz, DMSO-*d*_6_):* δ* 11.39 and 9.21 (2 s, 2H, 2NH), 8.81 (s, 1H, CH), 7.53 (dd, *J* = 8.9, 4.8 Hz, 2H, 2CH), 7.46–7.34 (m, 4H, 4CH), 7.31 (t, *J* = 7.0 Hz, 1H, CH), 7.19 (t, *J* = 8.9 Hz, 2H, 2CH), 3.81 and 3.79 (2 s, 4H, 2CH_2_N), 3.17 (t, *J* = 4.5 Hz, 2H, CH_2_C*H*_2_N), 2.93 (t, *J* = 4.5 Hz, 2H, C*H*_2_CH_2_N). 

#### 1-(5-((7-Benzyl-5,6,7,8-tetrahydropyrido[4',3':4,5]thieno[2,3-d]pyrimidin-4-yl)thio)-1,3,4-thiadiazol-2-yl)-3-(4-chlorophenyl)urea (11g)

C_25_H_20_ClN_7_OS_3_, White solid, mp 220–222 °C. IR (KBr) (*ν*_max_/cm^–1^): 3138 and 2922 (2NH), 1639 (C = O), 1527, 1492, 1426, 1391, 1305, 1243, 1205, 1111, 1025, 829, 742, 699, 620. ^1^H NMR (500.1 MHz, DMSO-*d*_*6*_ + CDCl_3_):* δ* 10.70 and 9.51 (2 s, 2H, 2NH), 8.71 (s, 1H, CH), 7.77 (d, *J* = 7.2 Hz, 2H, 2CH), 7.52–7.18 (m, 7H, 7CH + CDCl_3_), 3.76 and 3.74 (2 s, 4H, 2CH_2_N), 3.20–3.08 (m, 2H, CH_2_C*H*_2_N), 2.89 (t, *J* = 4.8 Hz, 2H, C*H*_2_CH_2_N).

#### (5-((7-Benzyl-5,6,7,8-tetrahydropyrido[4',3':4,5]thieno[2,3-d]pyrimidin-4-yl)thio)-1,3,4-thiadiazol-2-yl)-3-(3,4-dichlorophenyl)urea (11h)

C_25_H_19_Cl_2_N_7_OS_3_,White solid, mp 190–192 °C. IR (KBr) (*ν*_max_/cm^–1^): 3187 and 2924 (2NH), 1639 (C = O), 1524, 1488, 1424, 1379, 1299, 1245, 1110, 1025, 872, 823, 742, 699, 623. ^1^H NMR (300.1 MHz, DMSO-*d*_6_):* δ* 10.40 and 9.49 (2 s, 2H, 2NH), 8.67 and 8.11 (2 s, 2H, 2CH), 7.58 (d, *J* = 7.7 Hz, 1H, CH), 7.45–7.20 (m, 6H, 6CH), 3.76 and 3.73 (2 s, 4H, 2CH_2_N), 3.18–3.06 (m, 2H, CH_2_C*H*_2_N), 2.87 (br. s, 2H, C*H*_2_CH_2_N). ^13^C NMR (75.5 MHz, DMSO-*d*_6_): *δ* 165.6 (C = O); 165.4, 151.8, 144.5, 141.8, 138.0, 135.1, 130.7, 130.6, 130.1, 128.8, 128.4, 127.2, 126.7, 125.5, 123.5, 120.5, 120.4, 117.9; 60.4, 53.4 and 47.9 (3CH_2_N); 23.3 (*C*H_2_CH_2_N). ESI–MS *m/ z*: 599.38 [M + H]^+^.

#### (5-((7-Benzyl-5,6,7,8-tetrahydropyrido[4',3':4,5]thieno[2,3-d]pyrimidin-4-yl)thio)-1,3,4-thiadiazol-2-yl)-3-(3-chloro-4-methylphenyl)urea (11i)

C_26_H_22_ClN_7_OS_3_, White solid, mp 155–157 °C. IR (KBr) (*ν*_max_/cm^–1^): 3248 and 2918 (2NH), 1639 (C = O), 1573, 1525, 1495, 1423, 1385, 1302, 1246, 1201, 1114, 1035, 873, 817, 741, 698, 636. ^1^H NMR (500.1 MHz, DMSO-*d*_6_):* δ* 11.08 and 9.63 (2 s, 2H, 2NH), 8.74 and 7.99 (2 s, 2H, 2CH), 7.55 (d, *J* = 8.2 Hz, 1H, CH), 7.44–7.33 (m, 4H, 4CH), 7.29 (t, *J* = 7.0 Hz, 1H, CH), 7.21 (d, *J* = 8.4 Hz, 1H, 1CH), 3.76 and 3.74 (2 s, 4H, 2CH_2_N), 3.15 (t, *J* = 5.0 Hz, 2H, CH_2_C*H*_2_N), 2.93–2.65 (m, 2H, C*H*_2_CH_2_N), 2.25 (s, 3H, CH_3_). ^13^C NMR (125.1 MHz, DMSO-*d*_6_): *δ* 166.3 (C = O); 165.8, 156.2, 152.0, 148.8, 140.1, 138.4, 136.1, 133.5, 131.5, 129.3, 128.9, 128.8, 128.3, 127.6, 127.2, 125.8, 118.5, 117.4; 60.9, 53.9 and 48.4 (3CH_2_N); 23.8 (*C*H_2_CH_2_N); 19.3 (CH_3_). ESI–MS *m/ z*: 577.36 [M + H]^+^.

#### (5-((7-Benzyl-5,6,7,8-tetrahydropyrido[4',3':4,5]thieno[2,3-d]pyrimidin-4-yl)thio)-1,3,4-thiadiazol-2-yl)-3-(3-(trifluoromethyl)phenyl)urea (11j)

C_26_H_20_F_3_N_7_OS_3_, Yellow solid, mp 181–183 °C. IR (KBr) (*ν*_max_/cm^–1^): 3124 and 2928 (2NH), 1640 (C = O), 1552, 1494, 1425, 1384, 1308, 1249, 1219, 1174, 1116, 1072, 1033, 969, 904, 837, 790, 741, 698, 657. ^1^H NMR (500.1 MHz, DMSO-*d*_6_):* δ* 11.49 and 9.53 (2 s, 2H, 2NH), 8.69 and 8.36 (2 s, 2H, 2CH), 7.95 (d, *J* = 7.3 Hz, 1H, CH), 7.47 (t, *J* = 7.3 Hz, 1H, CH), 7.40–7.14 (m, 6H, 6CH), 3.71 and 3.69 (2 s, 4H, 2CH_2_N), 3.09 (br. s, 2H, CH_2_C*H*_2_N), 2.84 (br. s, 2H, C*H*_2_CH_2_N). ^13^C NMR (125.1 MHz, DMSO-*d*_6_): *δ* 165.8 (C = O), 165.4, 159.4, 156.6, 151.6, 145.3, 141.4, 138.0, 135.6, 129.7, 129.3, 128.8, 128.3, 127.2, 126.6, 126.2, 125.2, 122.6, 121.6, 117.6; 60.5, 53.4 and 47.9 (3CH_2_N), 23.3 (*C*H_2_CH_2_N).

#### (5-((7-Benzyl-5,6,7,8-tetrahydropyrido[4',3':4,5]thieno[2,3-d]pyrimidin-4-yl)thio)-1,3,4-thiadiazol-2-yl)-3-(4-chloro-3-(trifluoromethyl)phenyl)urea (11k)

C_26_H_19_ClF_3_N_7_OS_3_, Yellow solid, mp 252–255 °C. IR (KBr) (*ν*_max_/cm^–1^): 3138 and 2924 (2NH), 1640 (C = O), 1528, 1488, 1413, 1314, 1267, 1244, 1175, 1119, 1031, 903, 833, 743, 701, 662, 630. ^1^H NMR (300.1 MHz, DMSO-*d*_6_):* δ* 11.43 and 9.49 (2 s, 2H, 2NH), 8.64 and 8.42 (2 s, 2H, 2CH), 7.93 (d, *J* = 8.8 Hz, 1H, CH), 7.43 (d, *J* = 7.9 Hz, 1H, CH), 7.38–7.15 (m, 5H, 5CH), 3.71 and 3.68 (2 s, 4H, 2CH_2_N), 3.04 (t, *J* = 6.8 Hz, 2H, CH_2_C*H*_2_N), 2.82 (t, *J* = 6.7 Hz, 2H, C*H*_2_CH_2_N). ^13^C NMR (75.5 MHz, DMSO-*d*_6_): *δ* 165.7 (C = O); 165.4, 151.6, 148.4, 140.3, 138.0, 137.3, 136.5, 135.4, 131.6, 128.8, 128.3, 128.1, 127.2, 126.7, 126.5, 125.29, 125.26, 118.5, 116.3; 60.5, 53.4 and 47.9 (3CH_2_N); 25.9 (*C*H_2_CH_2_N).

#### (5-((7-(Pyridin-4-ylmethyl)-5,6,7,8-tetrahydropyrido[4',3':4,5]thieno[2,3-d]pyrimidin-4-yl)thio)-1,3,4-thiadiazol-2-yl)-3-(o-tolyl)urea (11l)

C_25_H_22_N_8_OS_3_, Yellow solid, mp 242–244 °C. IR (KBr) (*ν*_max_/cm^–1^): 3352 and 2923 (2NH), 1645 (C = O), 1595, 1547, 1488, 1422, 1382, 1303, 1249, 1195, 1148, 1117, 1048, 962, 861, 828, 752, 639. ^1^H NMR (300.1 MHz, DMSO-*d*_6_ + CDCl_3_): *δ* 11.58 (s, 1H, NH), 8.73 (s, 1H, CH), 8.56–8.43 (m, 3H), 7.77 (d, *J* = 7.9 Hz, 1H, CH), 7.36 (d, *J* = 5.0 Hz, 2H, 2CH), 7.22–7.12 (m, 1H, CH + CDCl_3_), 7.01 (t, *J* = 7.4 Hz, 1H, CH), 4.37 (s, 1H, NH), 3.76 and 3.75 (2 s, 4H, 2CH_2_N), 3.08 (t, *J* = 4.5 Hz, 2H, CH_2_C*H*_2_N), 2.84 (t, *J* = 4.8 Hz, 2H, C*H*_2_CH_2_N), 2.24 (s, 3H, CH_3_). ^13^C NMR (75.5 MHz, DMSO-*d*_6_): *δ* 166.0 (C = O); 164.1, 155.8, 151.5, 149.7, 148.0, 147.3, 136.0, 135.8, 131.9, 130.4, 128.7, 126.7, 126.4, 125.1, 124.1, 123.7, 121.8, 119.9; 56.1, 51.5 and 48.9 (3CH_2_N); 25.9 (*C*H_2_CH_2_N); 18.6 (CH_3_).

#### (5-((7-(Pyridin-4-ylmethyl)-5,6,7,8-tetrahydropyrido[4',3':4,5]thieno[2,3-d]pyrimidin-4-yl)thio)-1,3,4-thiadiazol-2-yl)-3-(m-tolyl)urea (11m)

C_25_H_22_N_8_OS_3_, Pale yellow solid, mp 281–283 °C. IR (KBr) (*ν*_max_/cm^–1^): 3363 and 2934 (2NH), 1637 (C = O), 1545, 1492, 1416, 1384, 1307, 1251, 1215, 1145, 1107, 997, 826, 771, 740, 691, 638. ^1^H NMR (300.1 MHz, DMSO-*d*_6_):* δ* 10.86 and 9.50 (2 s, 2H, 2NH), 8.72 (s, 1H, CH), 8.51 (d, *J* = 5.5 Hz, 2H, 2CH), 7.52 (d, *J* = 8.0 Hz, 2H, 2CH), 7.38 (d, *J* = 5.5 Hz, 2H, 2CH), 7.05 (d, *J* = 7.4 Hz, 2H, CH), 3.79 and 3.76 (2 s, 4H, 2CH_2_N), 3.15 (t, *J* = 6.8 Hz, 2H, CH_2_C*H*_2_N), 3.15 (t, *J* = 6.8 Hz, 2H, CH_2_C*H*_2_N), 2.25 (s, 3H, CH_3_). ^13^C NMR (75.5 MHz, DMSO-*d*_6_): *δ* 165.8 (C = O); 165.4, 159.7, 156.3, 151.7, 149.7, 147.3, 144.8, 140.3, 137.8, 135.4, 128.5, 126.7, 125.3, 123.7, 122.4, 118.8, 115.4; 58.9, 53.4 and 47.9 (3CH_2_N); 25.9 (*C*H_2_CH_2_N); 21.4 (CH_3_).

#### (5-((7-(Pyridin-4-ylmethyl)-5,6,7,8-tetrahydropyrido[4',3':4,5]thieno[2,3-d]pyrimidin-4-yl)thio)-1,3,4-thiadiazol-2-yl)-3-(p-tolyl)urea (11n)

C_25_H_22_N_8_OS_3_, White solid, mp 170–172 °C. IR (KBr) (*ν*_max_/cm^–1^): 3346 and 2917 (2NH), 1637 (C=O), 1511, 1415, 1316, 1246, 1197, 1119, 1022, 820, 766, 740, 693, 638. ^1^H NMR (300.1 MHz, DMSO-*d*_6_): *δ* 11.02 and 9.51 (2 s, 2H, 2NH), 8.71 (s, 1H, CH), 8.51 (d, *J* = 5.1 Hz, 2H, 2CH), 7.68 (d, *J* = 8.1 Hz, 2H, 2CH), 7.38 (d, *J* = 5.1 Hz, 2H, 2CH), 7.06 (d, *J* = 8.1 Hz, 2H, 2CH), 3.78 and 3.15 (2 s, 4H, 2CH_2_N), 3.15 (br. s, 2H, CH_2_C*H*_2_N), 2.88 (t, *J* = 4.3 Hz, 2H, C*H*_2_CH_2_N), 2.22 (s, 3H, CH_3_). ^13^C NMR (75.5 MHz, DMSO-*d*_6_): *δ* 165.8 (C=O); 165.4, 159.8, 156.4, 151.7, 149.7, 147.3, 144.7, 137.9, 135.4, 130.3, 129.1, 126.6, 125.3, 123.7, 118.2; 58.9, 53.4 and 47.9 (3CH_2_N); 25.9 (*C*H_2_CH_2_N); 20.5 (CH_3_).

#### (3-Methoxyphenyl)-3-(5-((7-(pyridin-4-ylmethyl)-5,6,7,8-tetrahydropyrido[4',3':4,5]thieno[2,3-d]pyrimidin-4-yl)thio)-1,3,4-thiadiazol-2-yl)urea (11o)

C_25_H_22_N_8_O_2_S_3_, Pale yellow solid, mp 191–193 °C. IR (KBr) (*ν*_max_/cm^–1^): 3246 and 2928 (2NH), 1644 (C=O), 1599, 1547, 1495, 1419, 1385, 1309, 1221, 1177, 1152, 1109, 1078, 1028, 933, 829, 774, 742, 692, 640. ^1^H NMR (300.1 MHz, DMSO-*d*_6_):* δ* 11.25 and 9.52 (2 s, 2H, 2NH), 8.72 (s, 1H, CH), 8.51 (d, *J* = 5.2 Hz, 2H, 2CH), 7.52 (s, 1H, CH), 7.48–7.30 (m, 3H, 3CH), 7.15 (t, *J* = 8.2 Hz, 1H, CH), 6.51 (d, *J* = 8.1 Hz, 1H, CH), 3.78 and 3.75 (2 s, 4H, 2CH_2_N), 3.72 (s, 3H, OCH_3_), 3.21 (t, *J* = 7.0 Hz, 2H, CH_2_C*H*_2_N), 2.88 (t, *J* = 7.0 Hz, 2H, C*H*_2_CH_2_N). ^13^C NMR (75.5 MHz, DMSO-*d*_6_): *δ* 165.9 (C=O); 165.4, 159.7, 159.6, 156.4, 151.7, 149.7, 147.3, 145.0, 141.7, 135.4, 129.4, 126.6, 125.2, 123.7, 110.5, 107.4, 103.8; 59.0 (CH_2_N); 55.0 (OCH_3_); 53.4 and 47.9 (2 CH_2_N); 23.3 (*C*H_2_CH_2_N).

#### (4-Methoxyphenyl)-3-(5-((7-(pyridin-4-ylmethyl)-5,6,7,8-tetrahydropyrido[4',3':4,5]thieno[2,3-d]pyrimidin-4-yl)thio)-1,3,4-thiadiazol-2-yl)urea (11p)

C_25_H_22_N_8_O_2_S_3_, Orange solid, mp 169–171 °C. IR (KBr) (*ν*_max_/cm^–1^): 3381 and 2898 (2NH), 1643 (C=O), 1598, 1543, 1509, 1417, 1387, 1325, 1238, 1207, 1177, 1138, 1068, 1029, 972, 828, 797, 763, 740, 635. ^1^H NMR (300.1 MHz, DMSO-*d*_6_):* δ* 9.90 and 9.50 (2 s, 2H, 2NH), 8.74 (s, 1H, CH), 8.51 (d, *J* = 5.2 Hz, 2H, 2CH), 7.51 (d, *J* = 8.8 Hz, 2H, 2CH), 7.38 (d, *J* = 5.2 Hz, 2H, 2CH), 6.88 (d, *J* = 8.8 Hz, 2H, 2CH), 3.79 and 3.76 (2 s, 4H, 2CH_2_N), 3.70 (s, 3H, OCH_3_), 3.18–3.09 (m, 2H, CH_2_C*H*_2_N), 2.94–2.78 (m, 2H, C*H*_2_CH_2_N). ^13^C NMR (75.5 MHz, DMSO-*d*_6_): *δ* 166.0 (C = O); 165.4, 158.6, 155.0, 153.1, 151.5, 149.7, 147.4, 147.3, 135.7, 131.9, 126.6, 125.2, 123.7, 120.5, 114.0; 58.9 (CH_2_N); 55.2 (OCH_3_); 53.4 and 48.8 (2CH_2_N); 25.9 (*C*H_2_CH_2_N).

#### (3-Fluorophenyl)-3-(5-((7-(pyridin-4-ylmethyl)-5,6,7,8-tetrahydropyrido[4',3':4,5]thieno[2,3-d]pyrimidin-4-yl)thio)-1,3,4-thiadiazol-2-yl)urea (11q)

C_24_H_19_FN_8_OS_3_, Yellow solid, mp 243–246 °C. IR (KBr) (*ν*_max_/cm^–1^): 3237 and 2924 (2NH), 1638 (C=O), 1599, 1492, 1385, 1314, 1245, 1137, 1107, 1024, 987, 943, 867, 828, 769, 686, 640. ^1^H NMR (500.1 MHz, DMSO-*d*_6_): *δ* 10.57 and 9.97 (2 s, 2H, 2NH), 8.69 (s, 1H, CH), 8.52 (d, *J* = 5.0 Hz, 2H, 2CH), 7.43 (d, *J* = 4.5 Hz, 1H, CH), 7.39 (d, *J* = 5.0 Hz, 2H, 2CH), 7.23 (dd, *J* = 9.2, 4.6 Hz, 1H, CH), 6.66 (t, *J* = 4.9 Hz, 1H, CH), 3.80 and 3.78 (2 s, 4H, 2CH_2_N), 3.22 (t, *J* = 4.8 Hz, 2H, CH_2_C*H*_2_N), 2.89 (t, *J* = 4.9 Hz, 2H, C*H*_2_CH_2_N). ESI–MS *m/ z:* 550.14 [M + H]^+^.

#### (4-Fluorophenyl)-3-(5-((7-(pyridin-4-ylmethyl)-5,6,7,8-tetrahydropyrido[4',3':4,5]thieno[2,3-d]pyrimidin-4-yl)thio)-1,3,4-thiadiazol-2-yl)urea (11r)

C_24_H_19_FN_8_OS_3_, White solid, mp 201–203 °C. IR (KBr) (*ν*_max_/cm^–1^): 3308 and 2934 (2NH), 1638 (C=O), 1599, 1549, 1504, 1418, 1314, 1252, 1206, 1111, 1023, 841, 760, 697, 636. ^1^H NMR (300.1 MHz, DMSO-*d*_6_):* δ* 10.67 and 9.54 (2 s, 2H, 2NH), 8.75 (s, 1H, CH), 8.55 (d, *J* = 5.4 Hz, 2H, 2CH), 7.76 (dd, *J* = 8.7, 4.9 Hz, 2H, 2CH), 7.42 (d, *J* = 5.4 Hz, 2H, 2CH), 7.11 (t, *J* = 8.2 Hz, 2H, 2CH), 3.84 and 3.81 (2 s, 4H, 2CH_2_N), 3.20 (br. s, 2H, CH_2_C*H*_2_N), 2.93 (t, *J* = 4.2 Hz, 2H, C*H*_2_CH_2_N).

#### (2-Chlorophenyl)-3-(5-((7-(pyridin-4-ylmethyl)-5,6,7,8-tetrahydropyrido[4',3':4,5]thieno[2,3-d]pyrimidin-4-yl)thio)-1,3,4-thiadiazol-2-yl)urea (11s)

C_24_H_19_ClN_8_OS_3_, White solid, mp 229–231 °C. IR (KBr) (*ν*_max_/cm^–1^): 3357 and 2924 (2NH), 1648 (C=O), 1598, 1543, 1490, 1435, 1384, 1299, 1235, 1198, 1148, 1074, 1003, 960, 830, 751, 640. ^1^H NMR (300.1 MHz, DMSO-*d*_6_):* δ* 11.83 (s, 1H, NH), 8.74 (s, 1H, NH), 8.72 (s, 1H, CH), 8.52 (d, *J* = 4.9 Hz, 2H, 2CH), 8.10 (d, *J* = 8.2 Hz, 1H, 1CH), 8.00 (s, 1H, CH), 7.37 (d, *J* = 4.9 Hz, 2H, 2CH), 7.31 (t, *J* = 7.9 Hz, 1H, CH), 6.95 (d, *J* = 7.9 Hz, 1H, CH), 3.76 and 3.75 (2 s, 4H, 2CH_2_N), 3.12 (br. s, 2H, C*H*_2_CH_2_N), 2.85 (br. s, 2H, CH_2_C*H*_2_N). ^13^C NMR (75.5 MHz, DMSO-*d*_6_): *δ* 166.0 (C = O); 163.6, 157.7, 151.4, 151.3, 149.7, 149.2, 135.8, 134.6, 129.4, 127.8, 126.5, 125.1, 124.8, 123.7, 122.8, 121.7; 58.9, 56.1 and 48.8 (3CH_2_N), 25.9 (*C*H_2_CH_2_N). ESI–MS *m/ z*: 566.45 [M + H]^+^.

#### (3-Chlorophenyl)-3-(5-((7-(pyridin-4-ylmethyl)-5,6,7,8-tetrahydropyrido[4',3':4,5]thieno[2,3-d]pyrimidin-4-yl)thio)-1,3,4-thiadiazol-2-yl)urea (11t)

C_24_H_19_ClN_8_OS_3_, Yellow solid, mp 122–124 °C. IR (KBr) (*ν*_max_/cm^–1^): 3358 and 2921 (2NH), 1636 (C=O), 1528, 1488, 1414, 1304, 1251, 1203, 1118, 1026, 824, 789, 699, 641. ^1^H NMR (300.1 MHz, DMSO-*d*_6_): *δ* 11.17 and 9.51 (2 s, 2H, 2NH), 8.72 (s, 1H, CH), 8.51 (d, *J* = 5.5 Hz, 2H, 2CH), 8.00 (s, 1H, CH), 7.62 (d, *J* = 8.2 Hz, 1H, CH), 7.38 (d, *J* = 5.5 Hz, 2H, 2CH), 7.27 (t, *J* = 8.0 Hz, 1H, CH), 6.97 (dd, *J* = 7.9, 1.2 Hz, 1H, CH), 3.78 and 3.76 (2 s, 4H, 2CH_2_N), 3.13 (t, *J* = 4.2 Hz, 2H, CH_2_C*H*_2_N), 2.87 (t, *J* = 4.8 Hz, 2H, C*H*_2_CH_2_N). ^13^C NMR (75.5 MHz, DMSO-*d*_6_): *δ* 165.9 (C = O); 165.4, 159.3, 156.0, 151.6, 149.7, 147.3, 145.6, 141.9, 135.5, 133.2, 130.3, 126.6, 125.2, 123.7, 121.3, 117.6, 116.7; 58.9, 53.4 and 47.9 (3CH_2_N); 25.9 (*C*H_2_CH_2_N).

#### (4-Chlorophenyl)-3-(5-((7-(pyridin-4-ylmethyl)-5,6,7,8-tetrahydropyrido[4',3':4,5]thieno[2,3-d]pyrimidin-4-yl)thio)-1,3,4-thiadiazol-2-yl)urea (11u)

C_24_H_19_ClN_8_OS_3_, White solid, mp 188–190 °C. IR (KBr) (*ν*_max_/cm^–1^): 3318 and 2923 (2NH), 1640 (C=O), 1578, 1530, 1493, 1427, 1316, 1247, 1204, 1123, 1030, 1005, 830, 743, 697, 620. ^1^H NMR (300.1 MHz, DMSO-*d*_6_):* δ* 10.96 and 9.53 (2 s, 2H, 2NH), 8.69 (s, 1H, CH), 8.51 (d, *J* = 4.8 Hz, 2H, 2CH), 7.80 (d, *J* = 8.6 Hz, 2H, 2CH), 7.38 (d, *J* = 4.8 Hz, 2H, 2CH), 7.27 (d, *J* = 4.8 Hz, 2H, 2CH), 3.78 and 3.76 (2 s, 4H, 2CH_2_N), 3.22 (t, *J* = 4.9 Hz, 2H, CH_2_C*H*_2_N), 2.87 (t, *J* = 4.9 Hz, 2H, C*H*_2_CH_2_N). ^13^C NMR (75.5 MHz, DMSO-*d*_6_): *δ* 165.8 (C = O); 165.4, 160.2, 157.7, 151.7, 149.7, 147.3, 143.6, 140.4, 135.3, 128.4, 126.6, 125.3, 124.5, 123.7, 119.4; 58.9, 53.4 and 47.9 (3CH_2_N); 23.3 (*C*H_2_CH_2_N).

#### (3,4-Dichlorophenyl)-3-(5-((7-(pyridin-4-ylmethyl)-5,6,7,8-tetrahydropyrido[4',3':4,5]thieno[2,3-d]pyrimidin-4-yl)thio)-1,3,4-thiadiazol-2-yl)urea (11v)

C_24_H_18_Cl_2_N_8_OS_3_,White solid, mp 252–255 °C. IR (KBr) (*ν*_max_/cm^–1^): 3318 and 2925 (2NH), 1636 (C=O), 1573, 1522, 1476, 1422, 1378, 1303, 1243, 1207, 1116, 1030, 866, 826, 796, 764, 677, 622. ^1^H NMR (300.1 MHz, DMSO-*d*_6_):* δ* 10.45 and 9.56 (2 s, 2H, 2NH), 8.69 (s, 1H, CH), 8.52 (d, *J* = 3.2 Hz, 2H, CH), 8.10 (d, *J* = 1.7 Hz, 1H, CH), 7.58 (dd, *J* = 8.7, 1.9 Hz, 1H, CH), 7.46–7.35 (m, 3H, 3CH), 3.80 and 3.78 (2 s, 4H, 2CH_2_N), 3.16 (t, *J* = 4.9 Hz, 2H, CH_2_C*H*_2_N), 2.89 (t, *J* = 4.9 Hz, 2H, C*H*_2_CH_2_N). ^13^C NMR (75.5 MHz, DMSO-*d*_6_): *δ* 165.7 (C = O); 165.4, 160.7, 151.8, 149.7, 147.3, 141.8, 136.2, 135.2, 130.8, 130.2, 126.6, 126.4, 125.4, 123.7, 122.4, 121.5, 117.7; 58.9, 53.4 and 47.9 (3CH_2_N); 23.3 (*C*H_2_CH_2_N).

#### (3-Chloro-4-methylphenyl)-3-(5-((7-(pyridin-4-ylmethyl)-5,6,7,8-tetrahydropyrido[4',3':4,5]thieno[2,3-d]pyrimidin-4-yl)thio)-1,3,4-thiadiazol-2-yl)urea (11w)

C_25_H_21_ClN_8_OS_3_, White solid, mp 157–159 °C. IR (KBr) (*ν*_max_/cm^–1^): 3246 and 2958 (2NH), 1637 (C=O), 1579, 1526, 1494, 1437, 1384, 1302, 1249, 1204, 1122, 1045, 1003, 960, 878, 823, 796, 745, 696, 634. ^1^H NMR (300.1 MHz, DMSO-*d*_6_): *δ* 11.18 and 9.52 (2 s, 2H, 2NH), 8.71 (s, 1H, CH), 8.50 (d, *J* = 5.0 Hz, 2H, 2CH), 8.00 (s, 1H, CH), 7.54 (d, *J* = 8.3 Hz, 1H, CH), 7.37 (d, *J* = 5.0 Hz, 2H, 2CH), 7.19 (d, *J* = 8.3 Hz, 1H, CH), 3.77 and 3.75 (2 s, 4H, 2CH_2_N), 3.15–3.07 (m, 2H, CH_2_C*H*_2_N), 2.95–2.75 (m, 2H, C*H*_2_CH_2_N), 2.22 (s, 3H, CH_3_). ^13^C NMR (75.5 MHz, DMSO-*d*_6_): *δ* 165.8 (C = O); 165.4, 159.5, 152.6, 151.7, 149.7, 147.3 (2C), 139.7, 135.4, 133.1, 131.1, 127.8, 126.6, 125.2, 123.7, 117.9, 116.8; 58.9, 53.4 and 47.9 (3CH_2_N); 25.9 (*C*H_2_CH_2_N); 18.9 (CH_3_).

#### (5-((7-(Pyridin-4-ylmethyl)-5,6,7,8-tetrahydropyrido[4',3':4,5]thieno[2,3-d]pyrimidin-4-yl)thio)-1,3,4-thiadiazol-2-yl)-3-(3-(trifluoromethyl)phenyl)urea (11x)

C_25_H_19_F_3_N_8_OS_3_, Yellow solid, mp 241–243 °C. IR (KBr) (*ν*_max_/cm^–1^): 3256 and 2923 (2NH), 1640 (C=O), 1549, 1494, 1423, 1383, 1310, 1252, 1213, 1161, 1113, 1029, 896, 832, 792, 744, 699, 654. ^1^H NMR (300.1 MHz, DMSO-*d*_6_):* δ* 11.46 and 9.51 (2 s, 2H, 2NH), 8.71 (s, 1H, CH), 8.50 (d, *J* = 5.0 Hz, 2H, 2CH), 8.35 (s, 1H, CH), 7.94 (d, *J* = 7.8 Hz, 1H, CH), 7.48 (t, *J* = 7.8 Hz, 1H, CH), 7.36 (d, *J* = 5.0 Hz, 2H, 2CH), 7.25 (d, *J* = 7.5 Hz, 1H, CH), 3.77 and 3.74 (2 s, 4H, 2CH_2_N), 3.16–3.06 (m, 2H, CH_2_C*H*_2_N), 2.92–2.78 (m, 2H, C*H*_2_CH_2_N). ^13^C NMR (75.5 MHz, DMSO-*d*_6_): *δ* 165.9 (C = O); 165.4, 159.3, 156.4, 151.6, 149.7, 147.3, 141.3, 135.4, 129.7, 129.3, 126.6, 126.2, 125.2, 123.7, 122.6, 121.7, 117.8; 59.0, 53.4 and 47.9 (3CH_2_N); 25.9 (*C*H_2_CH_2_N). ESI–MS *m/ z*: 600.19 [M + H]^+^.

#### (4-Chloro-3-(trifluoromethyl)phenyl)-3-(5-((7-(pyridin-4-ylmethyl)-5,6,7,8-tetrahydropyrido[4',3':4,5]thieno[2,3-d]pyrimidin-4-yl)thio)-1,3,4-thiadiazol-2-yl)urea (11y)

C_25_H_18_ClF_3_N_8_OS_3_, White solid, mp 257–260 °C. IR (KBr) (*ν*_max_/cm^–1^): 3286 and 2924 (2NH), 1634 (C=O), 1529, 1490, 1412, 1312, 1267, 1243, 1176, 1113, 1030, 904, 830, 744, 661, 630. ^1^H NMR (300.1 MHz, DMSO-*d*_6_): *δ* 11.61 (s, 1H, NH), 8.65 (s, 1H, CH), 8.49 (d, *J* = 4.8 Hz, 2H, 2CH), 8.37 (s, 1H, CH), 7.90 (d, *J* = 8.6 Hz, 1H, CH), 7.46 (d, *J* = 8.6 Hz, 1H, CH), 7.34 (d, *J* = 4.8 Hz, 2H, 2CH), 4.39 (s, 1H, NH), 3.72 (br. s, 4H, 2CH_2_N), 3.12–3.00 (m, 2H, CH_2_C*H*_2_N), 2.82 (t, *J* = 4.2 Hz, 2H, C*H*_2_CH_2_N). ^13^C NMR (75.5 MHz, DMSO-*d*_6_): *δ* 165.7 (C = O); 159.3, 156.0, 151.5, 149.6, 147.2, 145.3, 140.2, 135.3, 131.6, 126.8, 126.4, 125.1, 124.8, 123.6, 122.5, 121.6, 121.2, 116.3; 59.0, 51.4 and 48.9 (3CH_2_N); 25.5 (*C*H_2_CH_2_N).

### Ethical approval and statement

All methods were carried out in accordance with relevant guidelines and regulations of The Ministry of Health and Medical Education IR.TUMS.VCR.REC.1397.140. Protocols were conducted and approved by Motamed Cancer Institute . All methods are reported in accordance with ARRIVE guidelines.

### MTT assay

Cytotoxicity of the compounds was measured by detecting the ability of cells in transforming MTT into a purple formazan dye. The different cell lines were provided by the National Cell Bank of Iran (Pastor Institute, Tehran, Iran). The required materials and reagents were purchased from Sigma. After the cell culture in RPMI-1640 medium and DMEM containing 10% FBS (Gibco, Milano, Italy), the suspension of MCF-7 (7000) and other cells (5000) were poured into 96 well plates and incubated in a humidified incubator containing 5% CO_2_ at 37 °C for 24 h. Subsequently, a solution of the compounds in DMSO was added to these plates and incubated for 48 h. After incubation, the solution of 5% 3-(4,5-Dimethyl-2-thiazolyl)-2,5-diphenyl-2*H*-tetrazolium bromide (MTT) was added to all the wells and incubation was continued for another 4 h. The record of color intensity of the formazan solution was at 570 nm by Bio-Rad microplate reader (Model 680) which reflects the cell growth condition^26^.

### Apoptosis inducing analysis

For determination of apoptosis induction, the best concentration (IC_50_) of the most active compounds were selected and evaluated on HUVEC, MCF-7 cell lines. After incubation of in a 6-well plate at 37 °C for 24 h, they were treated with selected compounds at their IC_50_ concentration for 48 h. Then, cells were trypsinized, rinsed with phosphate-bufferede buffered saline (PBS) and then centrifuged at 1200 rpm for 3 min. After this, the binding buffer (500 μL) was added to the resulting cells, followed by addition and mixing with Annexin V-APC and PI (5 μL). After this, the samples were incubated in dark for 10 to 15 min at room temperature, and then the cellular analysis was measured by flow cytometer (FACS Calibur Bectone-Dickinson).

### Caspase-3 assay

Caspase-3 activity was measured using a caspase-3 (Active) (human) ELISA kit, MBS450774, according to the manufacturer’s instructions.

### Cell-cycle analysis

The selected compounds were examined on HUVEC, MCF-7 cells at IC_50_ concentration for 48 h. After trypsinization and rinsing with PBS, the cells were centrifuged at 1200 rpm for 5 min and then incubated with PBS and fixed in ice-cooled 70% ethanol. After washing with PBS, the cells were resuspended in RNase A (0.1 mg/mL) and incubated for 5 h. Then, staining with PI (50 mg/mL) and incubation for 15 min were performed. By using Novocyte flow cytometer (ACEA Biosciences) the analysis was done and the calculation of cell cycle distributions was done by NovoExpress 1.1.0 software.

### Chick chorioallantoic membrane (CAM) assay

All eggs were supplied from (Poultry Breeding Farm, Tehran, Iran). Since the allantois of the chick embryo appears at about 3.5 days of incubation, the corresponding eggs were incubated (37.5 °C, relative humidity: 55–65%). To decrease the risk of infection, the particular regions of the egg, *e.g.*, the front side of the embryo were cleaned and sterilized with 70% EtOH. On the 6th incubation day, a false air sac was created directly over the CAM, permitting its detachment from the shell membrane. The procedure was continued through a square incision over the CAM. The prepared window in a square form (1 × 1 cm), was covered by a flexible film and then transported to the incubator. On the 8th incubation day, the particular paper discs (Whatman) were coated with target agents and placed in the central area of the corresponding window. The surface of CAM was visually evaluated on the 12th incubation day using the stereomicroscope. We decided to use the number and the length of capillary blood vessels as the index of anti-angiogenic activity. Consequently, the prepared images were analyzed by the advantageous software, Image J^22,23^.

### Western blot analysis

HUVEC cells were treated with Sorafenib and the most active compound at IC_50_ concentration and incubated for 24, 48, and 72 h. After thawing in 100 mL lysis buffer and centrifuge at 1200 rpm, the loading and electrophoresis of proteins on 12% SDSPAGE was performed. Then, they transferred for 1.5 h at 100 V into a nitrocellulose membrane (0.45 mm) and blocked with 5% blocking buffer. After incubation of antibodies at 4 ◦C for 12 h, the detection of immunoblots were performed using the enhanced chemiluminescence (ECL) method.

### Docking studies

To investigate the interactions of compound **11n**, the AutoDock 4.2 program was used. The crystallographic structure of the VEGFR-2 in complex with ligand sorafenib (3WZE) was taken from PDB. At first, the 2D structures were prepared by using Marvin Sketch 15.10.12 and converted to pdb format. A series of changes using AutoDockTools version 1.5.6 were applied. To create the corresponding pdbqt file on the receptor, including the addition of polar hydrogens, assignment of partial atomic charges, and set up of rotatable bonds. A box of 81 (x) × 61 (y) × 64 (z) grid points spaced 0.375 Å apart was selected to ensure sufficient volume for accommodation of the VEGFR-2- **11n** system. The Lamarckian genetic algorithm with an initial population size of 50 runs was used for generating conformations of ligand (**11n**) docked to receptor. To visualize interactions, Discovery Studio Visualizer (Ver. 17.2) and Pymol (Ver. 1.level) was used. The docking protocol was validated by calculating the RMSD^37,38^.

## Supplementary Information

Supplementary Information.

## Data Availability

All data generated or analysed during this study are included in this published article and its supplementary information files.
