# Supplementary material for: Design, synthesis and evaluation of novel tetrahydropyridothienopyrimidin-ureas as cytotoxic and anti-angiogenic agents
Source: Sci Rep. 2022 Jun 11;12:9683. doi: 10.1038/s41598-022-13515-4 (PMC9188586; doi:10.1038/s41598-022-13515-4)
Supplement: Supplementary file 1 — Supplementary Information. [file 41598_2022_13515_MOESM1_ESM.docx]

**Design and synthesis of novel tetrahydropyridothienopyrimidin-ureas: cytotoxic and anti-angiogenesis properties**

Rasoul Motahari,^a^ Mohammad Amin Boshagh,^b^ Setareh Moghimi,^c^ Fariba Peytam,^c^ Zaman Hasanvand,^a^ Tayebeh Oghabi Bakhshaiesh ,^b^ Roham Foroumadi,^d^ Hamidreza Bijanzadeh,^e^ Loghman Firoozpour,^a^ Ali Khalaj,^a^ Rezvan Esmaeili,^b,*^, Alireza Foroumadi^a,c**^

^a^ *Department of Medicinal Chemistry, Faculty of Pharmacy, Tehran University of Medical Sciences, Tehran, Iran*

*^b^Genetics Department, Breast Cancer Research Center, Motamed Cancer Institute, ACECR,*

*Tehran, Iran*

*^c^Drug Design and Development Research Center, The Institute of Pharmaceutical Sciences (TIPS), Tehran University of Medical Sciences, Tehran, Iran*

*^d^Department of Pharmacology, School of Medicine, Tehran University of Medical Sciences,*

*Tehran, Iran*

*^e^Department of Environmental Sciences, Faculty of Natural Resources and Marine Sciences, Tarbiat Modares University, Tehran, Iran*

Corresponding authors:

* E-mail: [esmaeili.rezvan@gmail.com](mailto:esmaeili.rezvan@gmail.com) (R. Esmaeili); Tel.: 98 21 88876869; Fax: 98 88796208.

** E-mail: aforoumadi@yahoo.com (A. Foroumadi); Tel.: 98 21 66406757; Fax: 98 21 66461178.


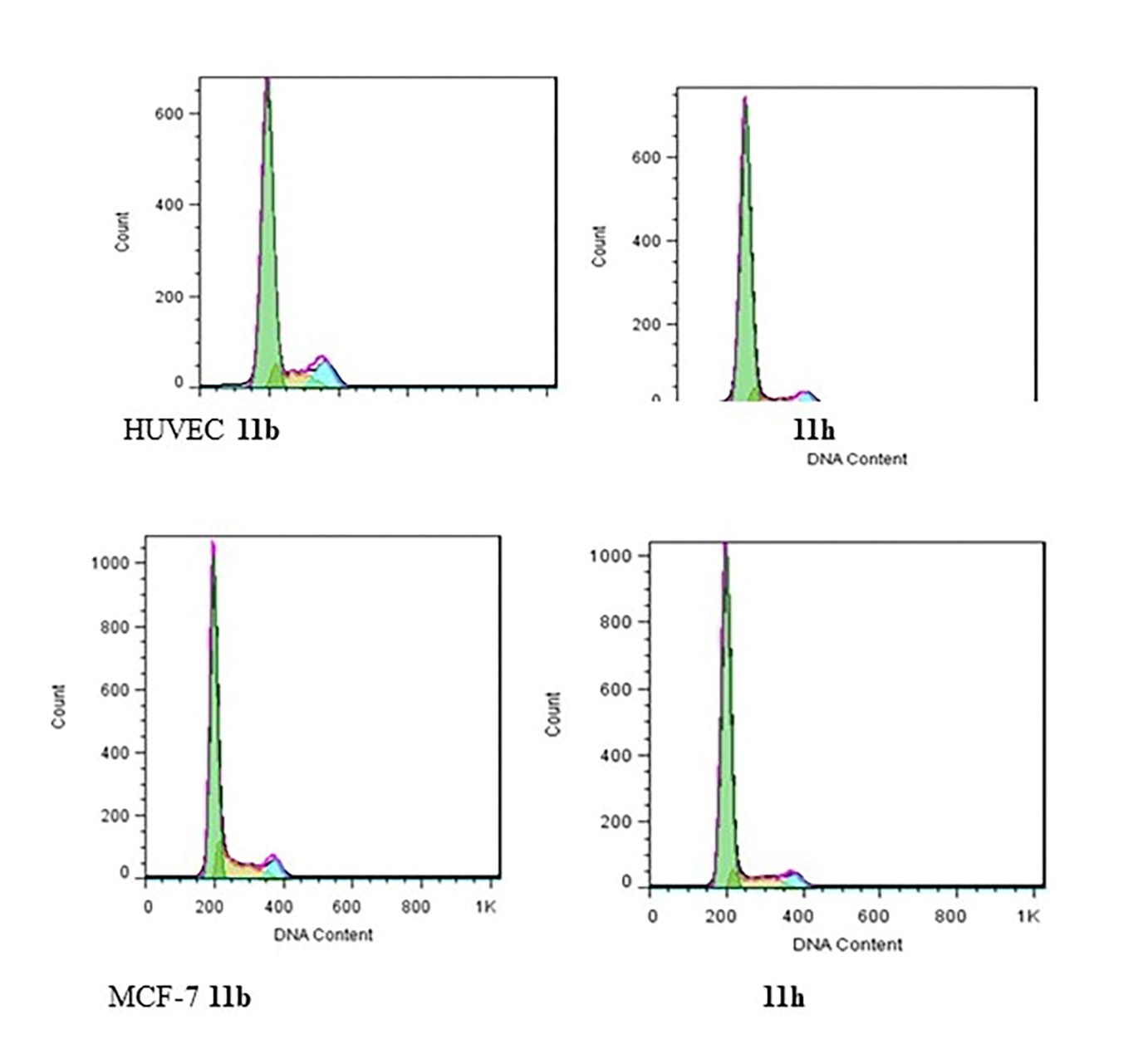


Fig S1. Cell cycle analysis of compounds **11b, 11h**


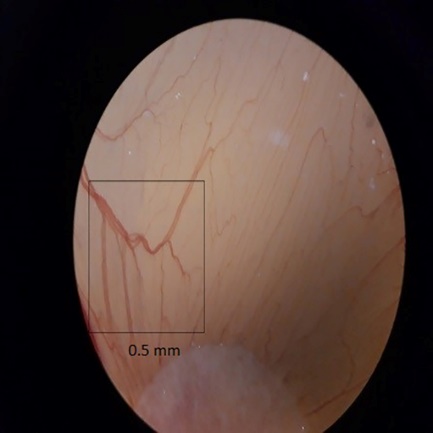

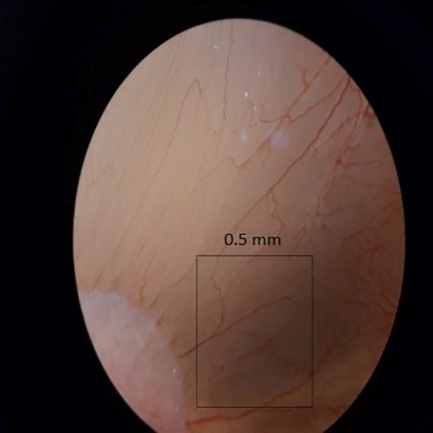


**11b**

**11h**


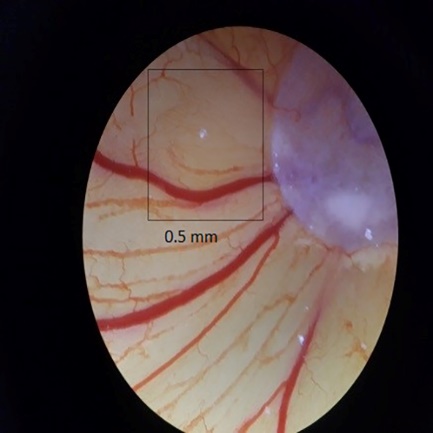

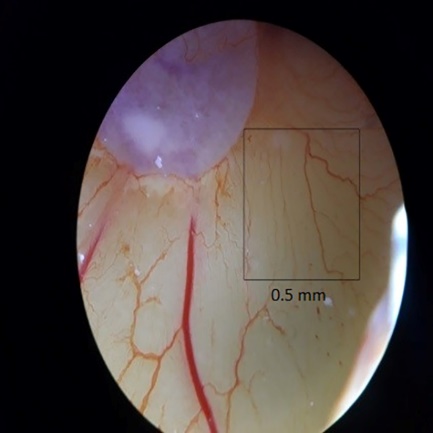


Fig S2. CAM assay of compounds 11b, 11h

As attached figures are shown, samples were run on a separate PVDF for detecting each antibody.


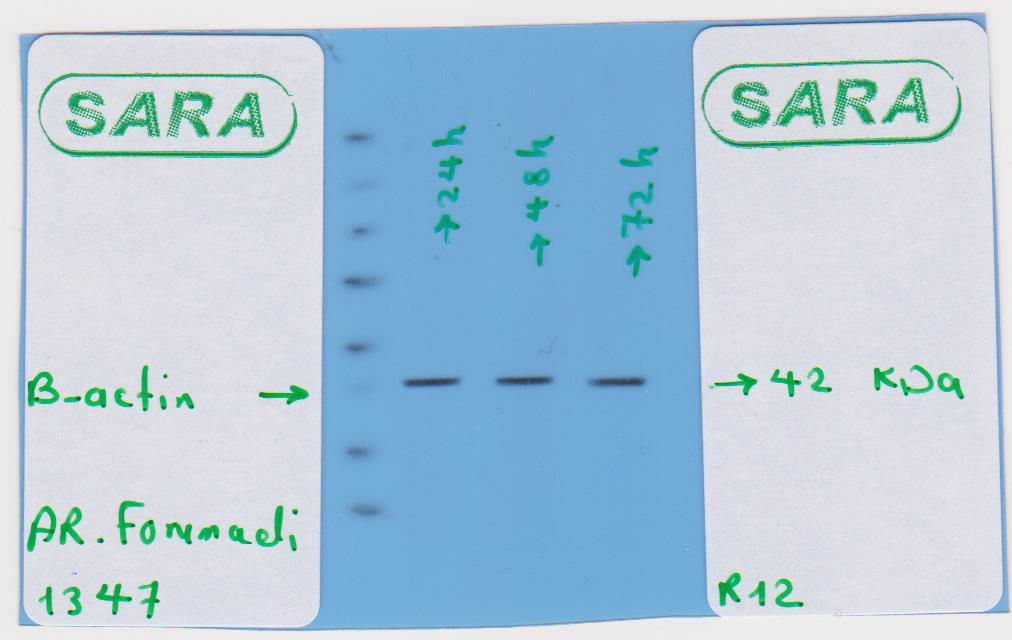


Figure S3. Beta-Actin for sample (11n=R12)


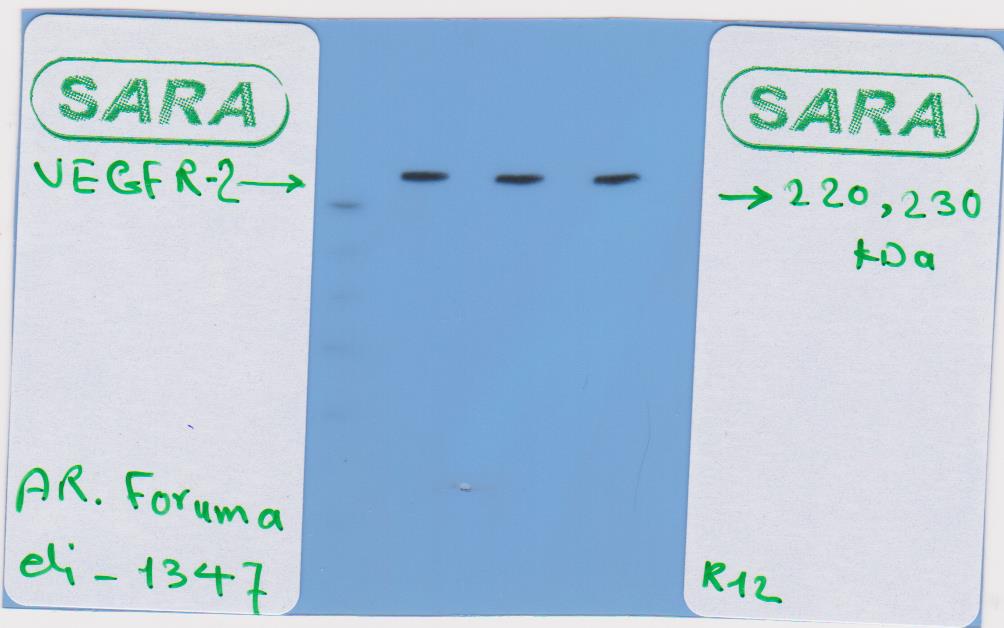


Figure S4. VEGFR2 for sample (11n=R12)


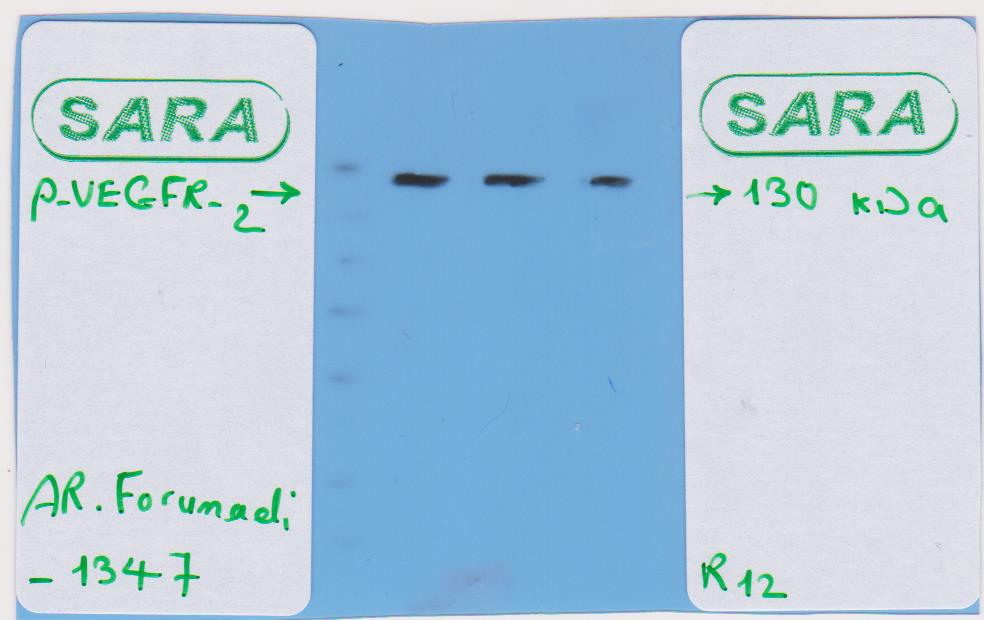


Figure S5. P-VEGFR2 for sample (11n=R12)


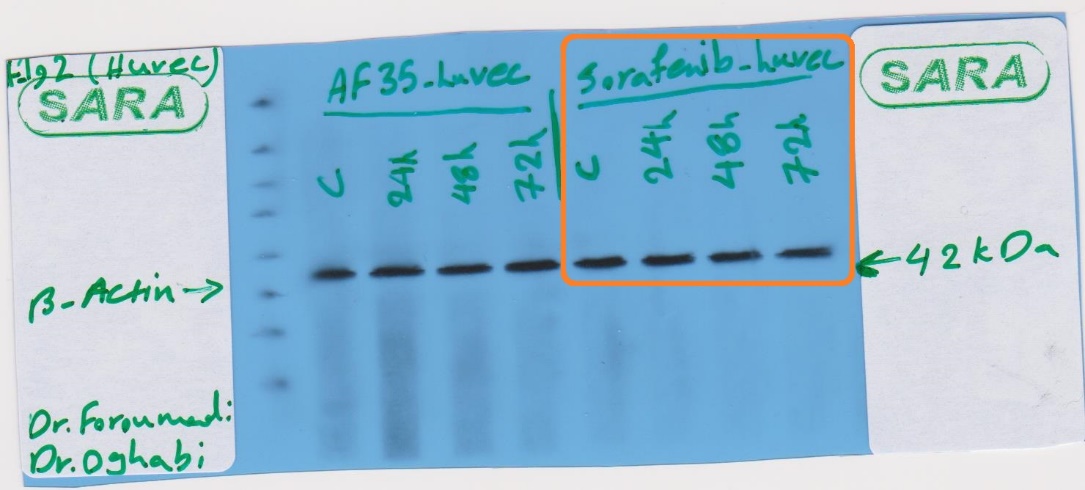


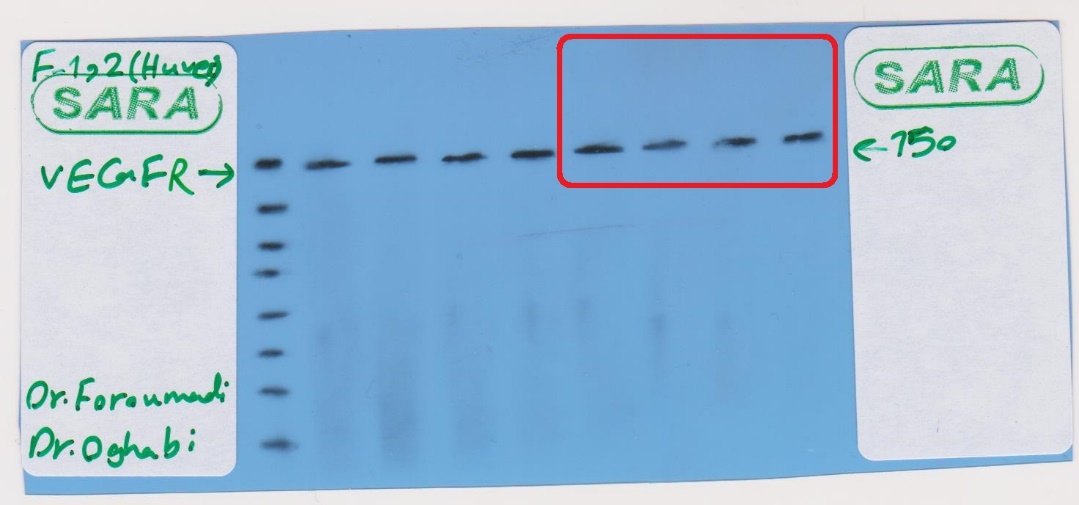


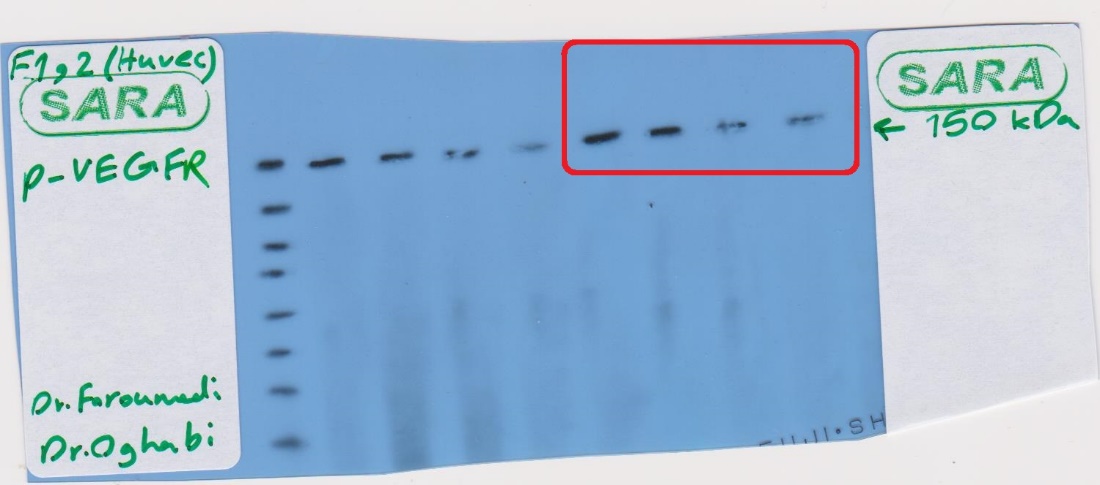


Figure S6. Beta-Actin, VEGFR2 and P-VEGFR2 for sorafenib. The part without red box is not belongs to this study. But, based on image policy we did not crop the image.


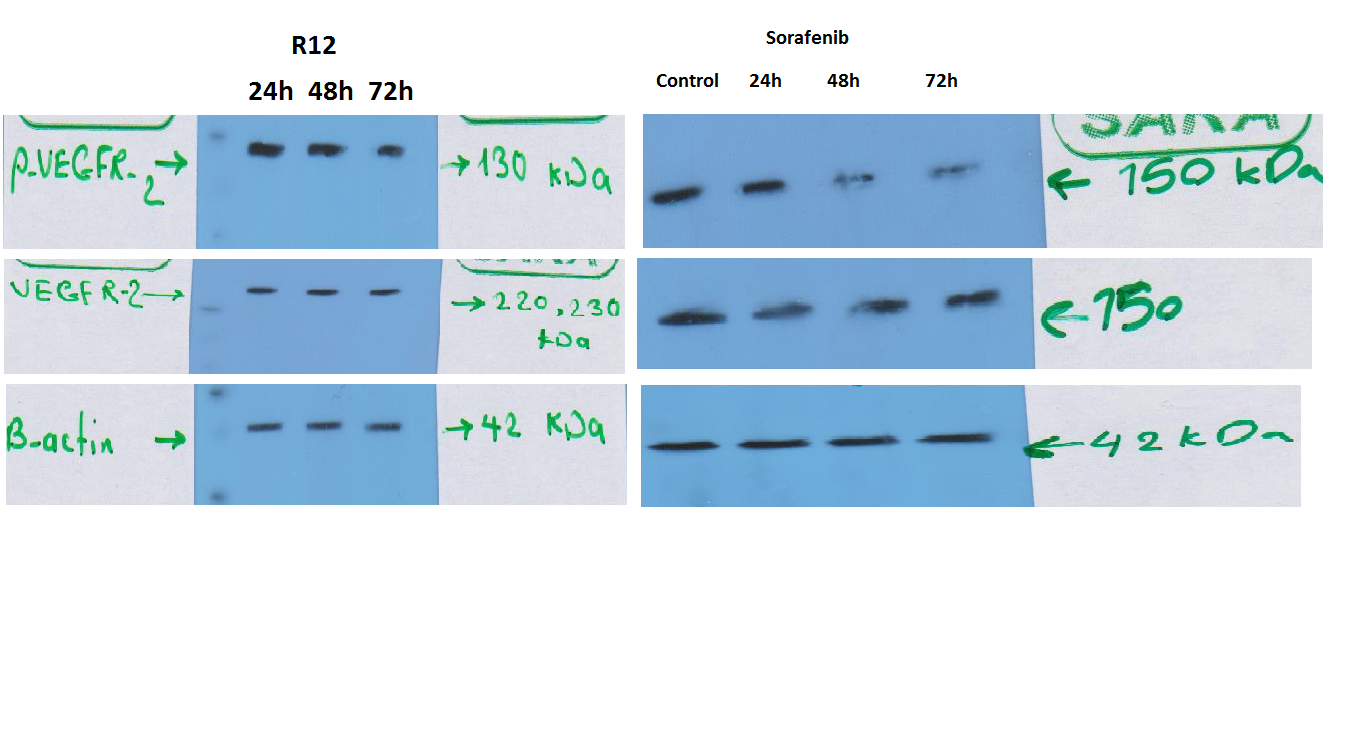


Figure S7. The cropped picture of western blot analysis

^1^H NMR spectrum of 1-(5-((7-benzyl-5,6,7,8-tetrahydropyrido[4',3':4,5]thieno[2,3-d]pyrimidin-4-yl)thio)-1,3,4-thiadiazol-2-yl)-3-phenylurea (**11a**)
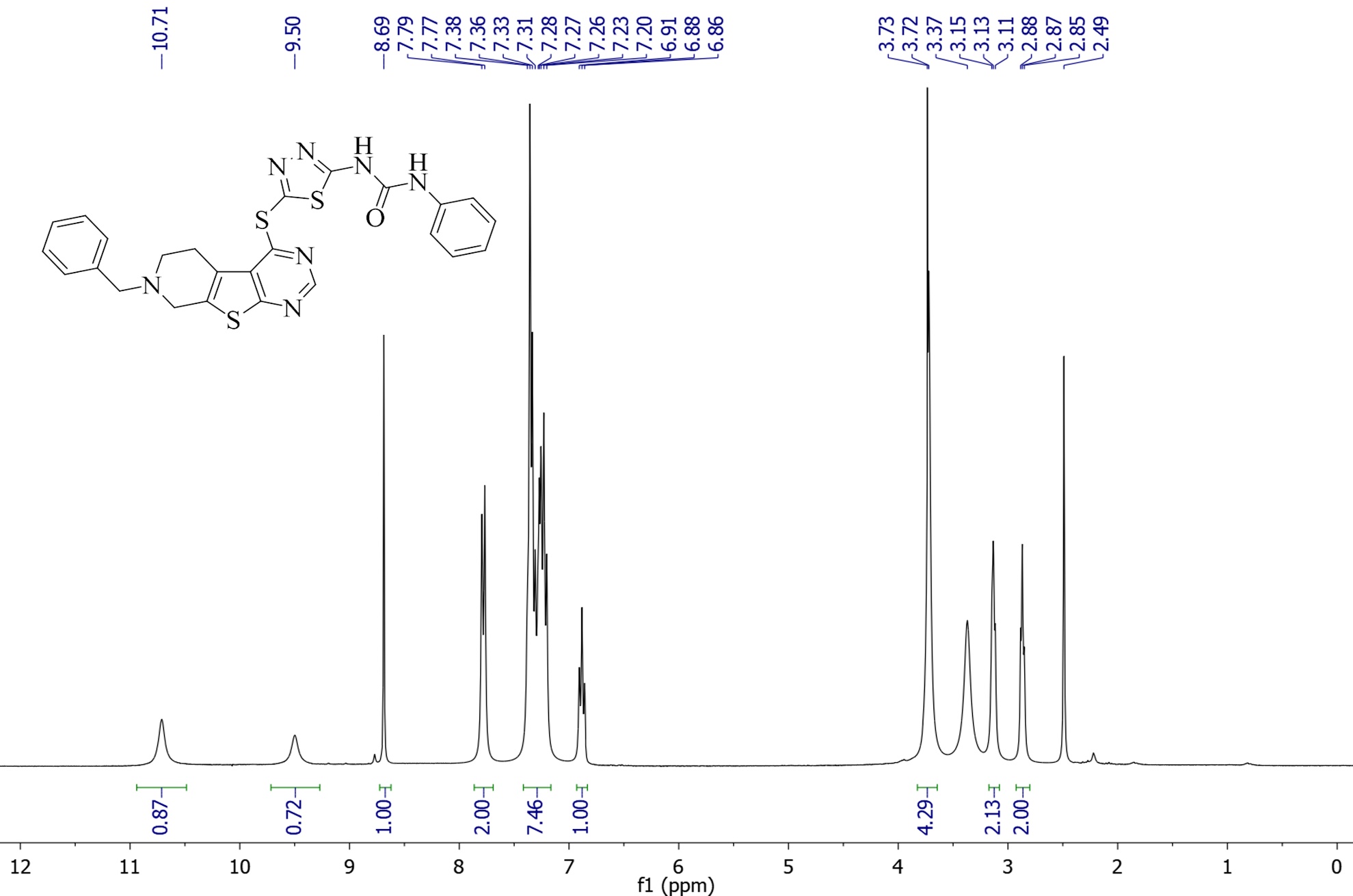


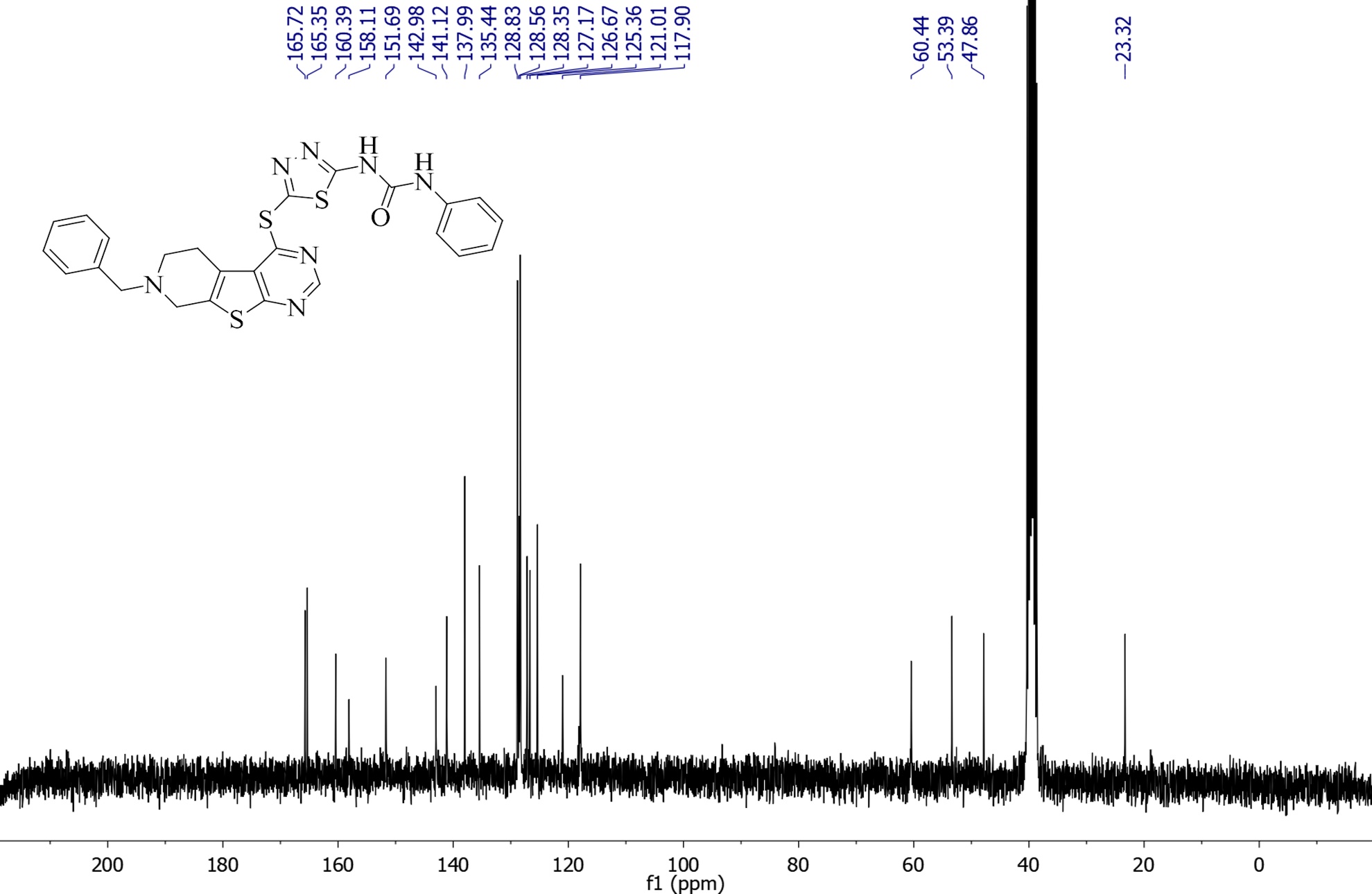
^13^C NMR spectrum of 1-(5-((7-benzyl-5,6,7,8-tetrahydropyrido[4',3':4,5]thieno[2,3-d]pyrimidin-4-yl)thio)-1,3,4-thiadiazol-2-yl)-3-phenylurea (**11a**)


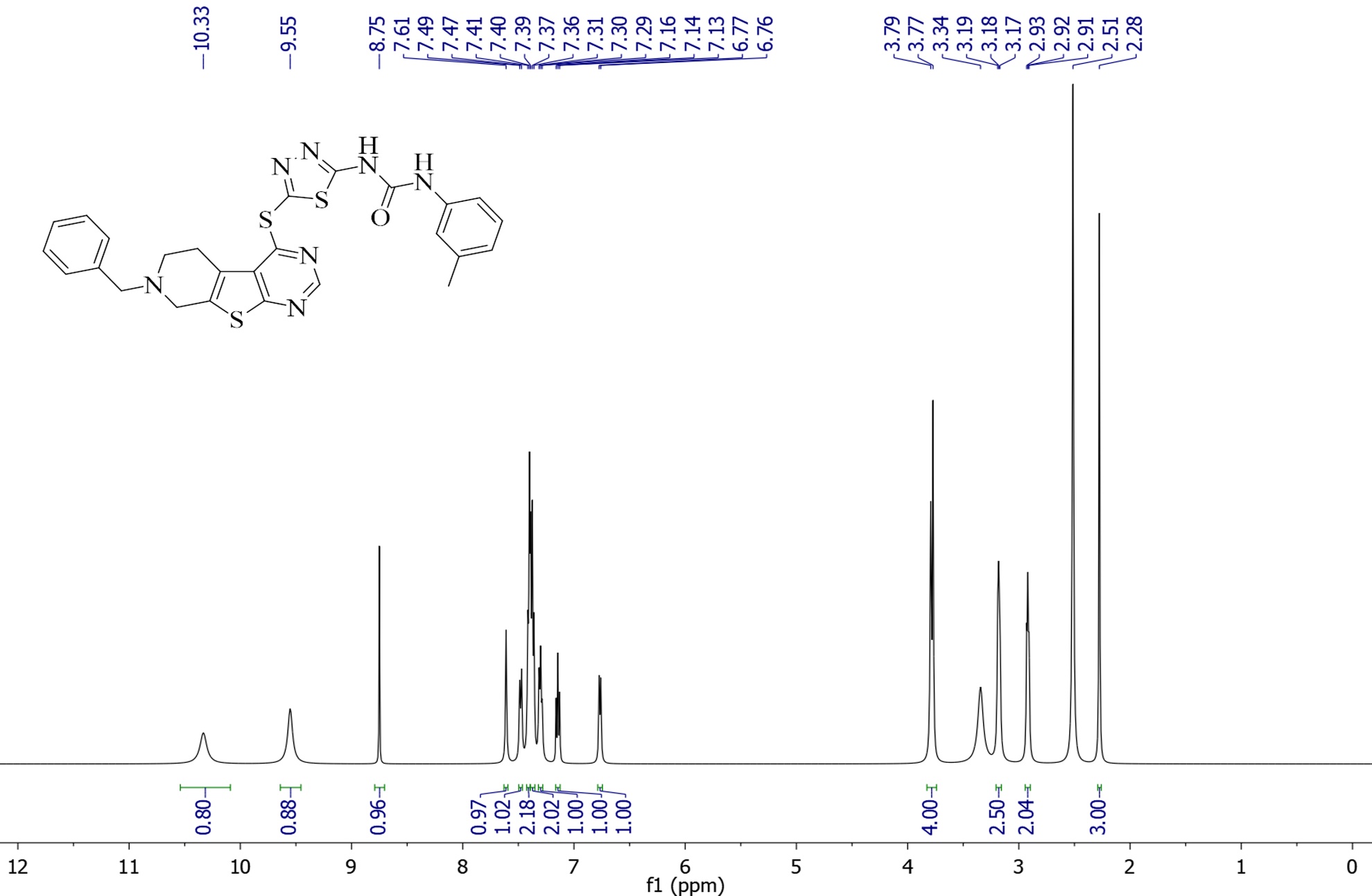
^1^H NMR spectrum of 1-(5-((7-benzyl-5,6,7,8-tetrahydropyrido[4',3':4,5]thieno[2,3-d]pyrimidin-4-yl)thio)-1,3,4-thiadiazol-2-yl)-3-(m-tolyl)urea (**11b**)


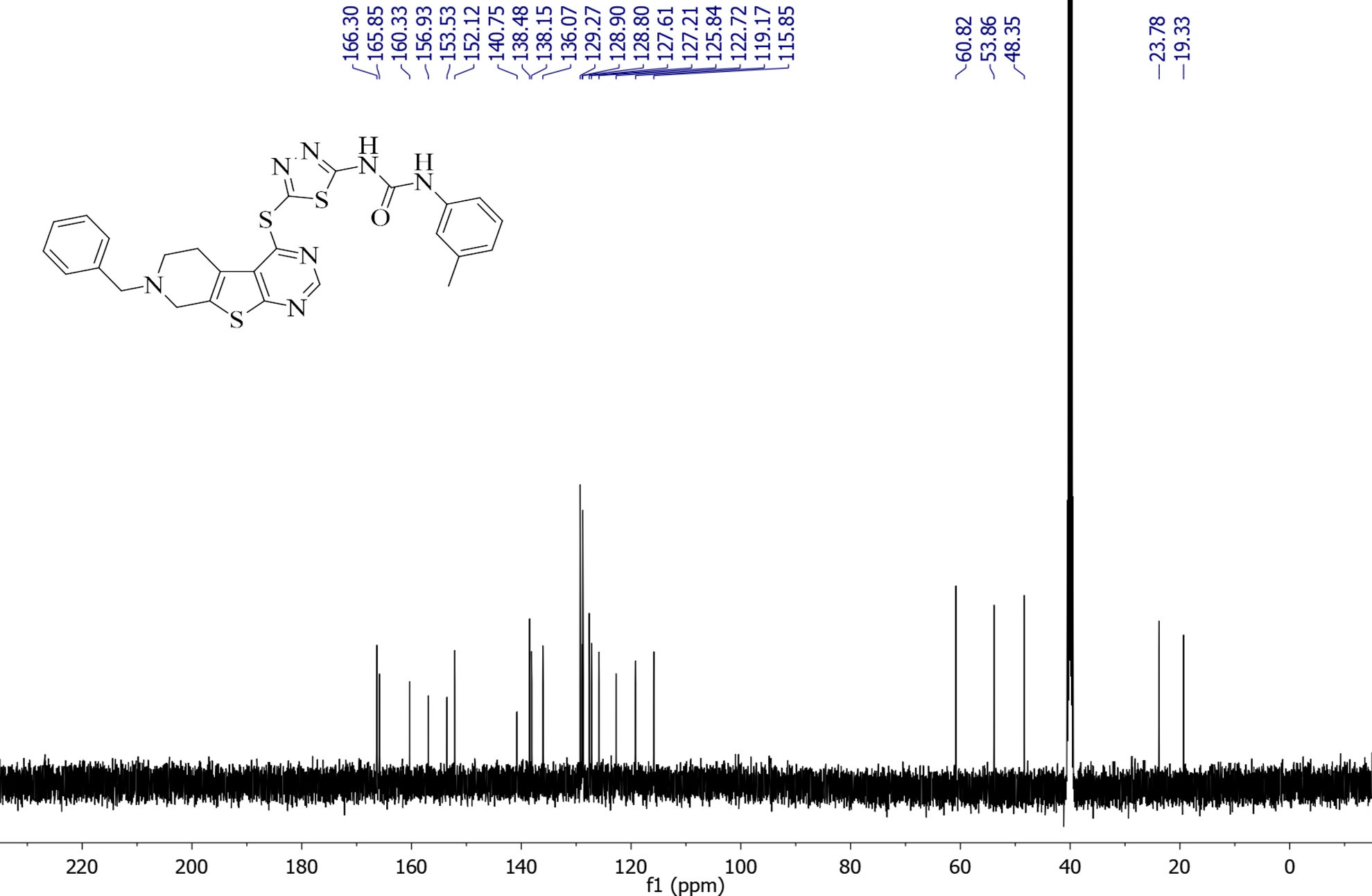
^13^C NMR spectrum of 1-(5-((7-benzyl-5,6,7,8-tetrahydropyrido[4',3':4,5]thieno[2,3-d]pyrimidin-4-yl)thio)-1,3,4-thiadiazol-2-yl)-3-(m-tolyl)urea (**11b**)


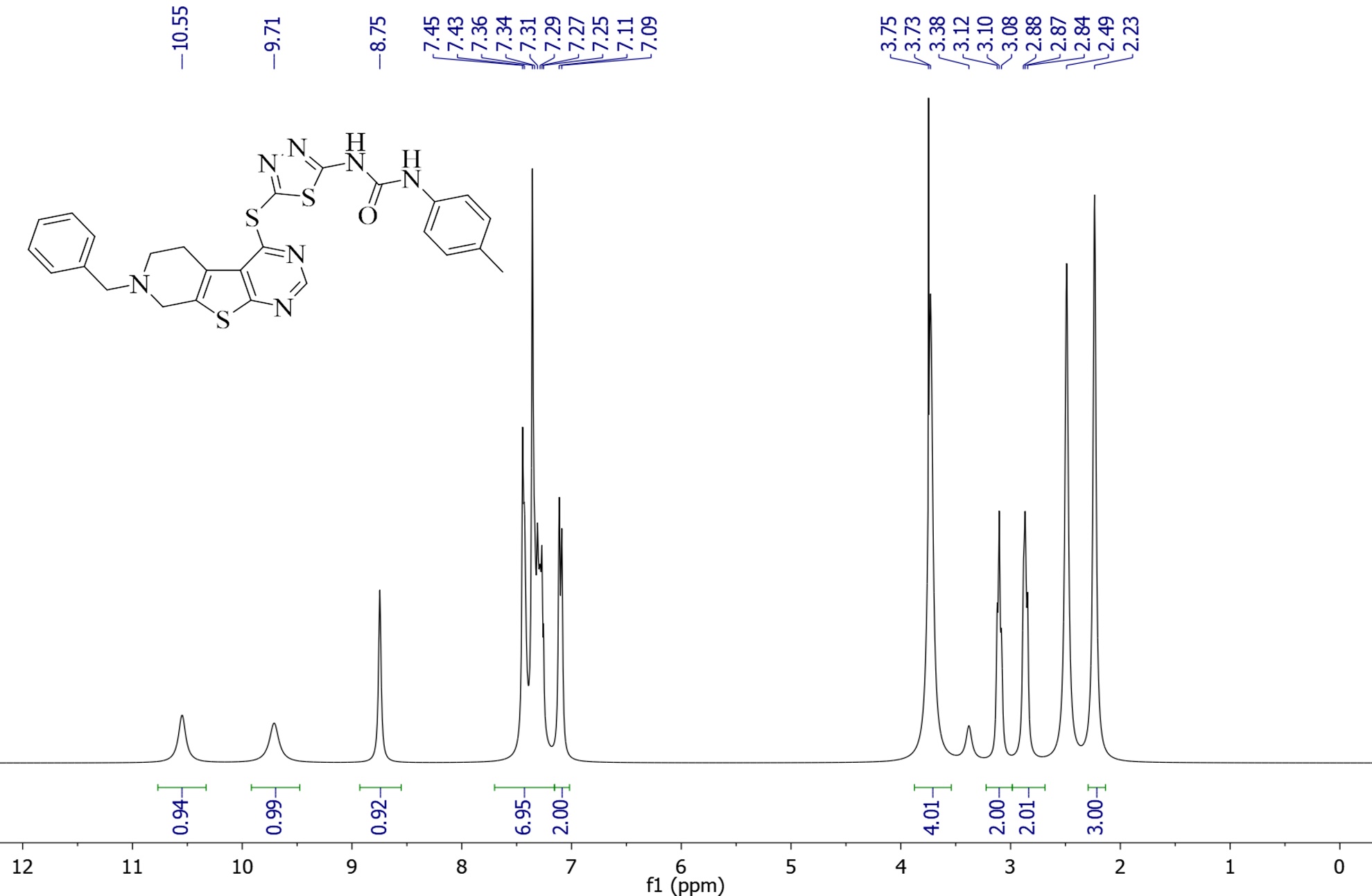
^1^H NMR spectrum of 1-(5-((7-benzyl-5,6,7,8-tetrahydropyrido[4',3':4,5]thieno[2,3-d]pyrimidin-4-yl)thio)-1,3,4-thiadiazol-2-yl)-3-(p-tolyl)urea (**11c**)


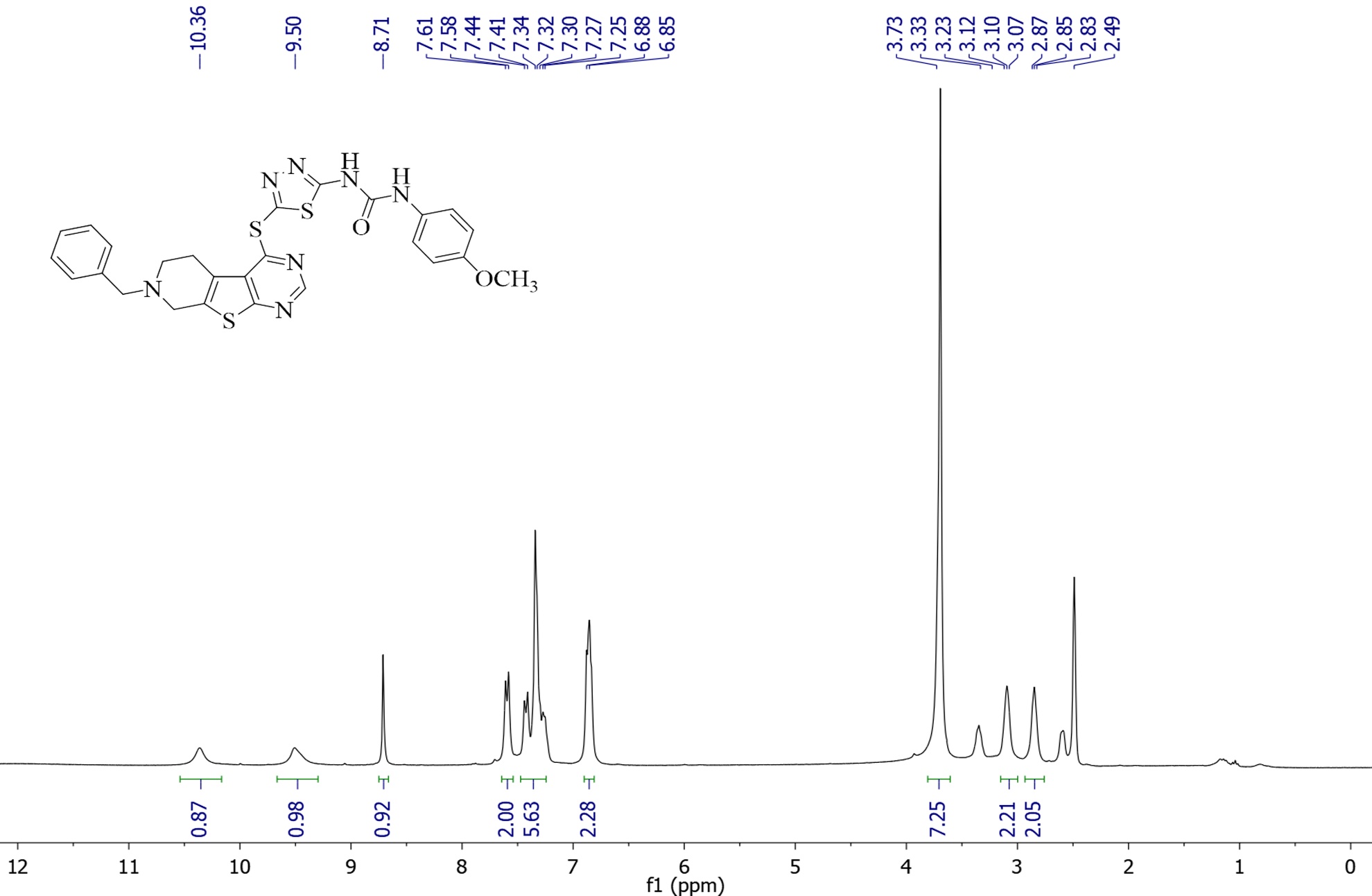
^1^H NMR spectrum of 1-(5-((7-benzyl-5,6,7,8-tetrahydropyrido[4',3':4,5]thieno[2,3-d]pyrimidin-4-yl)thio)-1,3,4-thiadiazol-2-yl)-3-(4-methoxyphenyl)urea (**11d**)

^
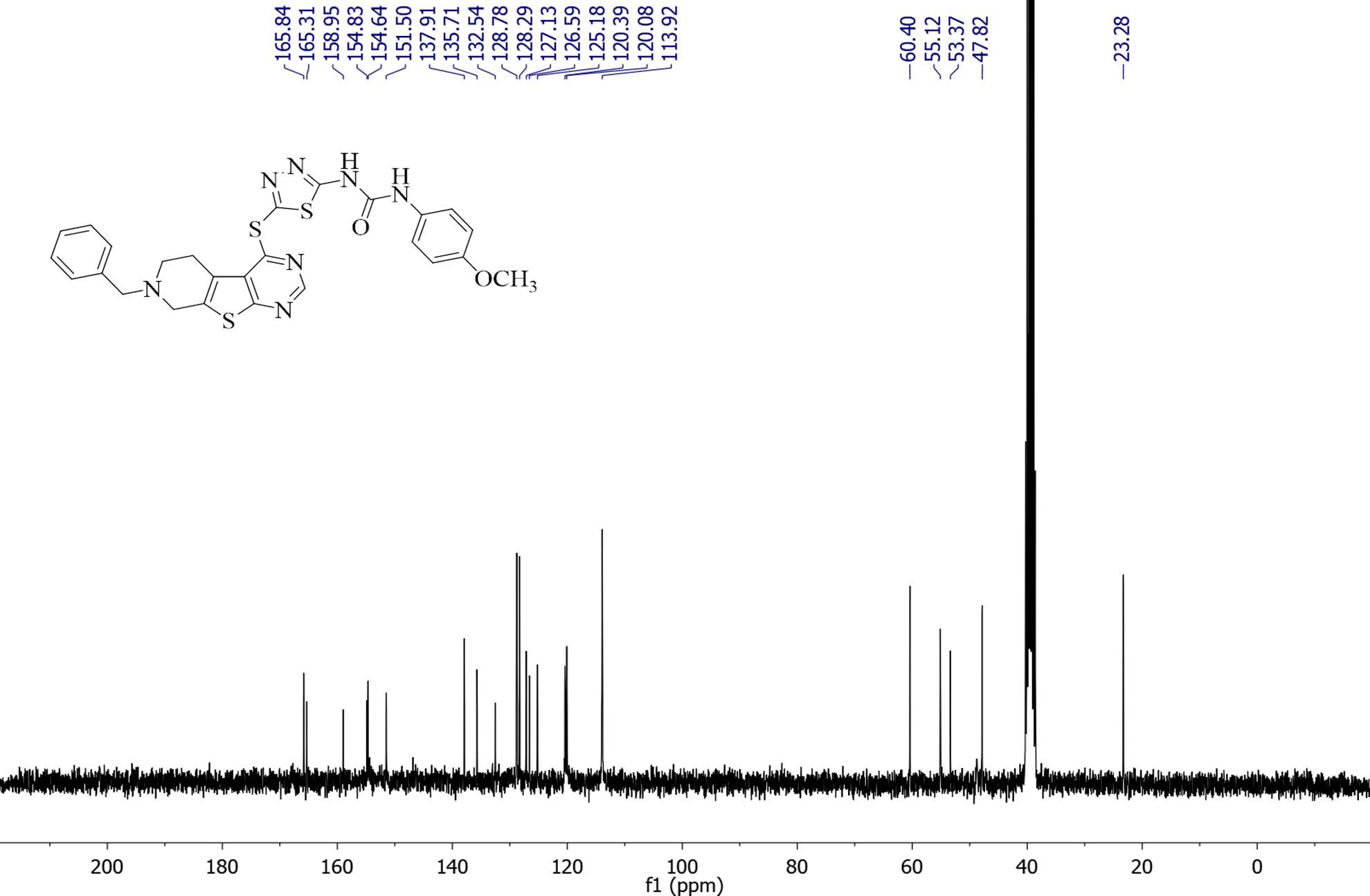
13^C NMR spectrum of 1-(5-((7-benzyl-5,6,7,8-tetrahydropyrido[4',3':4,5]thieno[2,3-d]pyrimidin-4-yl)thio)-1,3,4-thiadiazol-2-yl)-3-(4-methoxyphenyl)urea (**11d**)


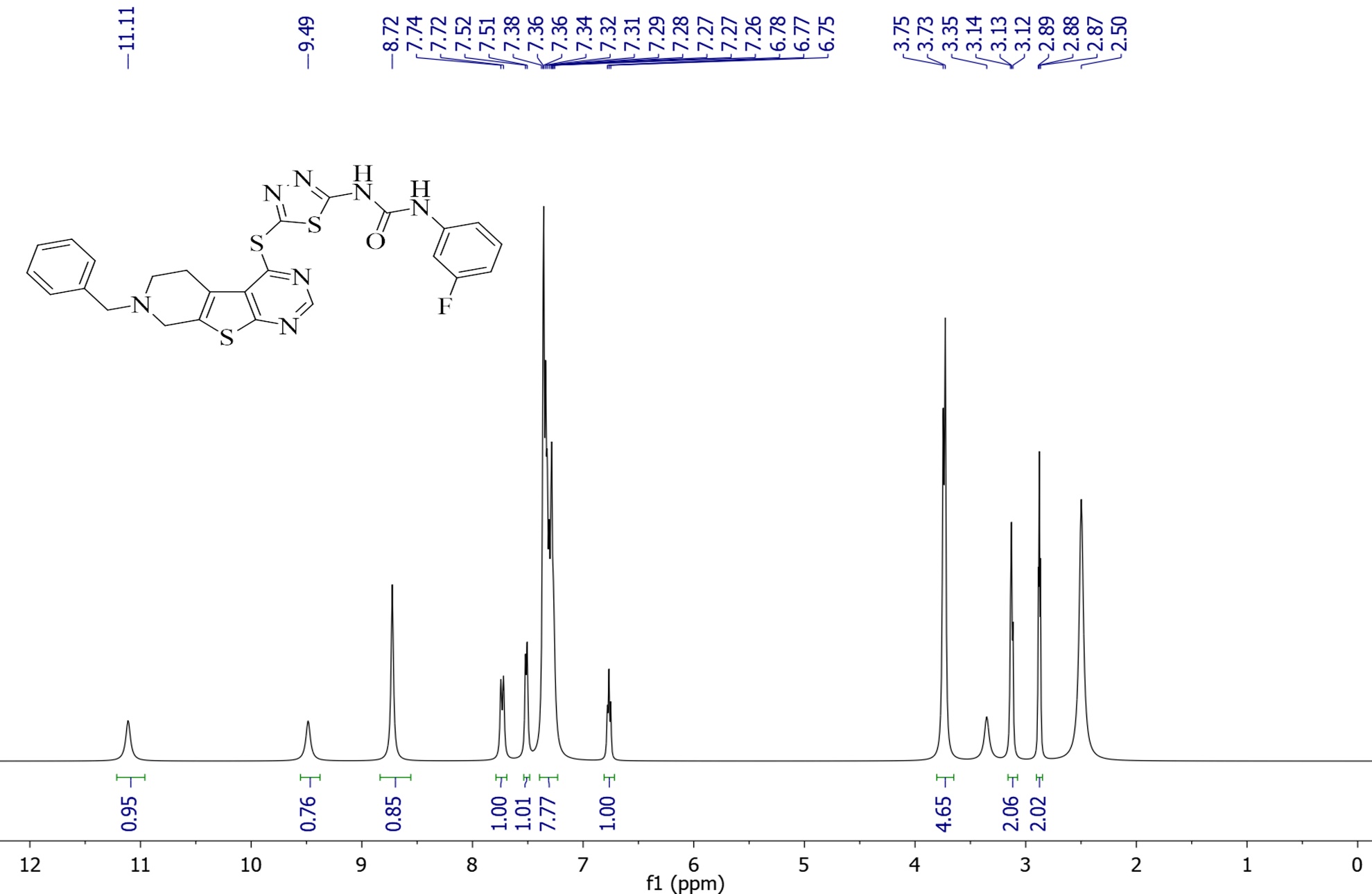
^1^H NMR spectrum of 1-(5-((7-benzyl-5,6,7,8-tetrahydropyrido[4',3':4,5]thieno[2,3-d]pyrimidin-4-yl)thio)-1,3,4-thiadiazol-2-yl)-3-(3-fluorophenyl)urea (**11e**)


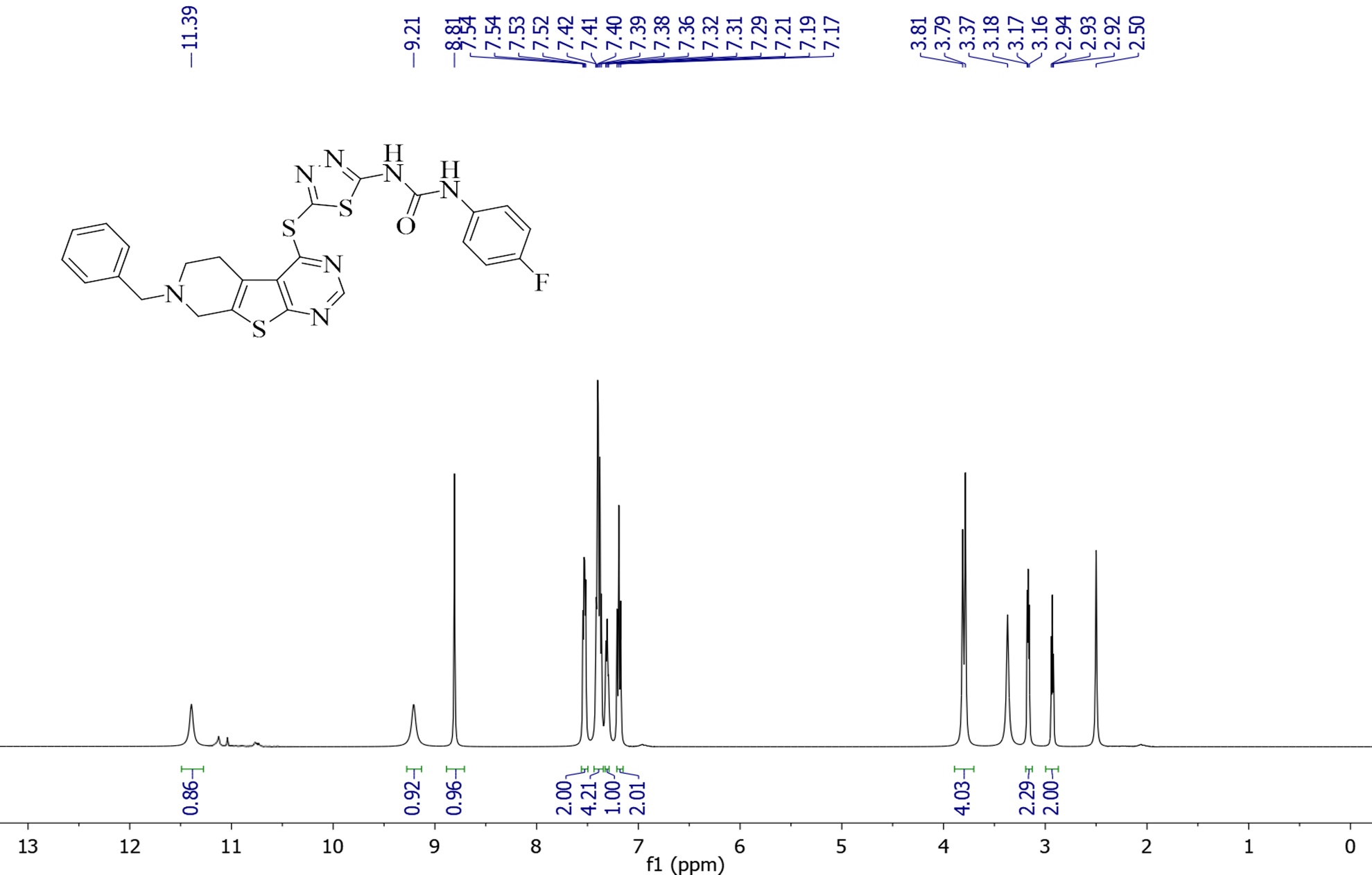
^1^H NMR spectrum of 1-(5-((7-benzyl-5,6,7,8-tetrahydropyrido[4',3':4,5]thieno[2,3-d]pyrimidin-4-yl)thio)-1,3,4-thiadiazol-2-yl)-3-(4-fluorophenyl)urea (**11f**)


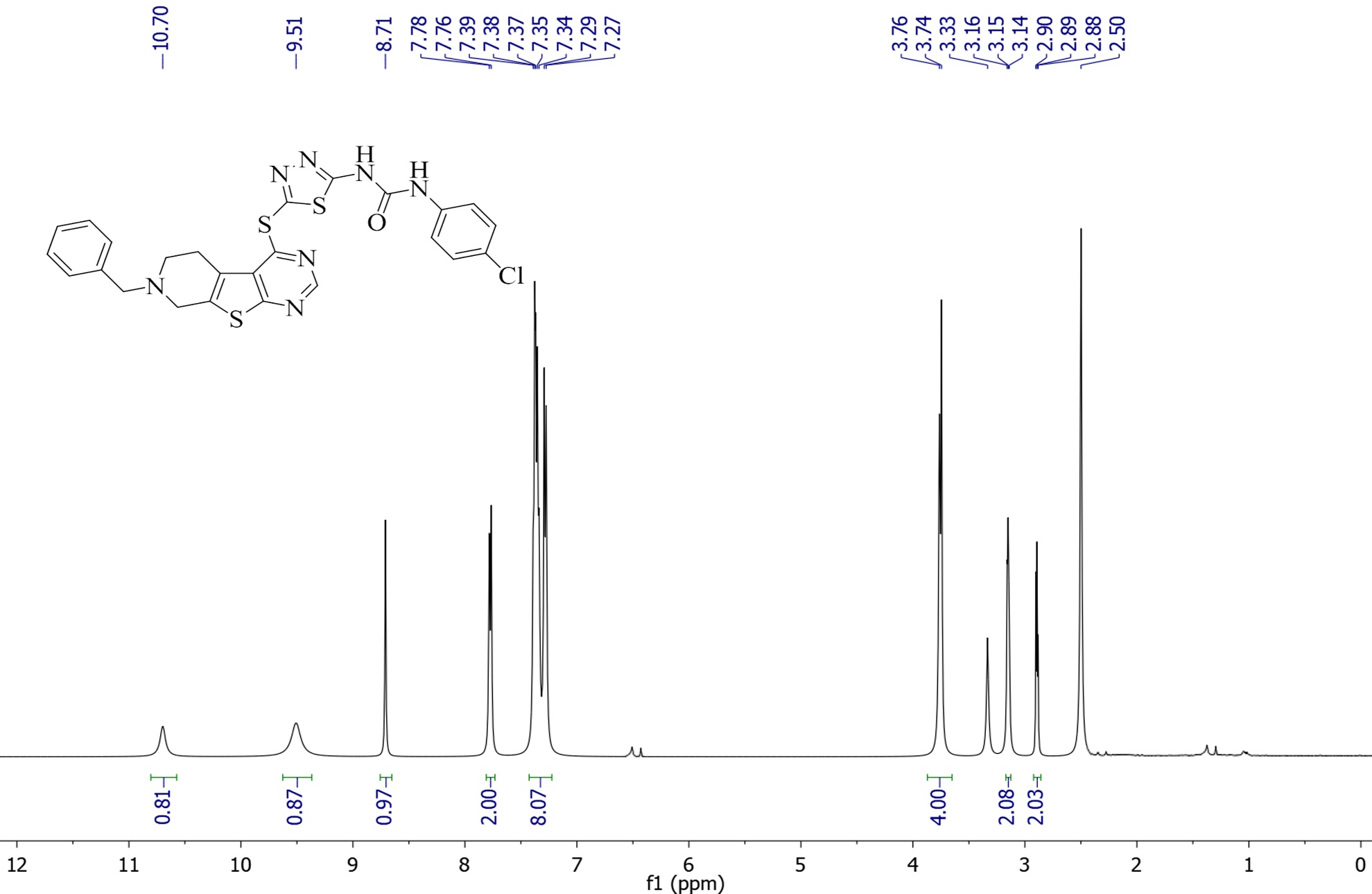
^1^H NMR spectrum of 1-(5-((7-benzyl-5,6,7,8-tetrahydropyrido[4',3':4,5]thieno[2,3-d]pyrimidin-4-yl)thio)-1,3,4-thiadiazol-2-yl)-3-(4-chlorophenyl)urea (**11g**)


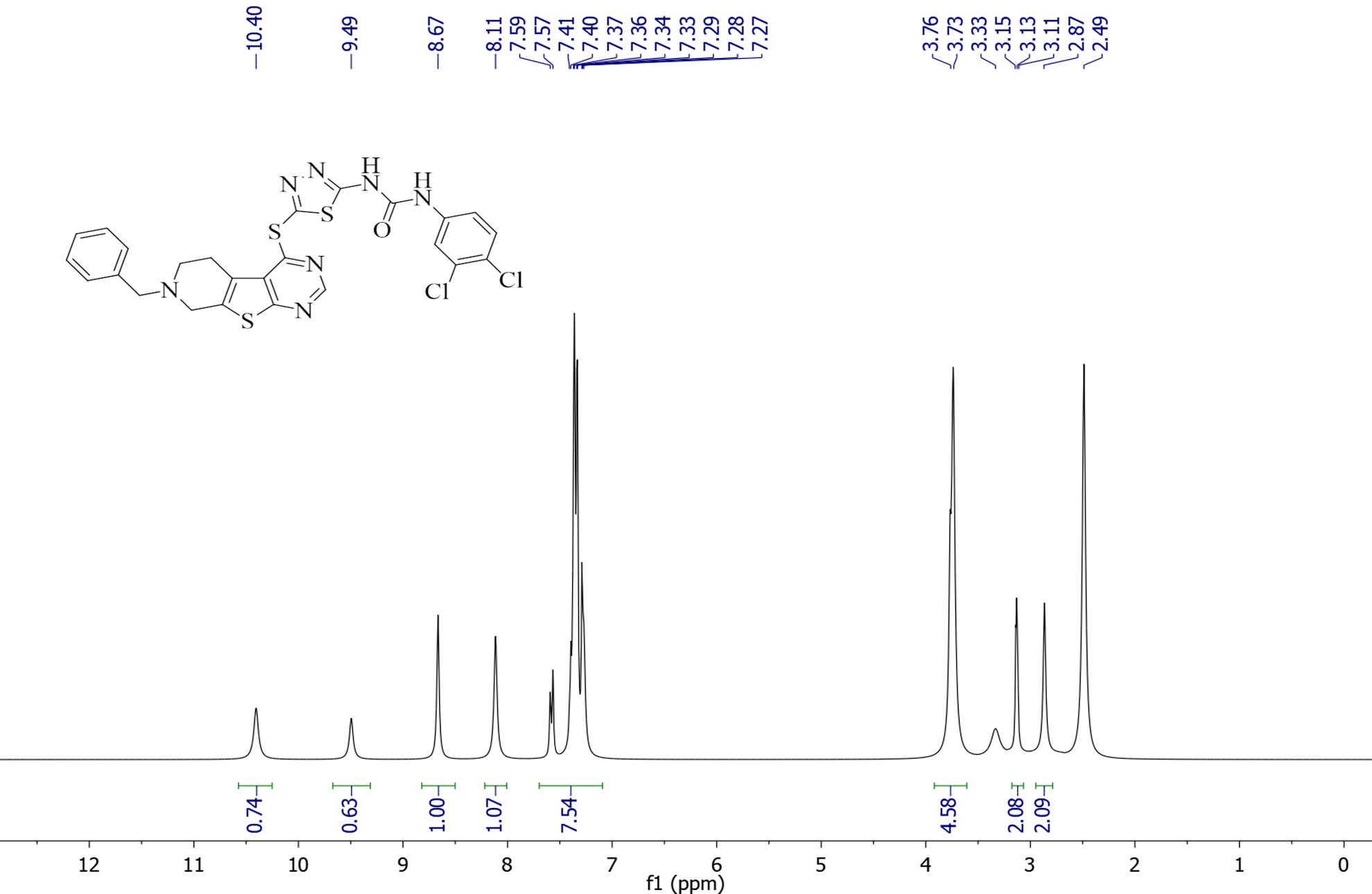
^1^H NMR spectrum of 1-(5-((7-benzyl-5,6,7,8-tetrahydropyrido[4',3':4,5]thieno[2,3-d]pyrimidin-4-yl)thio)-1,3,4-thiadiazol-2-yl)-3-(3,4-dichlorophenyl)urea (**11h**)


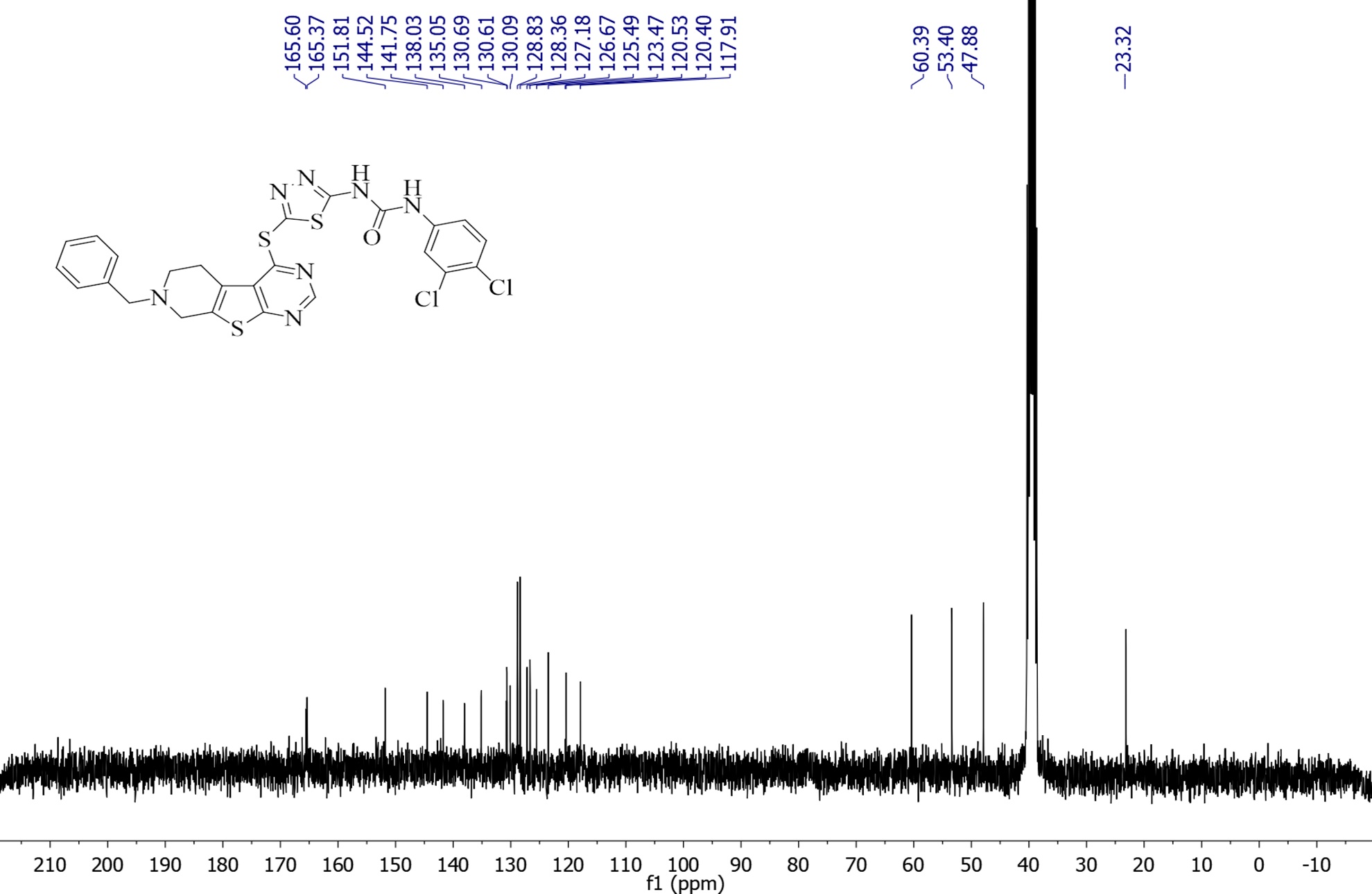
^13^C NMR spectrum of 1-(5-((7-benzyl-5,6,7,8-tetrahydropyrido[4',3':4,5]thieno[2,3-d]pyrimidin-4-yl)thio)-1,3,4-thiadiazol-2-yl)-3-(3,4-dichlorophenyl)urea (**11h**)


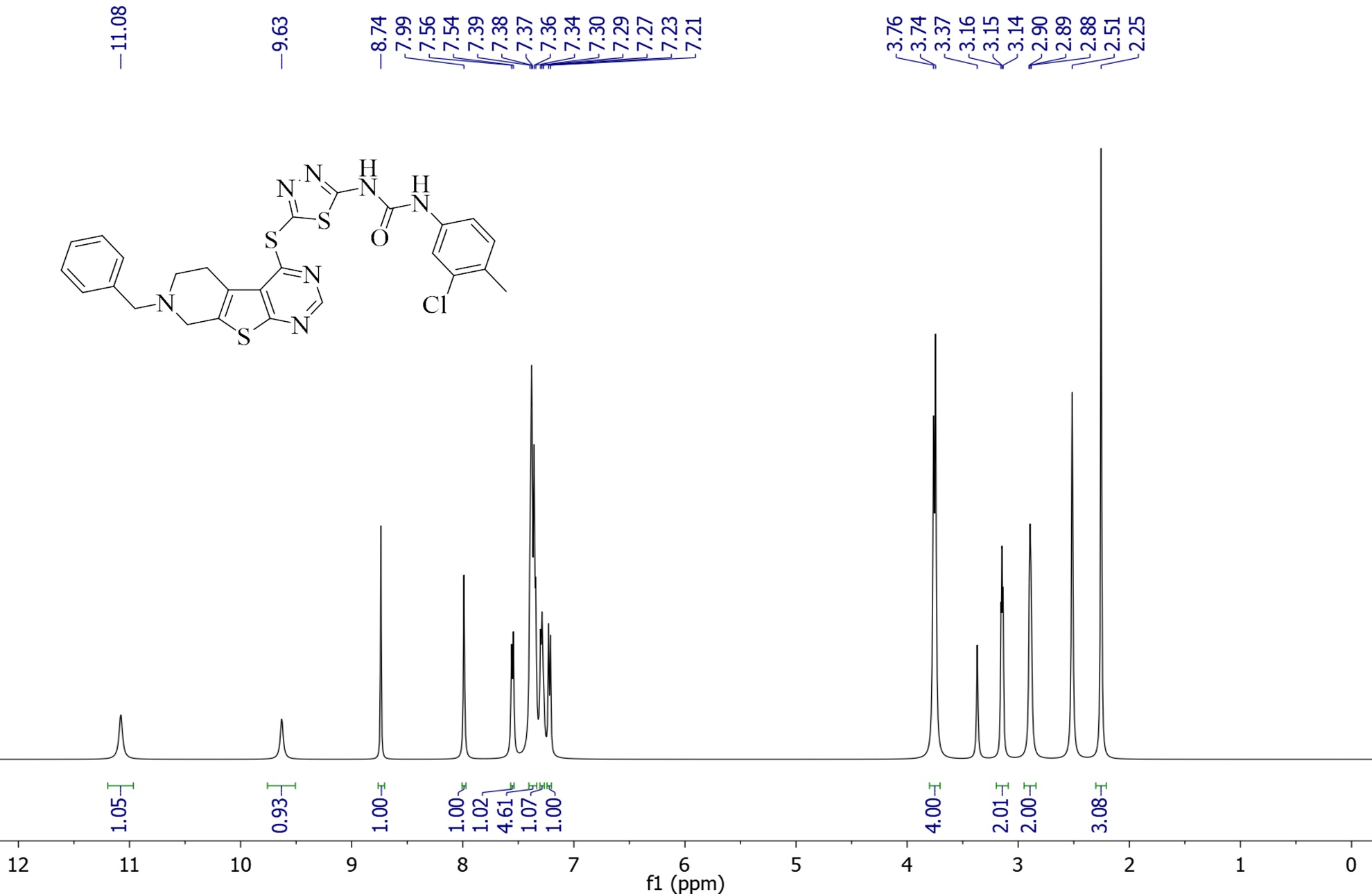
^1^H NMR spectrum of 1-(5-((7-benzyl-5,6,7,8-tetrahydropyrido[4',3':4,5]thieno[2,3-d]pyrimidin-4-yl)thio)-1,3,4-thiadiazol-2-yl)-3-(3-chloro-4-methylphenyl)urea (**11i**)


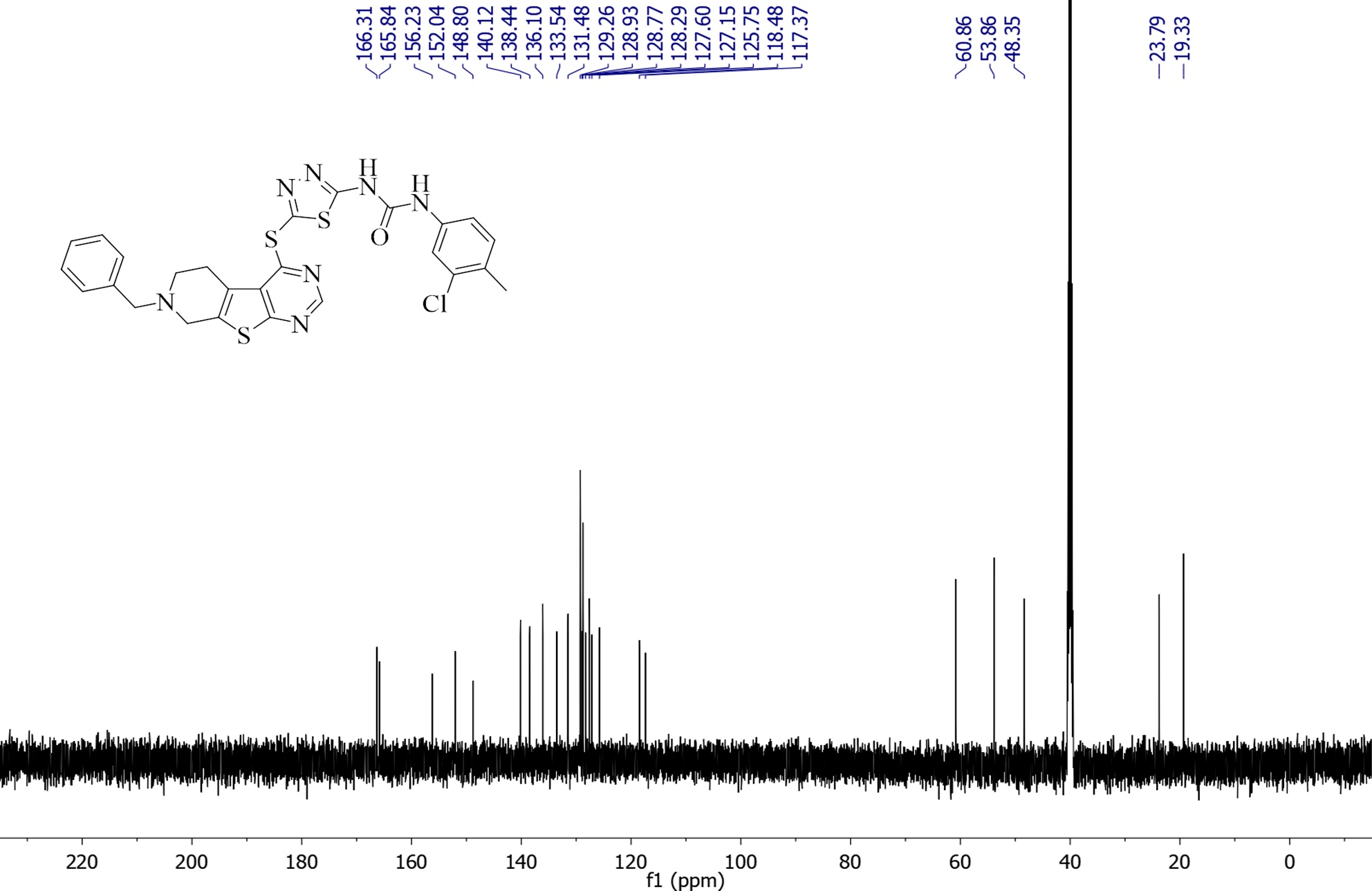
^13^C NMR spectrum of 1-(5-((7-benzyl-5,6,7,8-tetrahydropyrido[4',3':4,5]thieno[2,3-d]pyrimidin-4-yl)thio)-1,3,4-thiadiazol-2-yl)-3-(3-chloro-4-methylphenyl)urea (**11i**)


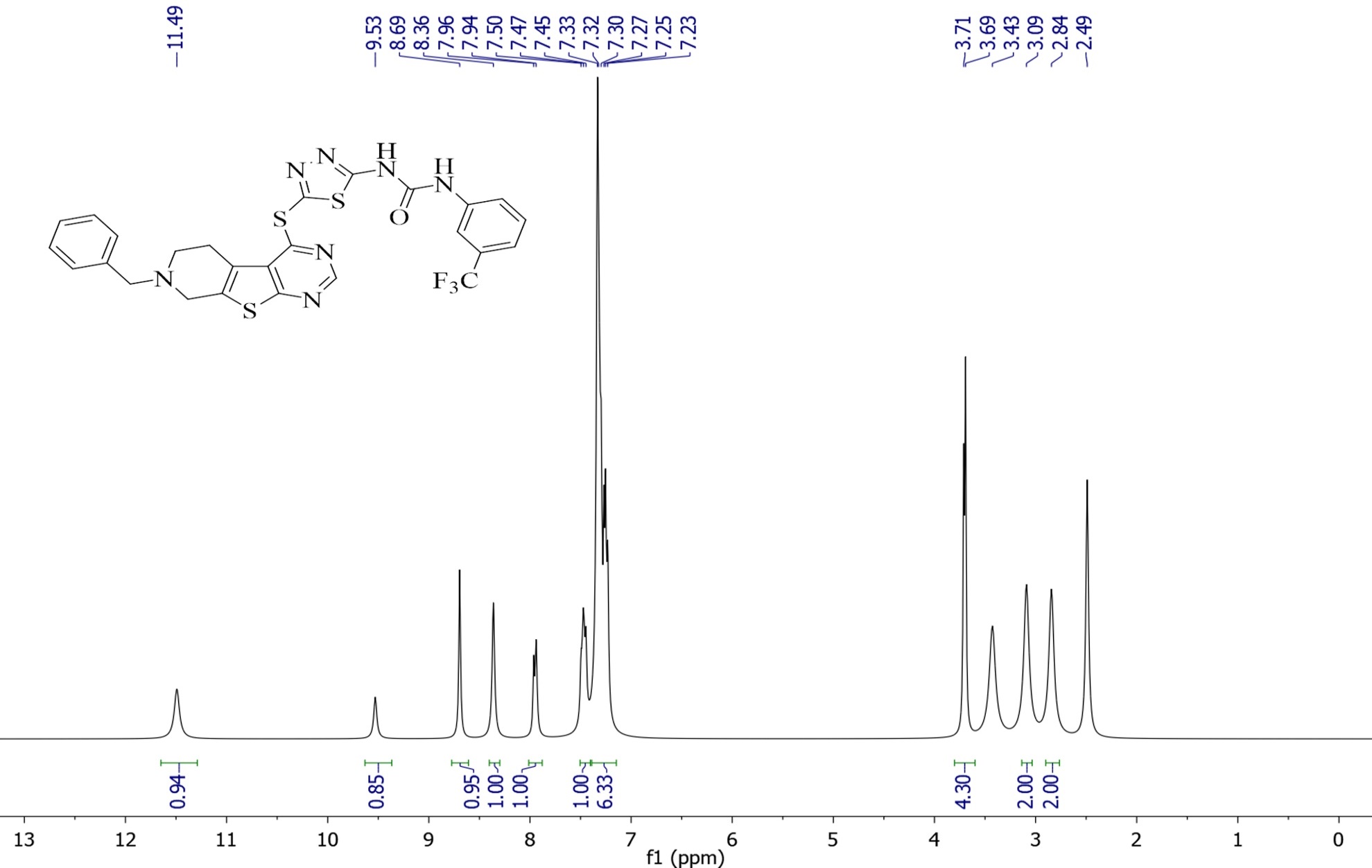
^1^H NMR spectrum of 1-(5-((7-benzyl-5,6,7,8-tetrahydropyrido[4',3':4,5]thieno[2,3-d]pyrimidin-4-yl)thio)-1,3,4-thiadiazol-2-yl)-3-(3-(trifluoromethyl)phenyl)urea (**11j**)


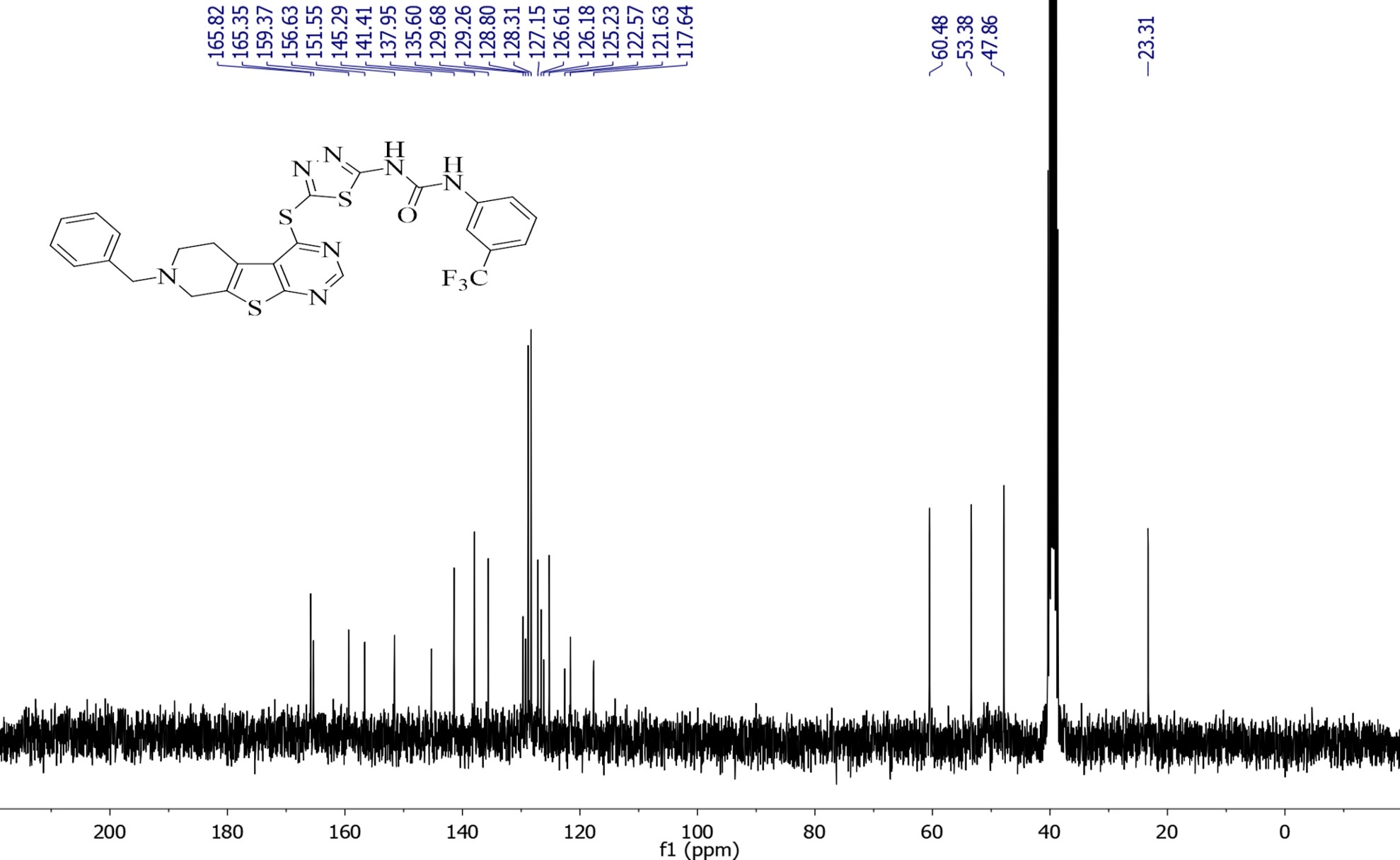
^13^C NMR spectrum of 1-(5-((7-benzyl-5,6,7,8-tetrahydropyrido[4',3':4,5]thieno[2,3-d]pyrimidin-4-yl)thio)-1,3,4-thiadiazol-2-yl)-3-(3-(trifluoromethyl)phenyl)urea (**11j**)


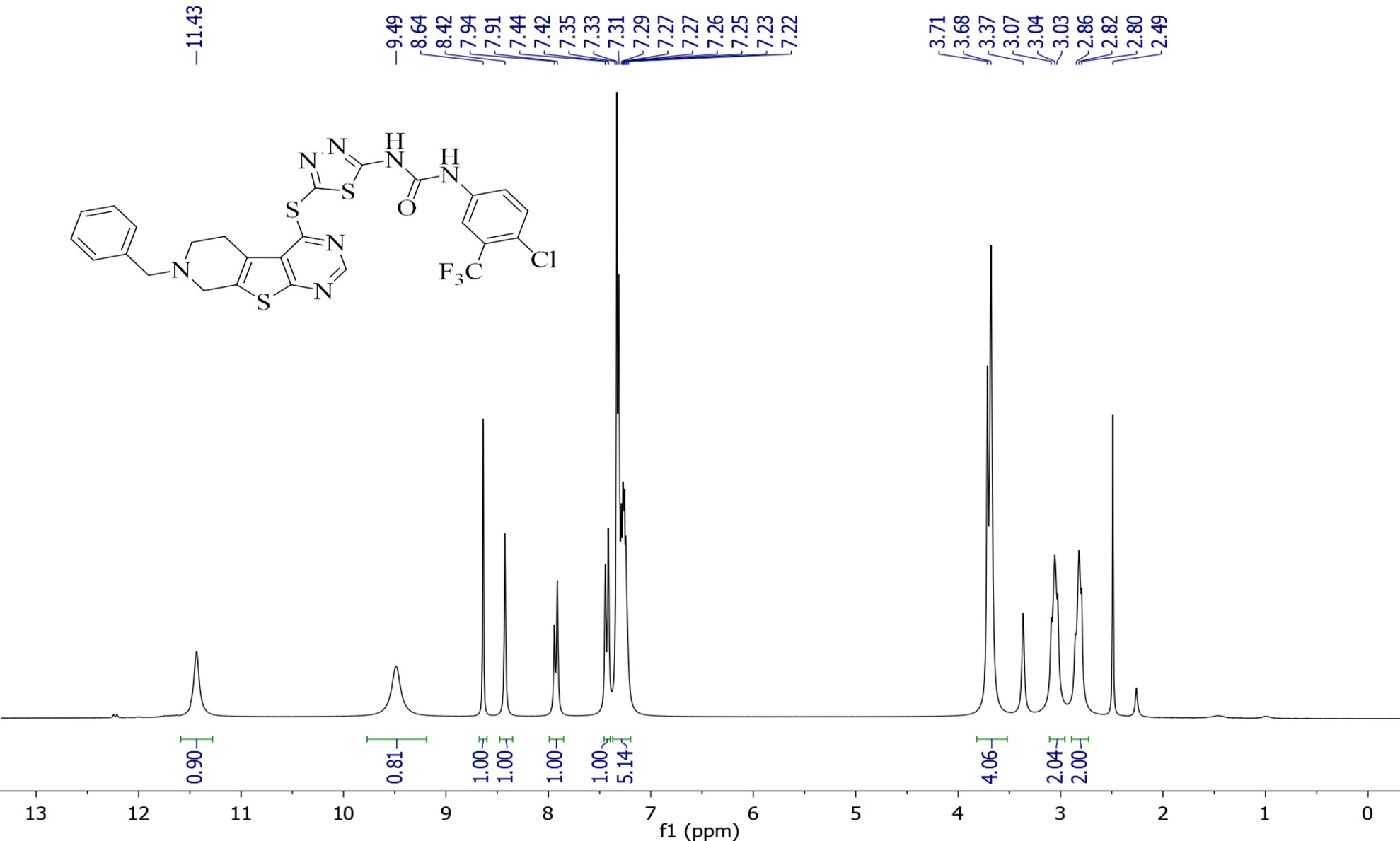
^1^H NMR spectrum of 1-(5-((7-benzyl-5,6,7,8-tetrahydropyrido[4',3':4,5]thieno[2,3-d]pyrimidin-4-yl)thio)-1,3,4-thiadiazol-2-yl)-3-(4-chloro-3-(trifluoromethyl)phenyl)urea (**11k**)


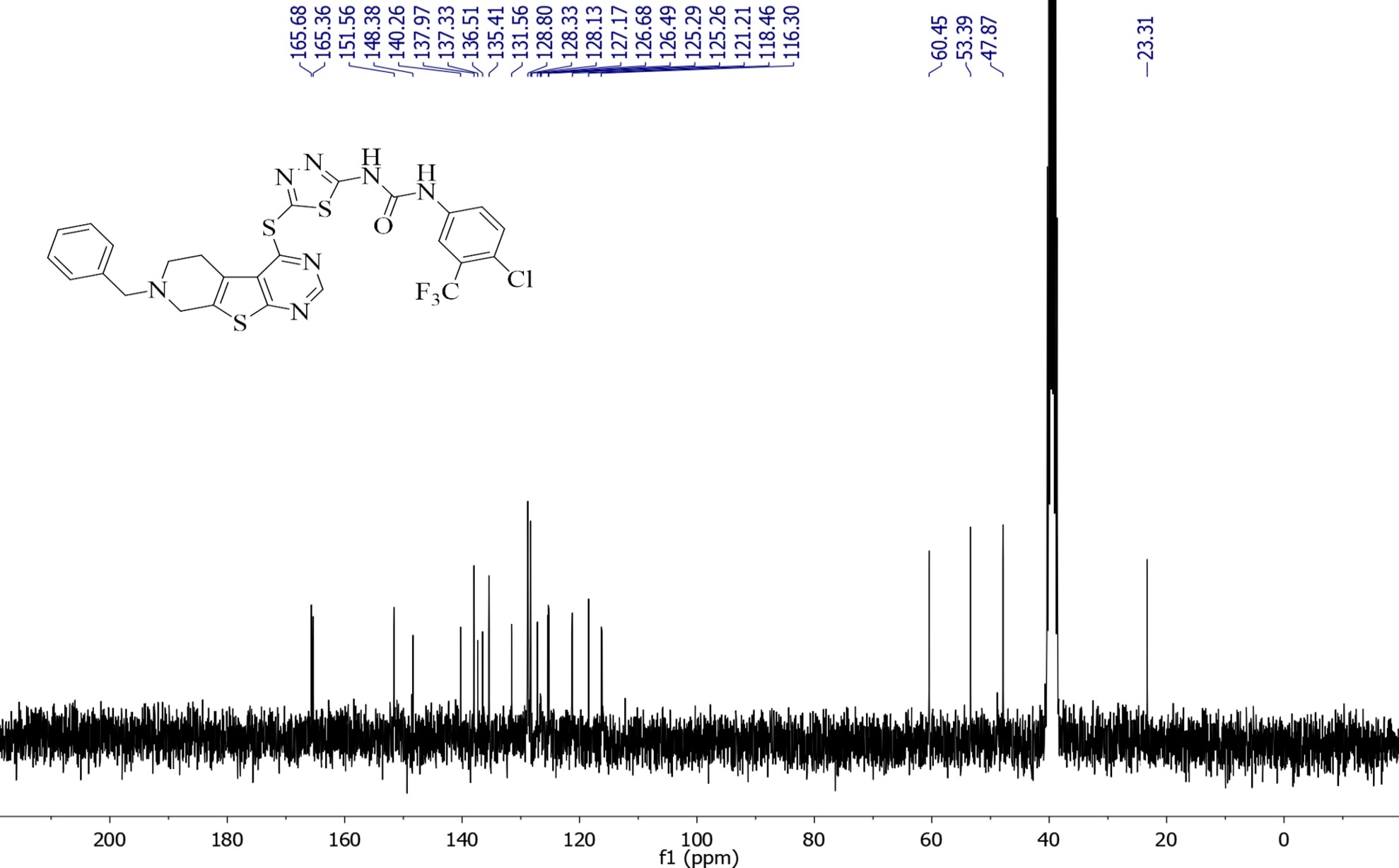
^13^C NMR spectrum of 1-(5-((7-benzyl-5,6,7,8-tetrahydropyrido[4',3':4,5]thieno[2,3-d]pyrimidin-4-yl)thio)-1,3,4-thiadiazol-2-yl)-3-(4-chloro-3-(trifluoromethyl)phenyl)urea (**11k**)


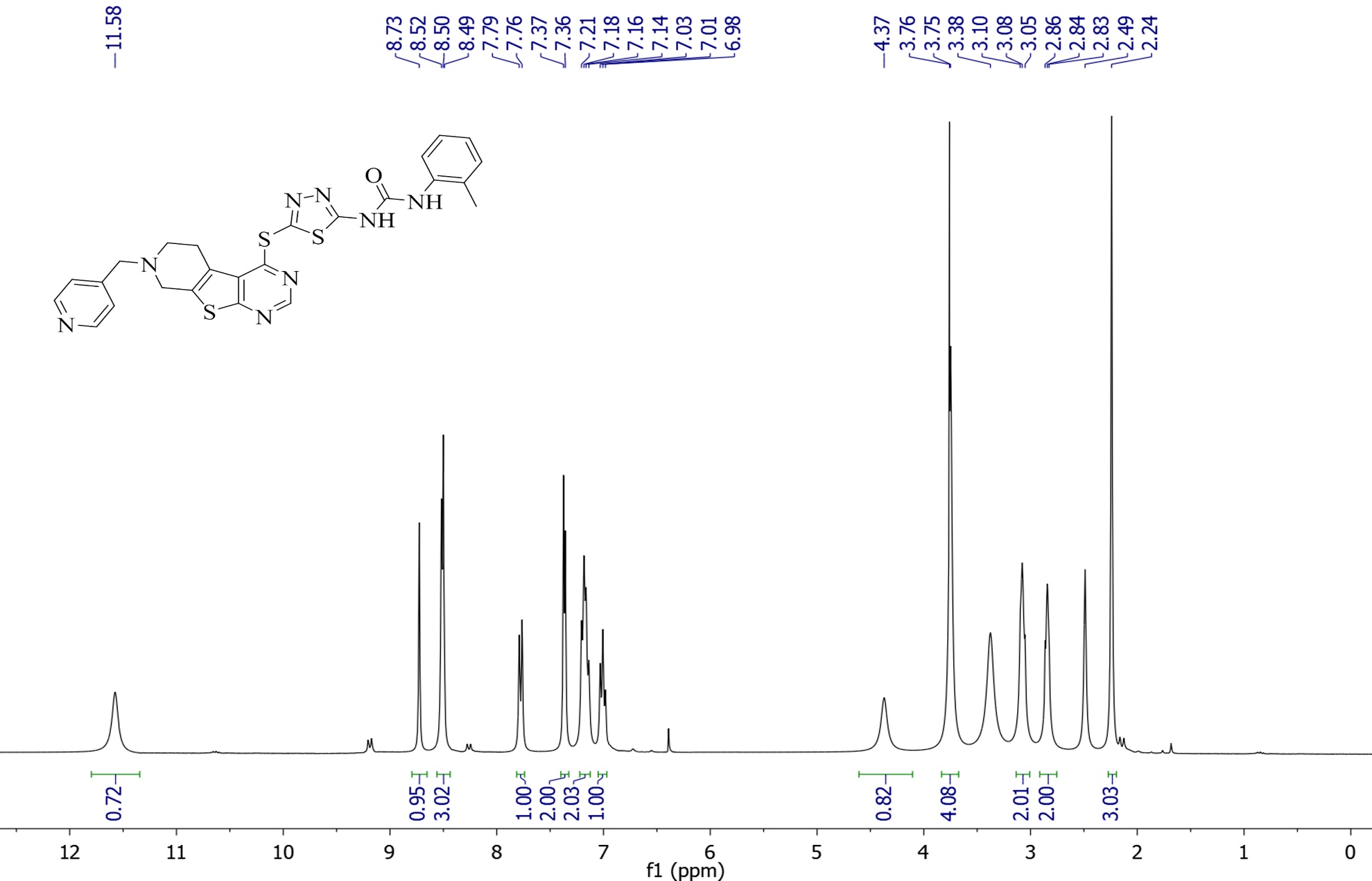
^1^H NMR spectrum of 1-(5-((7-(pyridin-4-ylmethyl)-5,6,7,8-tetrahydropyrido[4',3':4,5]thieno[2,3-d]pyrimidin-4-yl)thio)-1,3,4-thiadiazol-2-yl)-3-(o-tolyl)urea (**11l**)


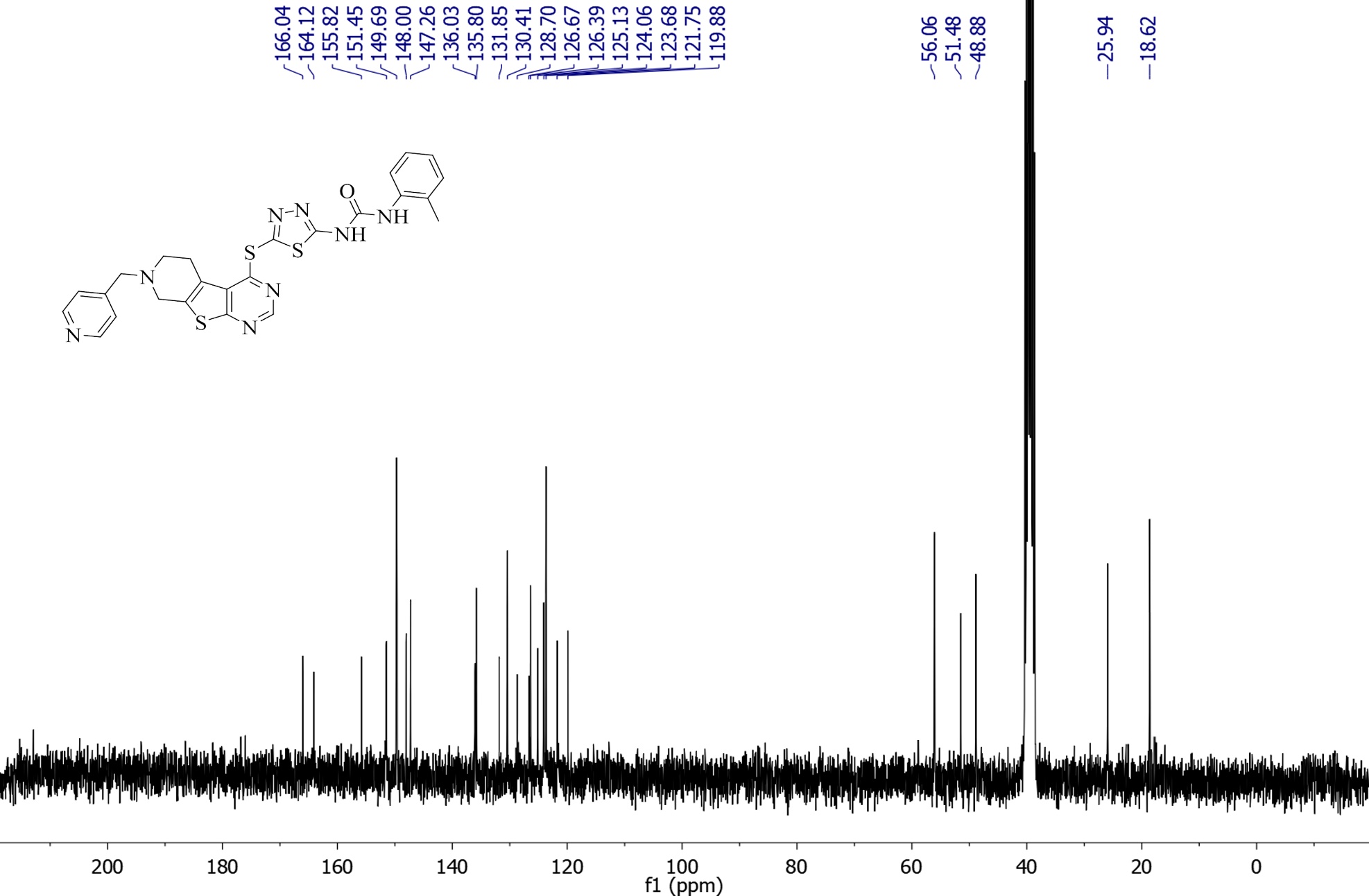
^13^C NMR spectrum of 1-(5-((7-(pyridin-4-ylmethyl)-5,6,7,8-tetrahydropyrido[4',3':4,5]thieno[2,3-d]pyrimidin-4-yl)thio)-1,3,4-thiadiazol-2-yl)-3-(o-tolyl)urea (**11l**)


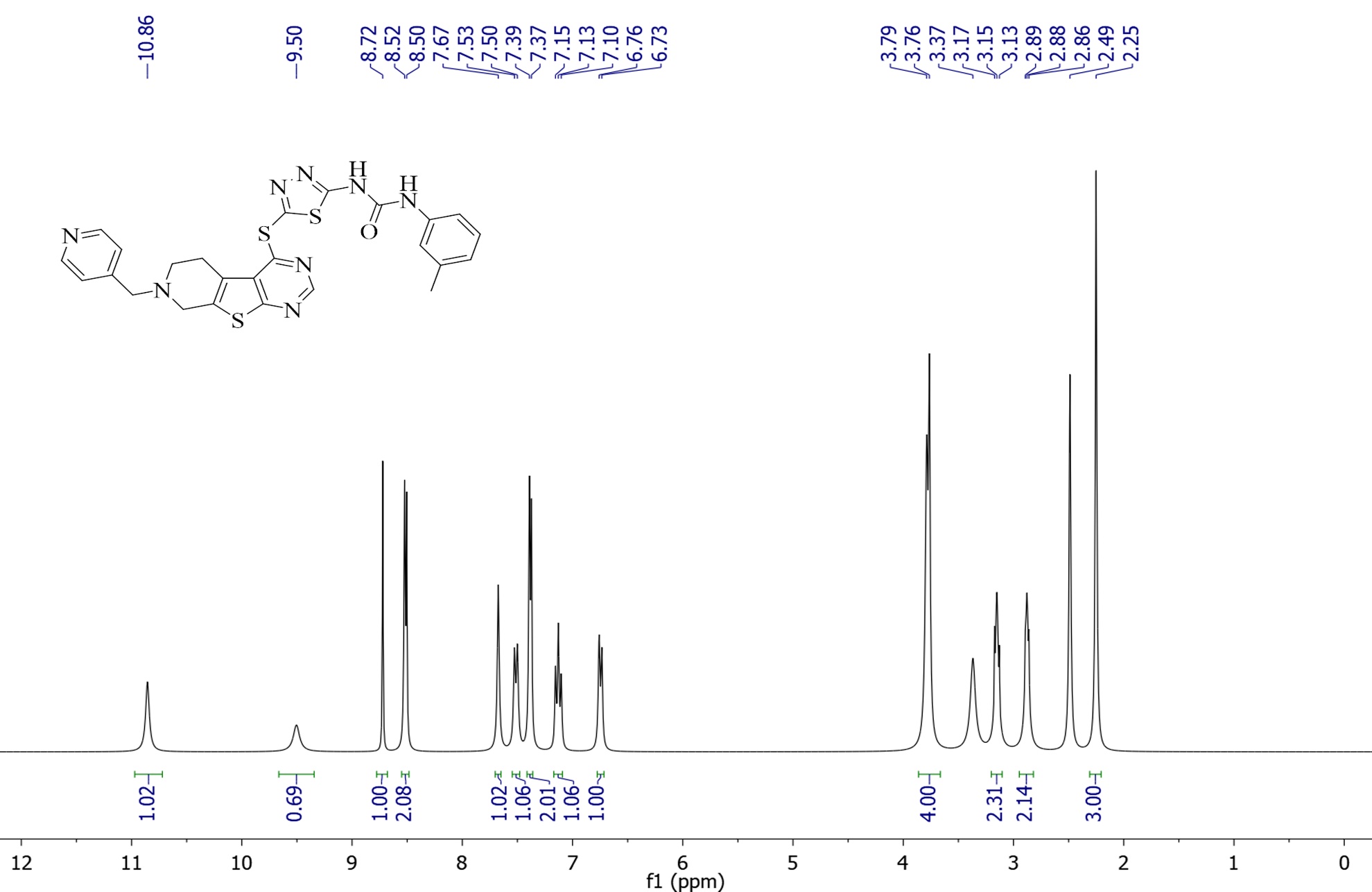
^1^H NMR spectrum of 1-(5-((7-(pyridin-4-ylmethyl)-5,6,7,8-tetrahydropyrido[4',3':4,5]thieno[2,3-d]pyrimidin-4-yl)thio)-1,3,4-thiadiazol-2-yl)-3-(m-tolyl)urea (**11m**


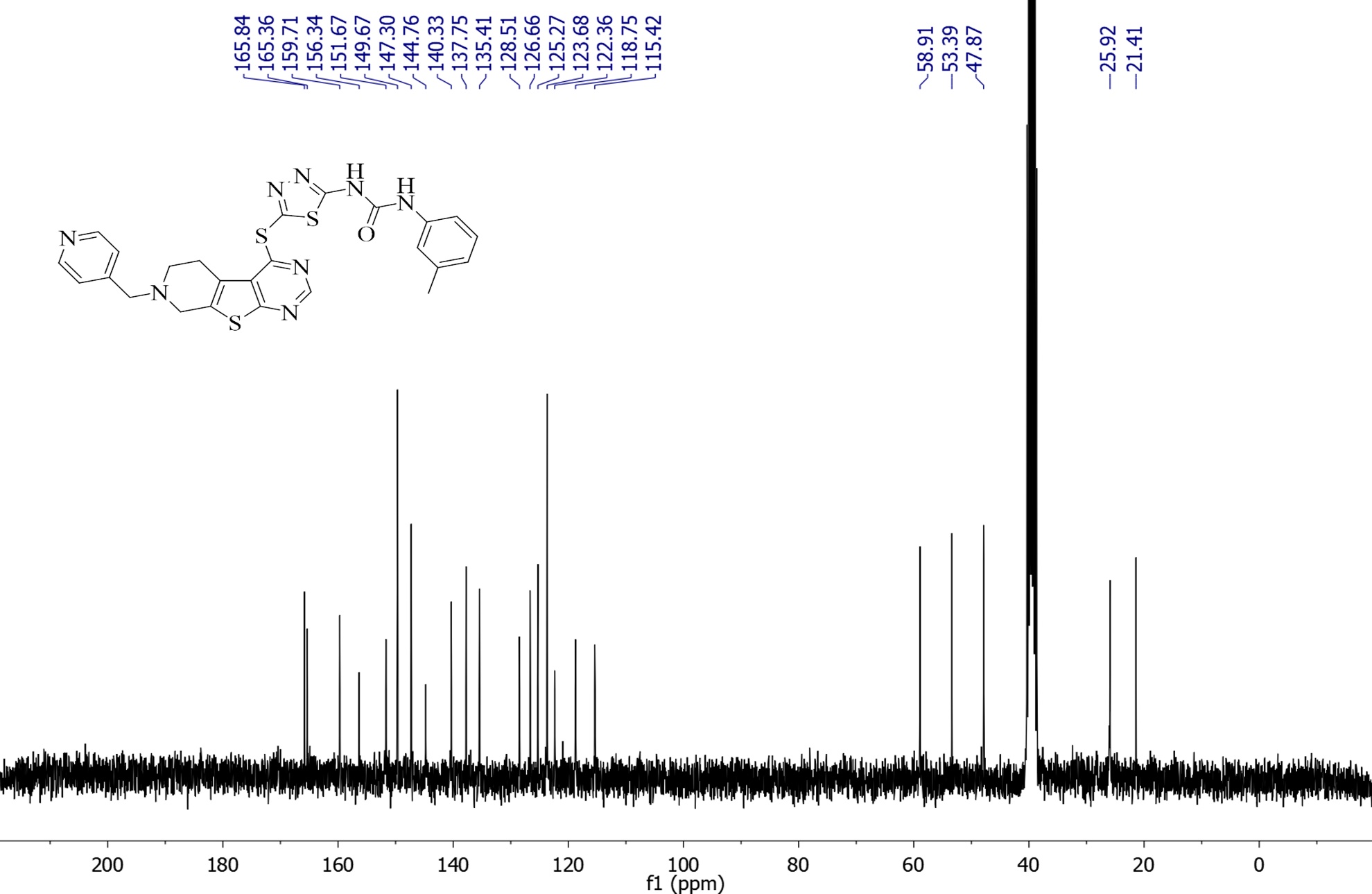
^13^C NMR spectrum of 1-(5-((7-(pyridin-4-ylmethyl)-5,6,7,8-tetrahydropyrido[4',3':4,5]thieno[2,3-d]pyrimidin-4-yl)thio)-1,3,4-thiadiazol-2-yl)-3-(m-tolyl)urea (**11m**)


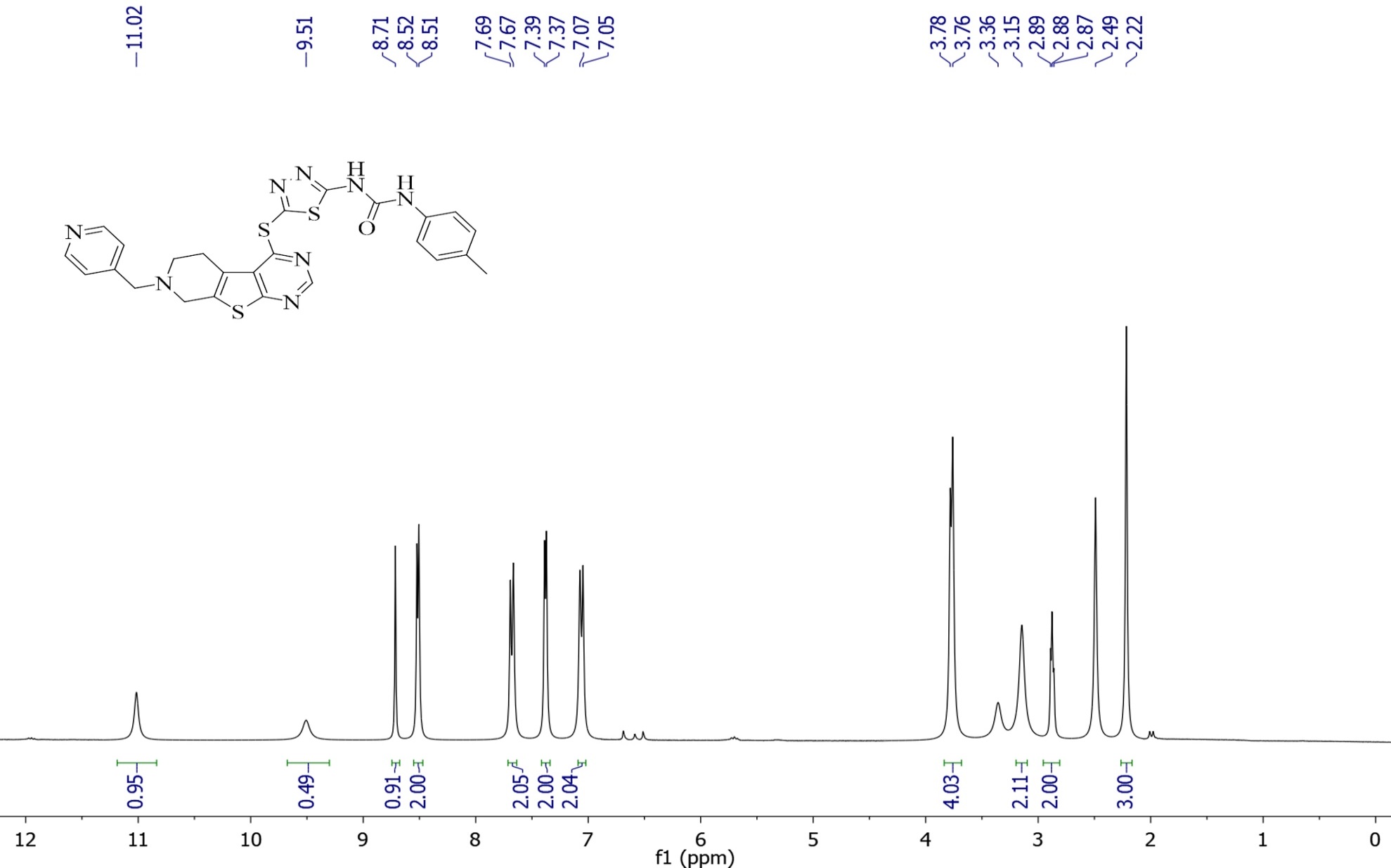
^1^H NMR spectrum of 1-(5-((7-(pyridin-4-ylmethyl)-5,6,7,8-tetrahydropyrido[4',3':4,5]thieno[2,3-d]pyrimidin-4-yl)thio)-1,3,4-thiadiazol-2-yl)-3-(p-tolyl)urea (**11n**)


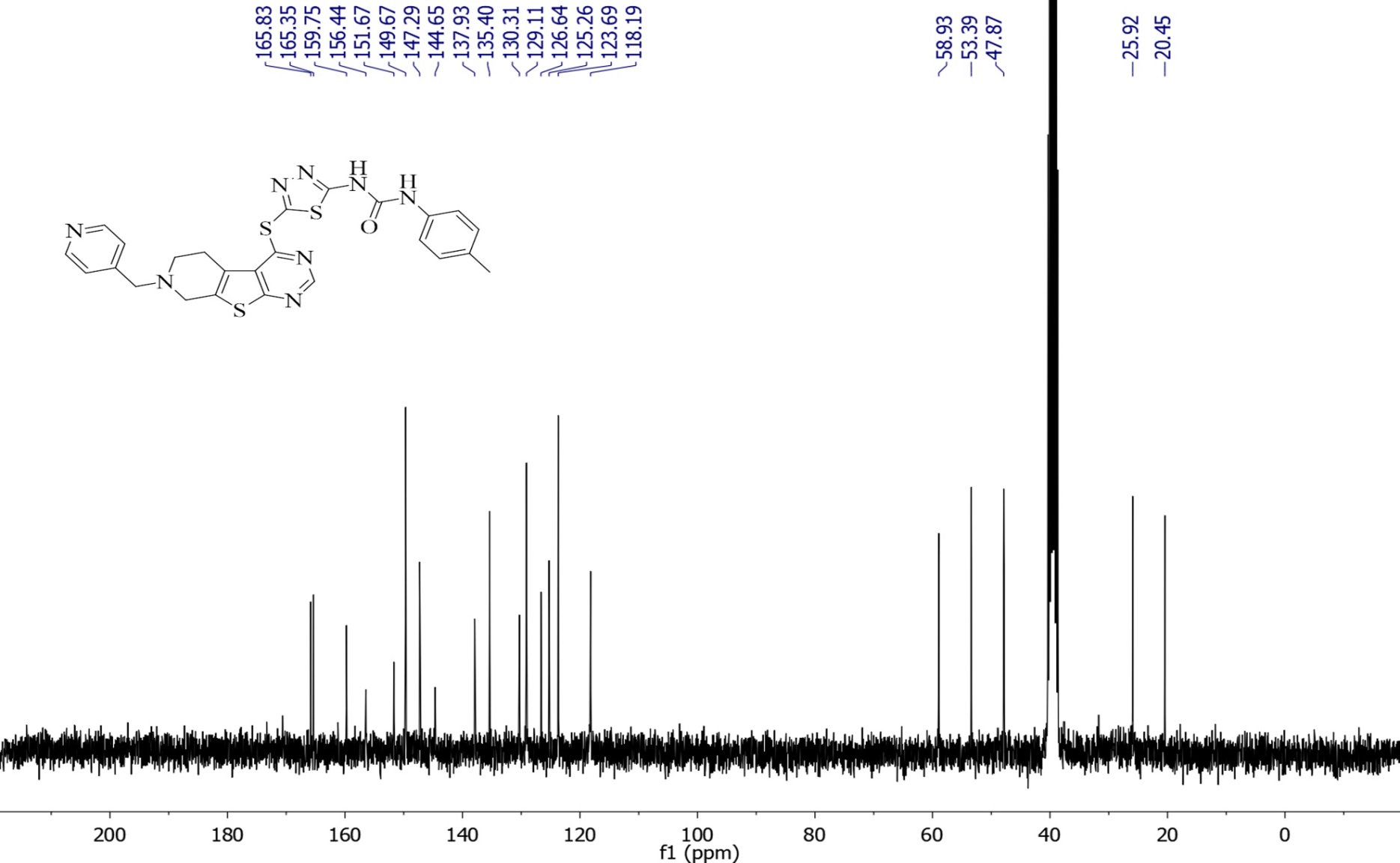
^13^C NMR spectrum of 1-(5-((7-(pyridin-4-ylmethyl)-5,6,7,8-tetrahydropyrido[4',3':4,5]thieno[2,3-d]pyrimidin-4-yl)thio)-1,3,4-thiadiazol-2-yl)-3-(p-tolyl)urea (**11n**)


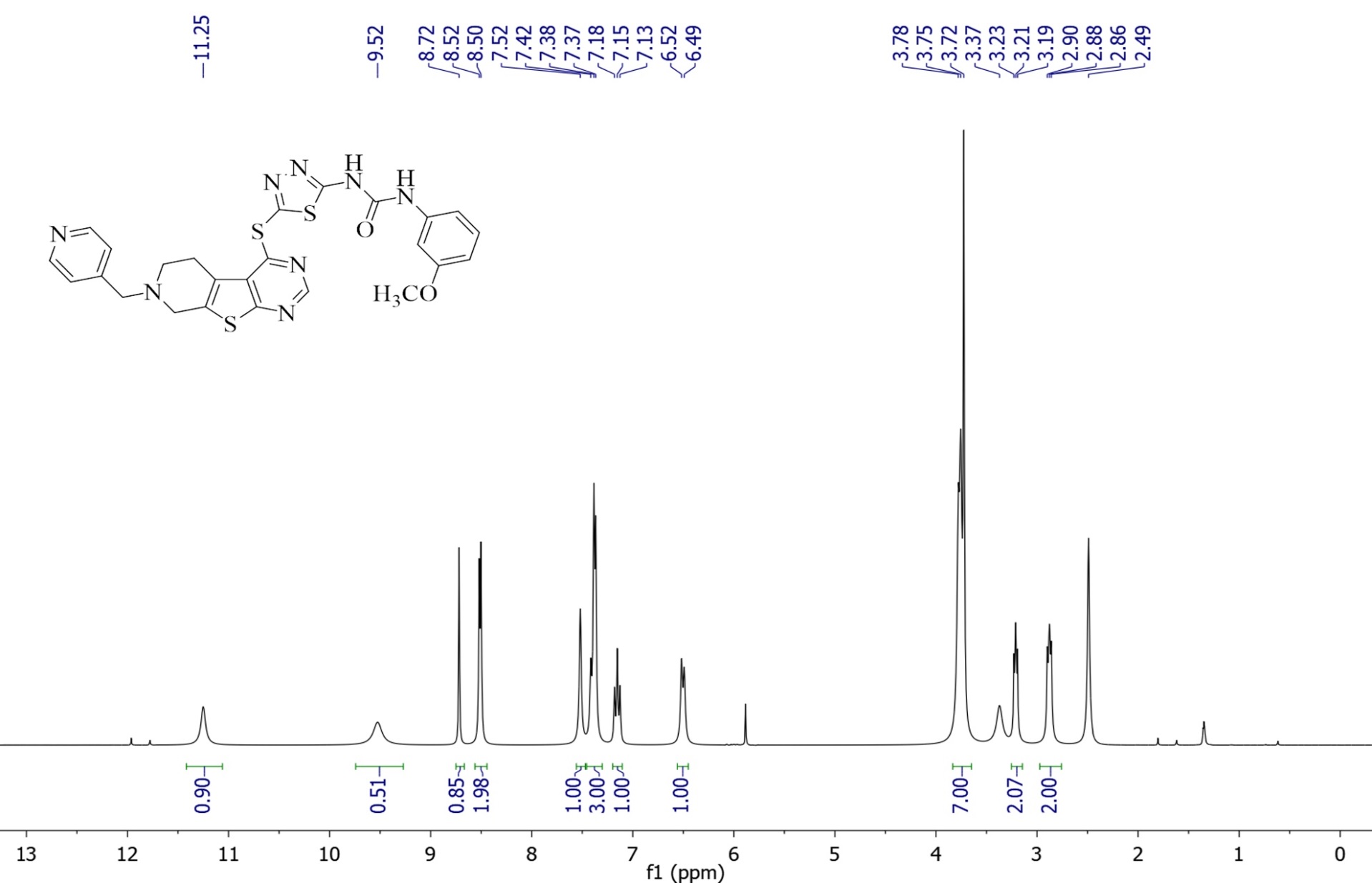
^1^H NMR spectrum of 1-(3-methoxyphenyl)-3-(5-((7-(pyridin-4-ylmethyl)-5,6,7,8-tetrahydropyrido[4',3':4,5]thieno[2,3-d]pyrimidin-4-yl)thio)-1,3,4-thiadiazol-2-yl)urea (**11o**)

**
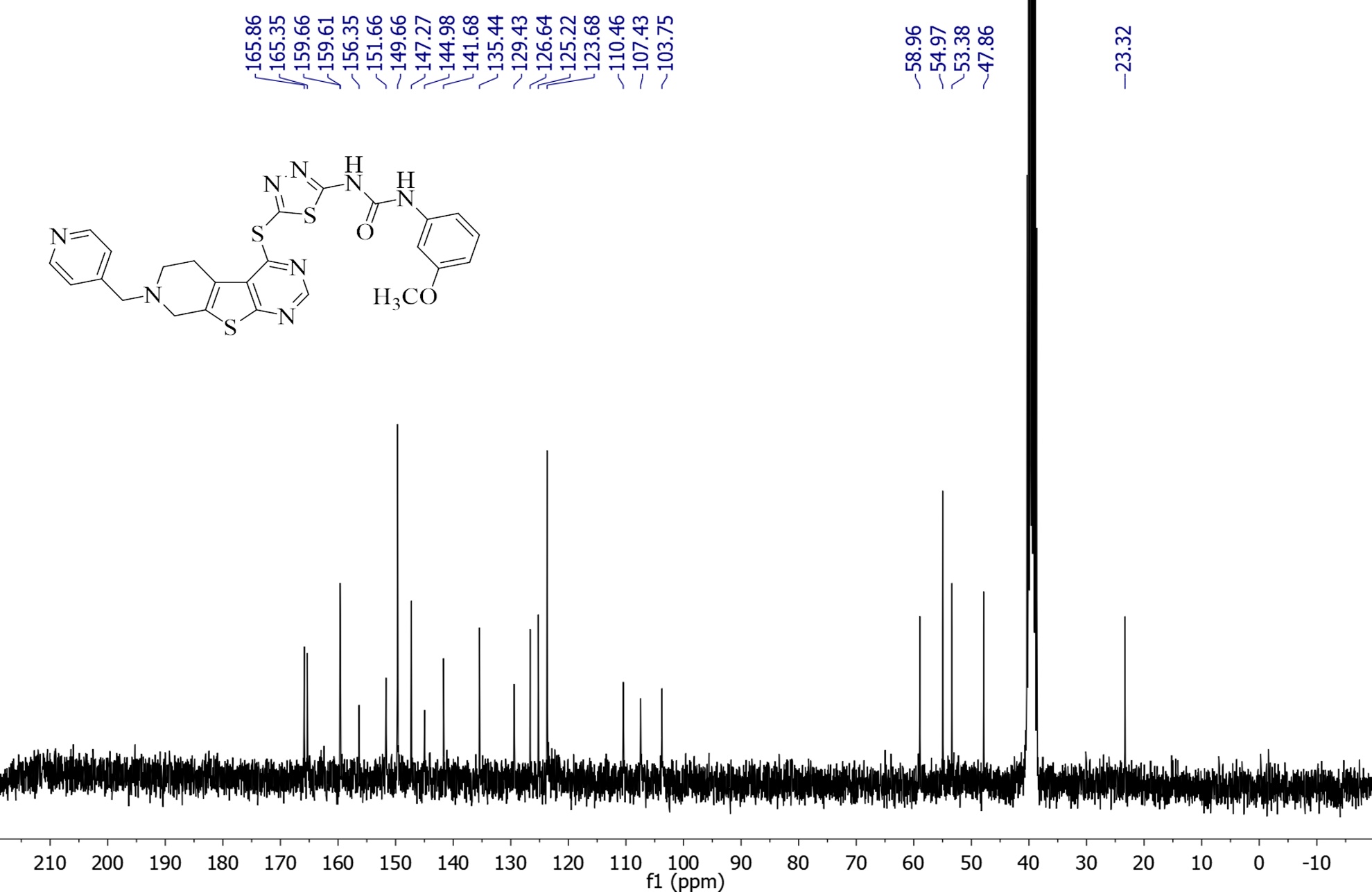
**^13^C NMR spectrum of 1-(3-methoxyphenyl)-3-(5-((7-(pyridin-4-ylmethyl)-5,6,7,8-tetrahydropyrido[4',3':4,5]thieno[2,3-d]pyrimidin-4-yl)thio)-1,3,4-thiadiazol-2-yl)urea (**11o**)^1^H NMR spectrum of 1-(4-methoxyphenyl)-3-(5-((7-(pyridin-4-ylmethyl)-5,6,7,8-tetrahydropyrido[4',3':4,5]thieno[2,3-*d*]pyrimidin-4-
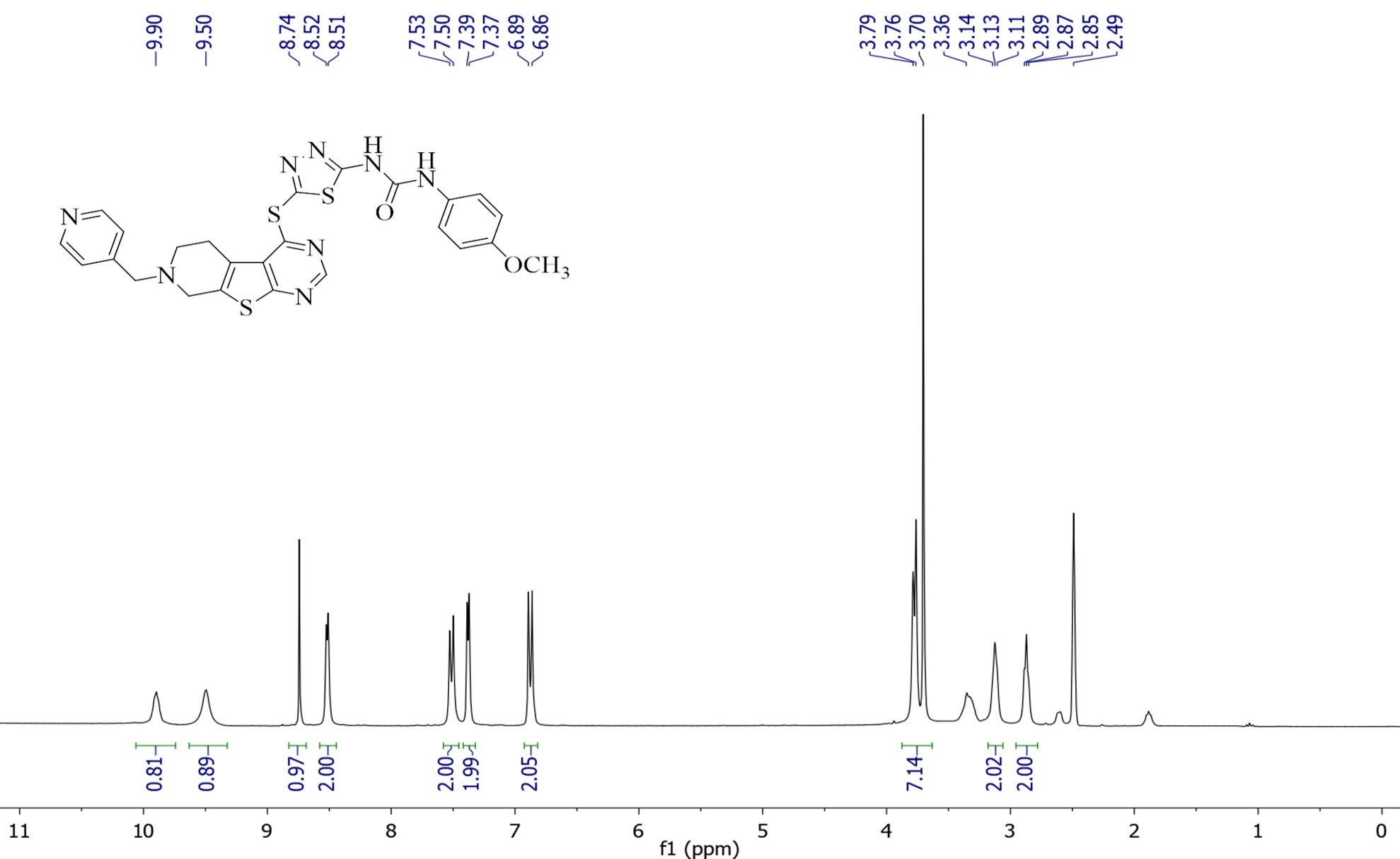
yl)thio)-1,3,4-thiadiazol-2-yl)urea (**11p**)


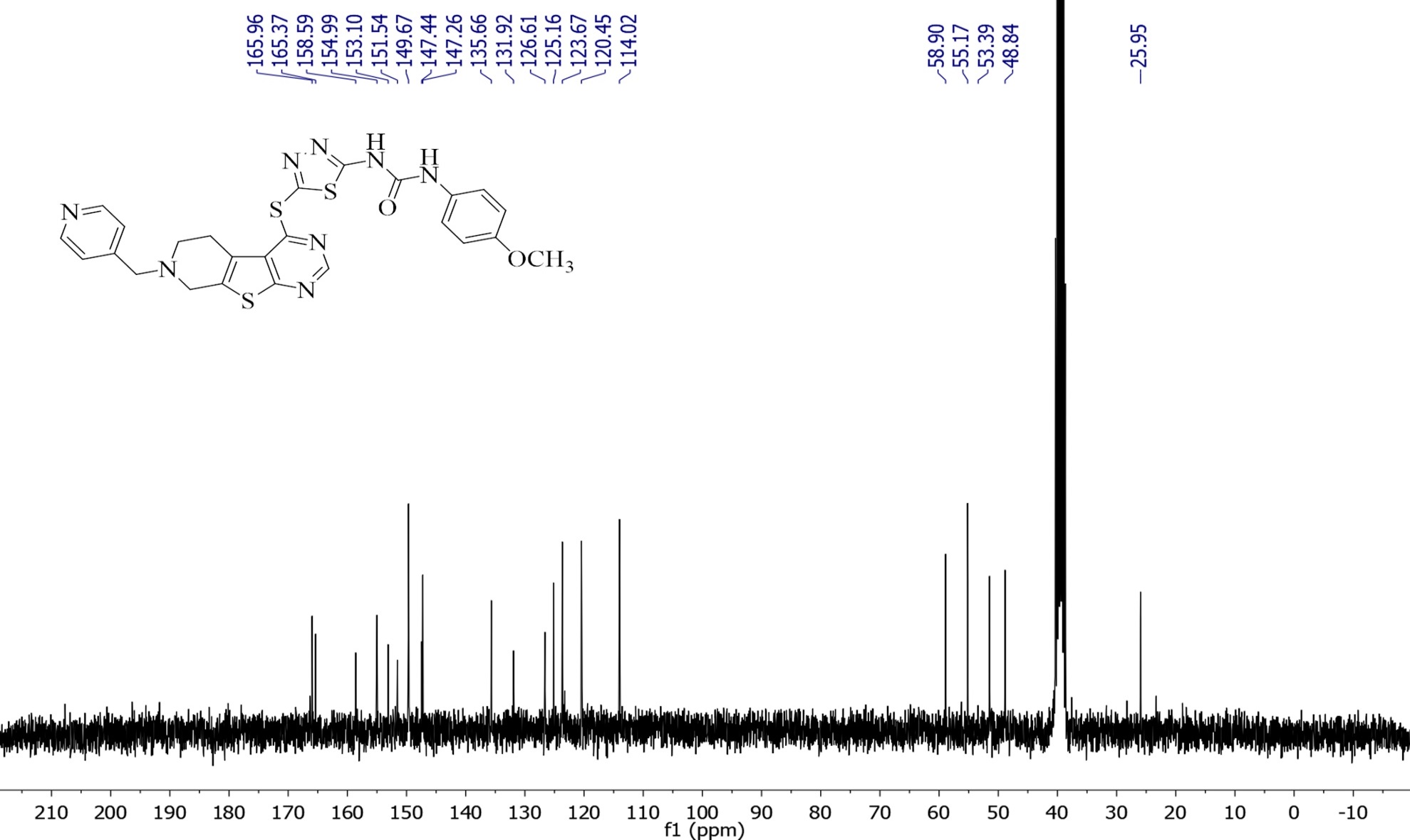
^13^C NMR spectrum of 1-(4-methoxyphenyl)-3-(5-((7-(pyridin-4-ylmethyl)-5,6,7,8-tetrahydropyrido[4',3':4,5]thieno[2,3-*d*]pyrimidin 4-


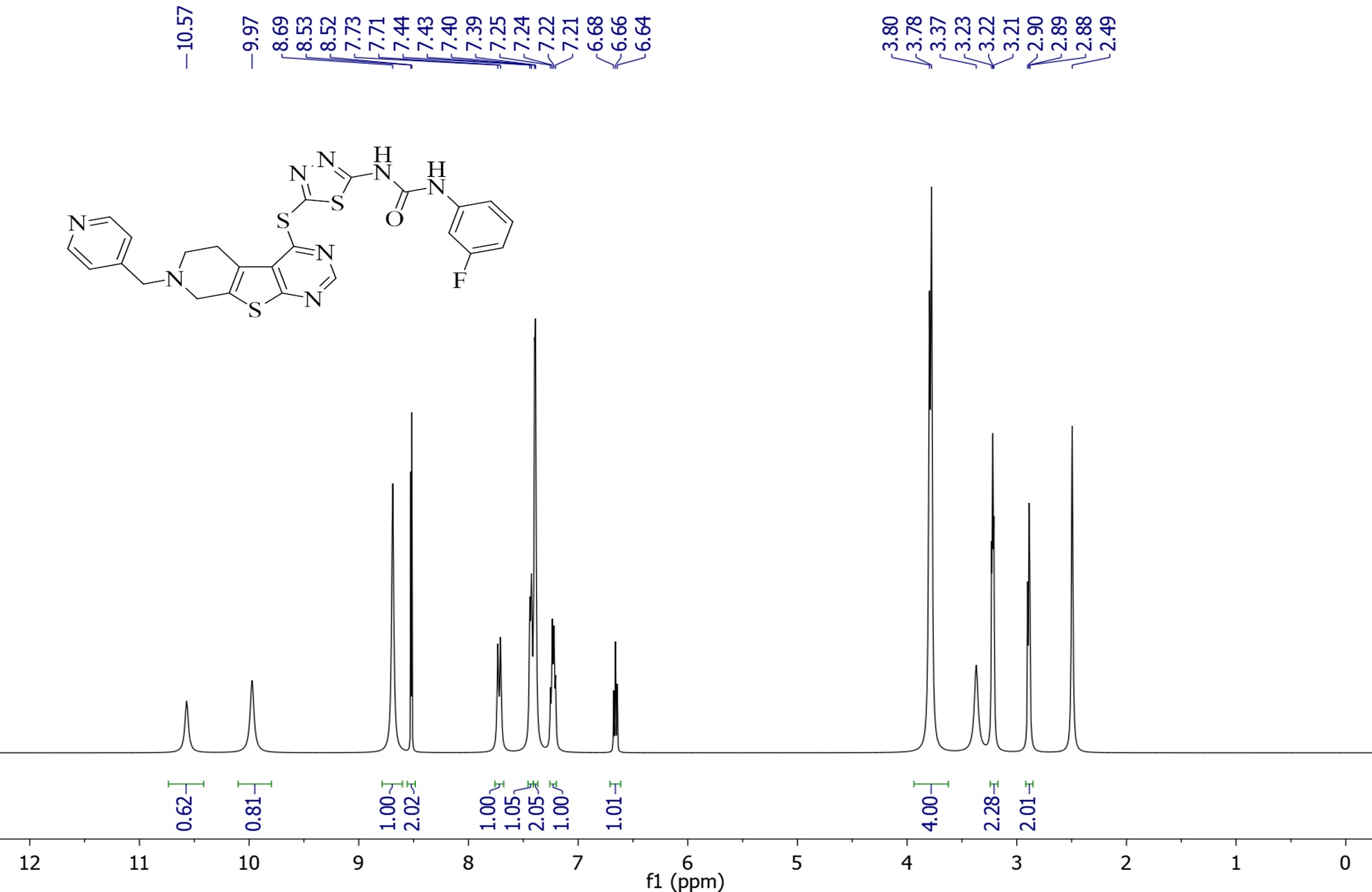
^1^H NMR spectrum of 1-(3-fluorophenyl)-3-(5-((7-(pyridin-4-ylmethyl)-5,6,7,8-tetrahydropyrido[4',3':4,5]thieno[2,3-d]pyrimidin-4-yl)thio)-1,3,4-thiadiazol-2-yl)urea (**11q**)


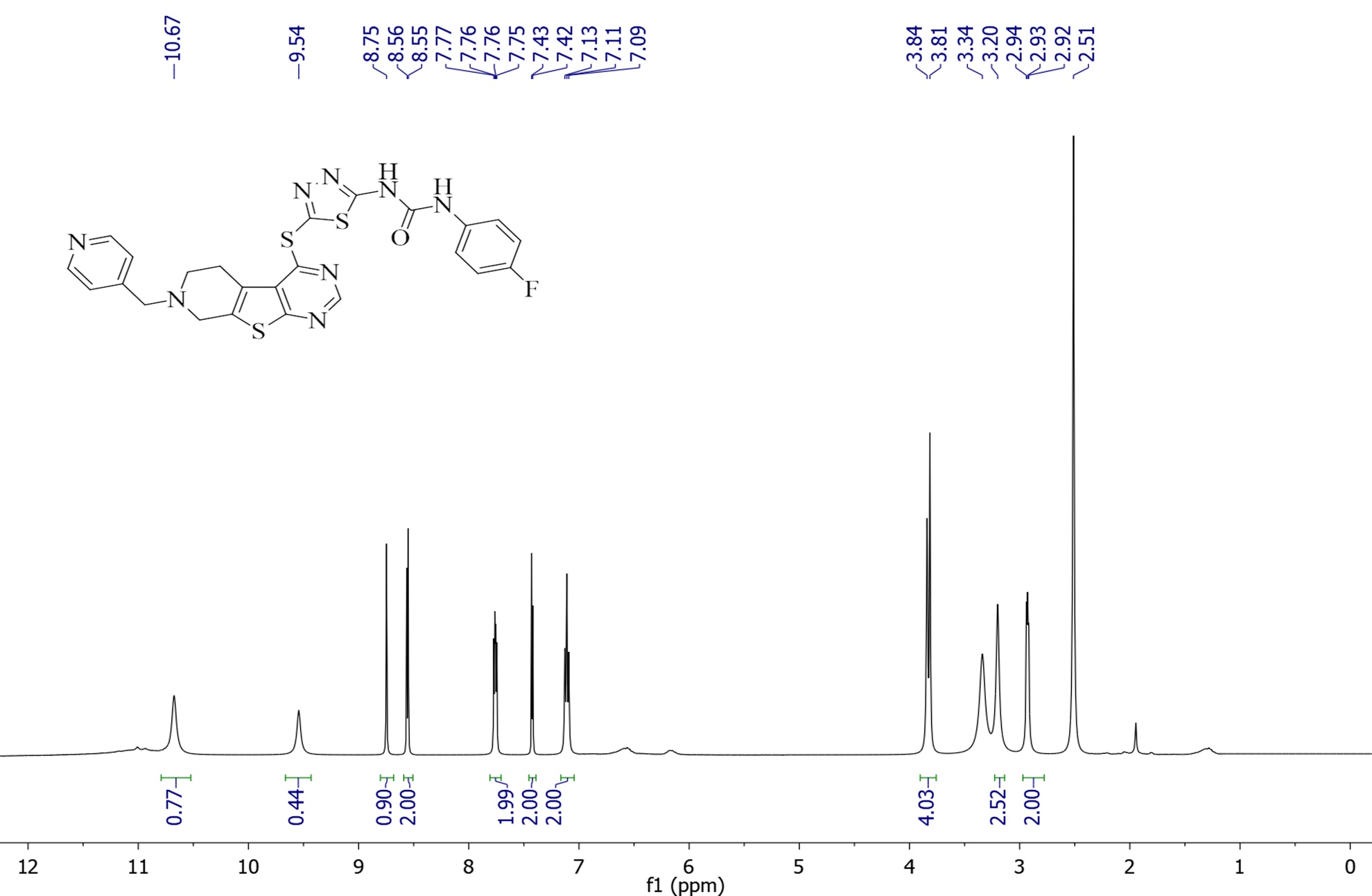
^1^H NMR spectrum of 1-(4-fluorophenyl)-3-(5-((7-(pyridin-4-ylmethyl)-5,6,7,8-tetrahydropyrido[4',3':4,5]thieno[2,3-d]pyrimidin-4-yl)thio)-1,3,4-thiadiazol-2-yl)urea (**11r**)


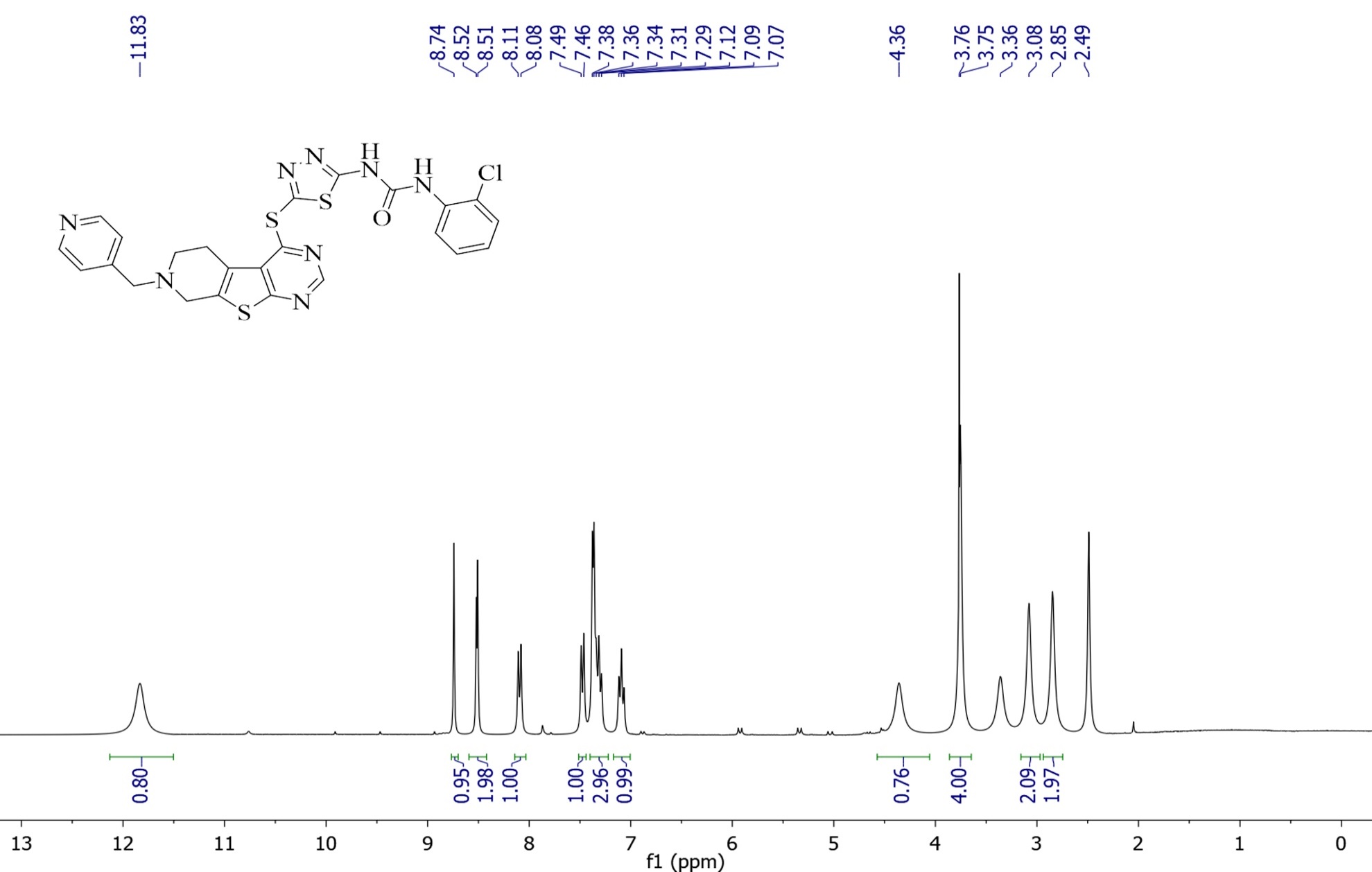
^1^H NMR spectrum of 1-(2-chlorophenyl)-3-(5-((7-(pyridin-4-ylmethyl)-5,6,7,8-tetrahydropyrido[4',3':4,5]thieno[2,3-d]pyrimidin-4-yl)thio)-1,3,4-thiadiazol-2-yl)urea (**11s**)


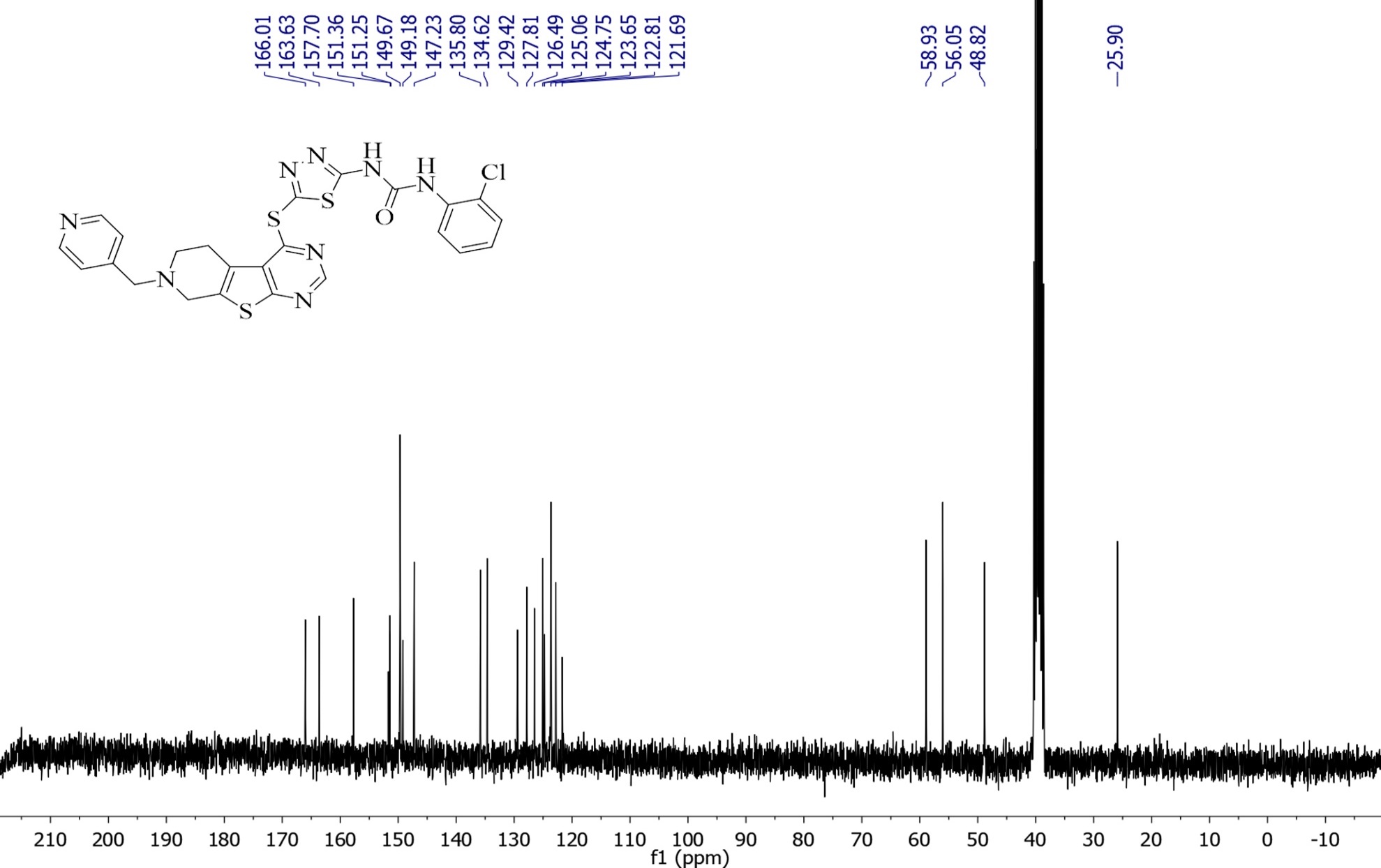
^13^C NMR spectrum of spectrum of 1-(2-chlorophenyl)-3-(5-((7-(pyridin-4-ylmethyl)-5,6,7,8-tetrahydropyrido[4',3':4,5]thieno[2,3-d]pyrimidin-4-yl)thio)-1,3,4-thiadiazol-2-yl)urea (**11s**)


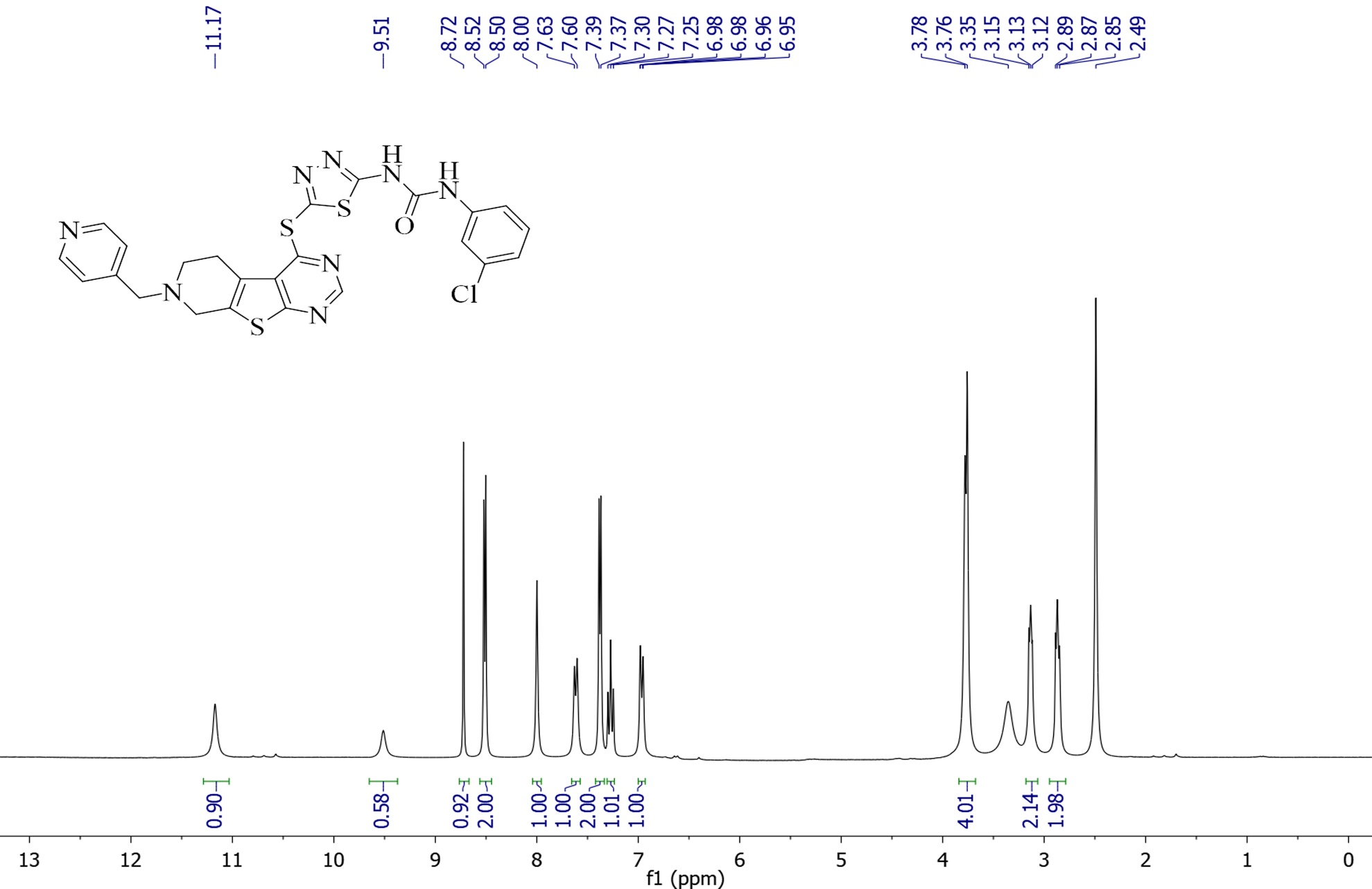
^1^H NMR spectrum of 1-(3-chlorophenyl)-3-(5-((7-(pyridin-4-ylmethyl)-5,6,7,8-tetrahydropyrido[4',3':4,5]thieno[2,3-d]pyrimidin-4

**
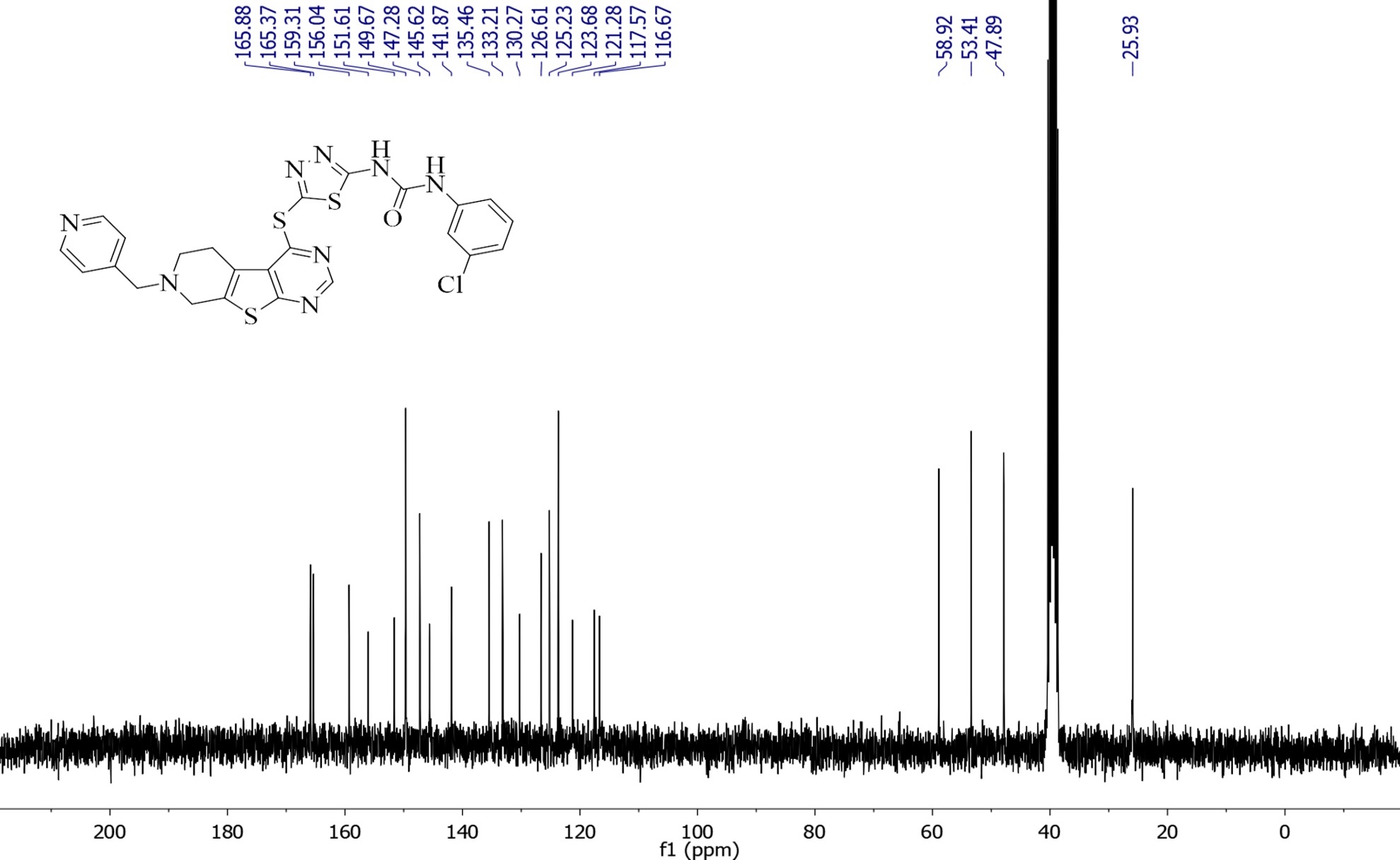
**^13^C NMR spectrum of 1-(3-chlorophenyl)-3-(5-((7-(pyridin-4-ylmethyl)-5,6,7,8-tetrahydropyrido[4',3':4,5]thieno[2,3-d]pyrimidin-4-yl)thio)-1,3,4-thiadiazol-2-yl)urea (**11t**)


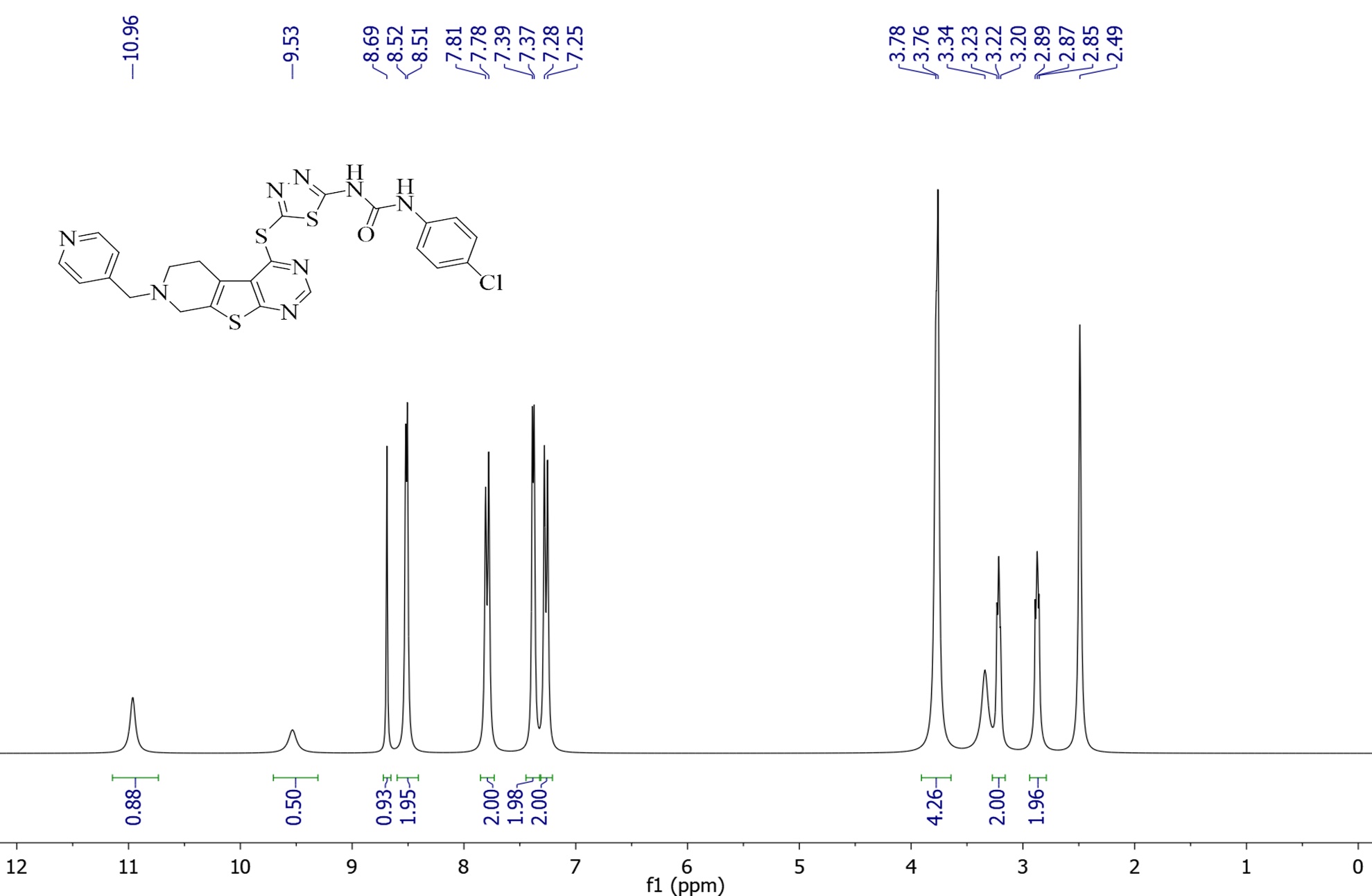
^1^H NMR spectrum of 1-(4-chlorophenyl)-3-(5-((7-(pyridin-4-ylmethyl)-5,6,7,8-tetrahydropyrido[4',3':4,5]thieno[2,3-d]pyrimidin-4-yl)thio)-1,3,4-thiadiazol-2-yl)urea (**11u**)

**
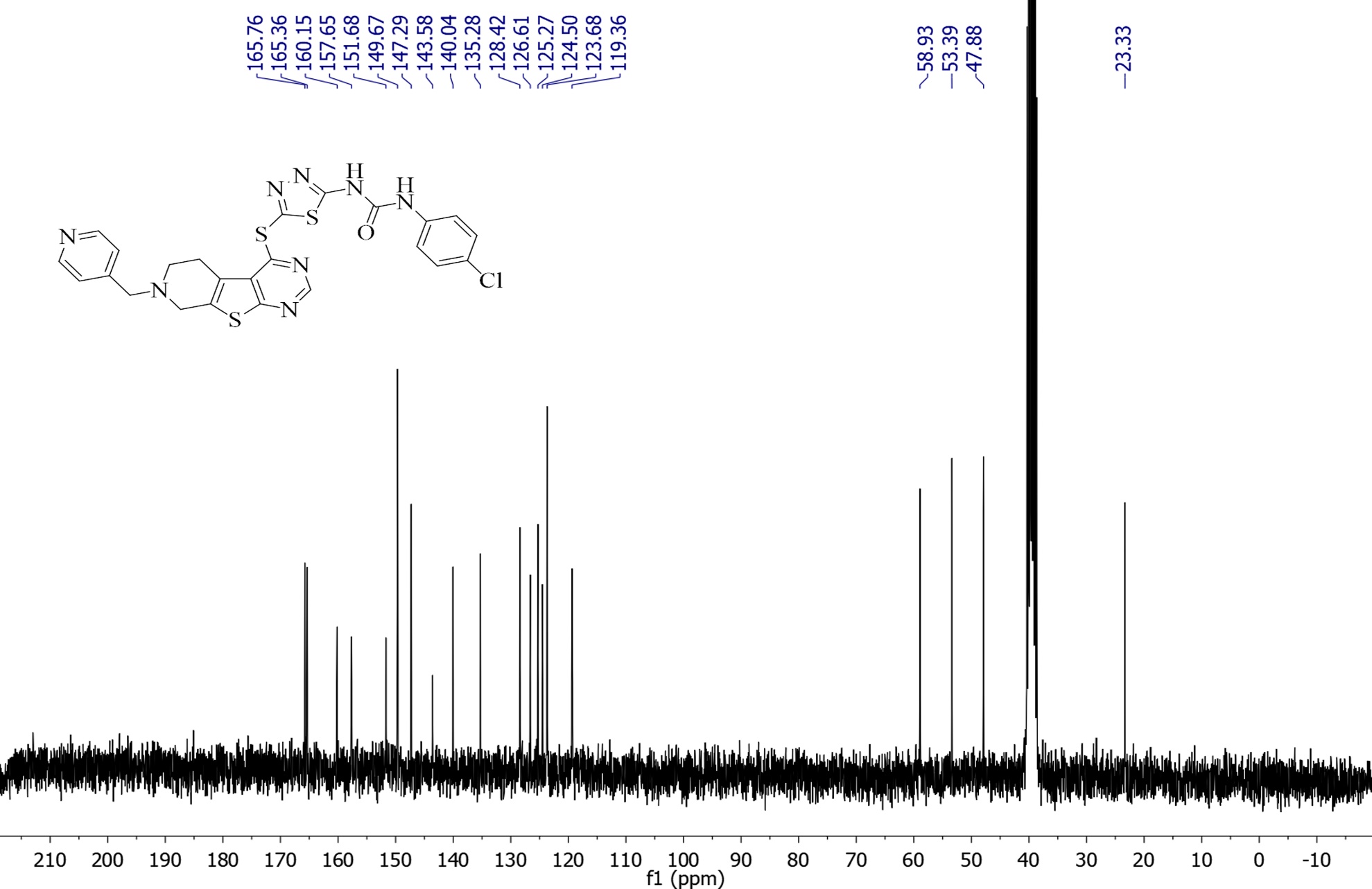
**^13^C NMR spectrum of 1-(4-chlorophenyl)-3-(5-((7-(pyridin-4-ylmethyl)-5,6,7,8-tetrahydropyrido[4',3':4,5]thieno[2,3-d]pyrimidin-4-yl)thio)-1,3,4-thiadiazol-2-yl)urea (**11u**)


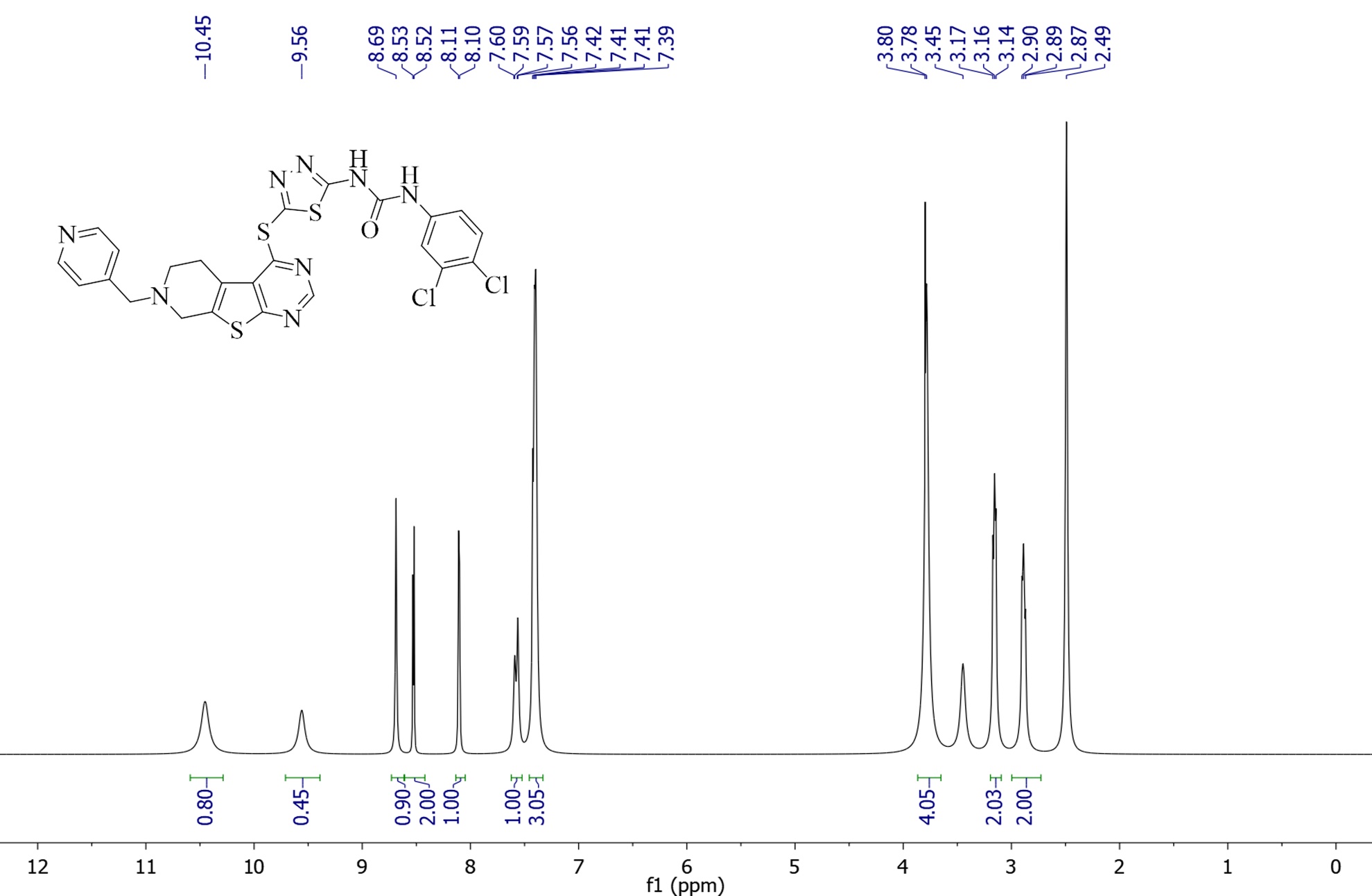
^1^H NMR spectrum of 1-(3,4-dichlorophenyl)-3-(5-((7-(pyridin-4-ylmethyl)-5,6,7,8-tetrahydropyrido[4',3':4,5]thieno[2,3-d]pyrimidin-4-yl)thio)-1,3,4-thiadiazol-2-yl)urea (**11v**)

**
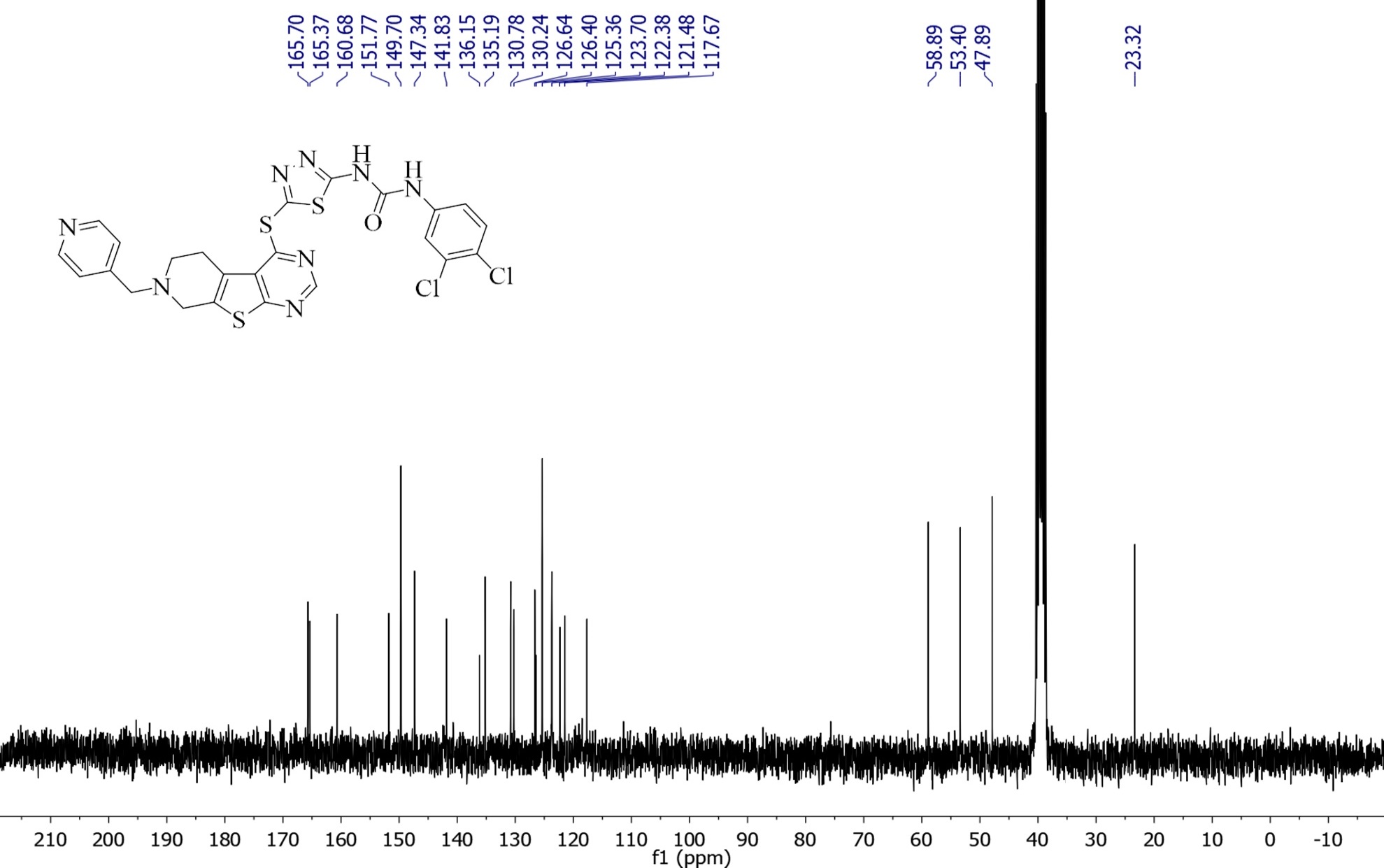
**^13^C NMR spectrum of 1-(3,4-dichlorophenyl)-3-(5-((7-(pyridin-4-ylmethyl)-5,6,7,8-tetrahydropyrido[4',3':4,5]thieno[2,3-d]pyrimidin-4-yl)thio)-1,3,4-thiadiazol-2-yl)urea (**11v**)

**
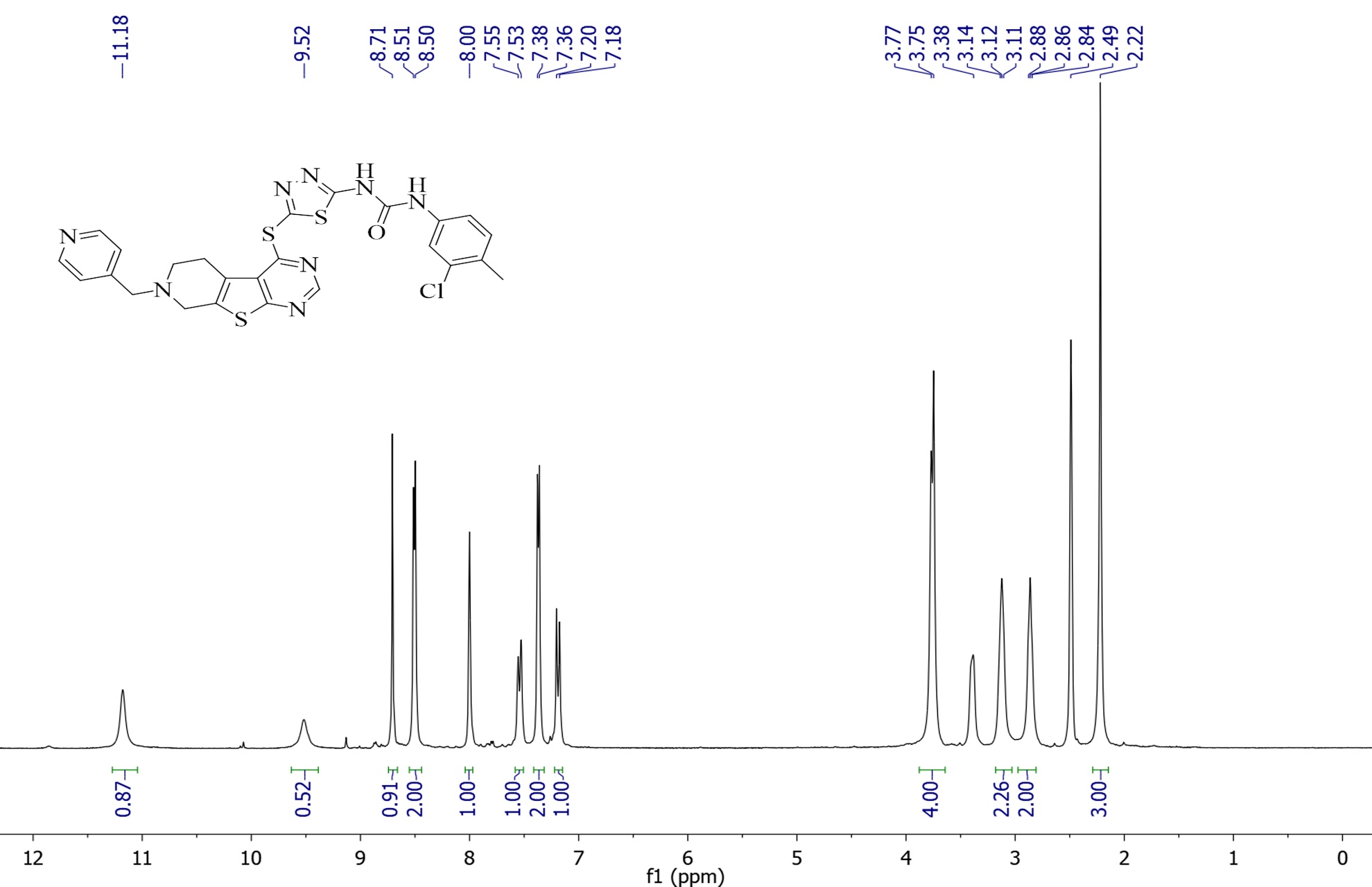
**^1^H NMR spectrum of 1-(3-chloro-4-methylphenyl)-3-(5-((7-(pyridin-4-ylmethyl)-5,6,7,8-tetrahydropyrido[4',3':4,5]thieno[2,3-d]pyrimidin-4-yl)thio)-1,3,4-thiadiazol-2-yl)urea (**11w**)

**
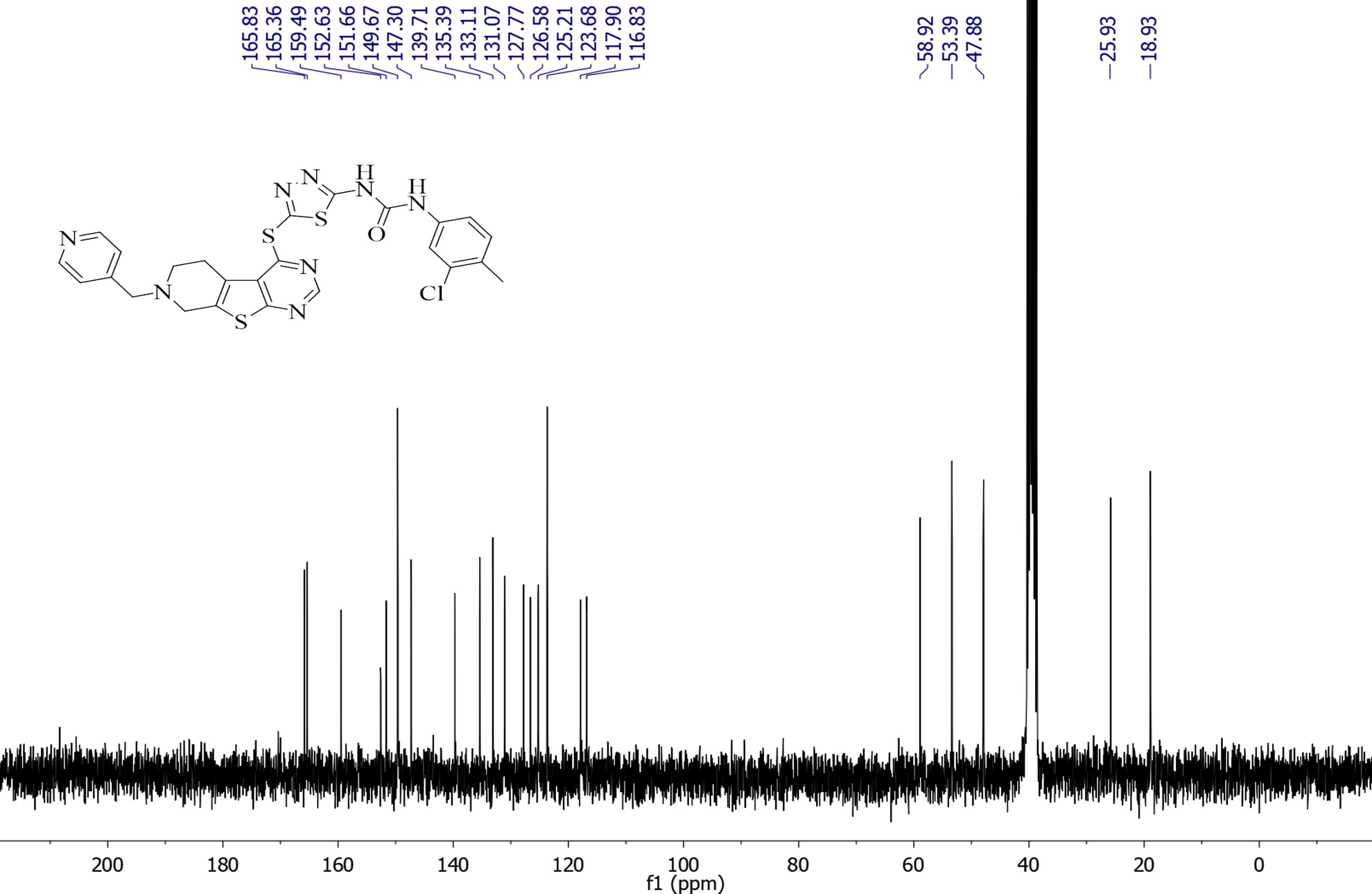
**^13^C NMR spectrum of 1-(3-chloro-4-methylphenyl)-3-(5-((7-(pyridin-4-ylmethyl)-5,6,7,8-tetrahydropyrido[4',3':4,5]thieno[2,3-d]pyrimidin-4-yl)thio)-1,3,4-thiadiazol-2-yl)urea (**11w**)

**
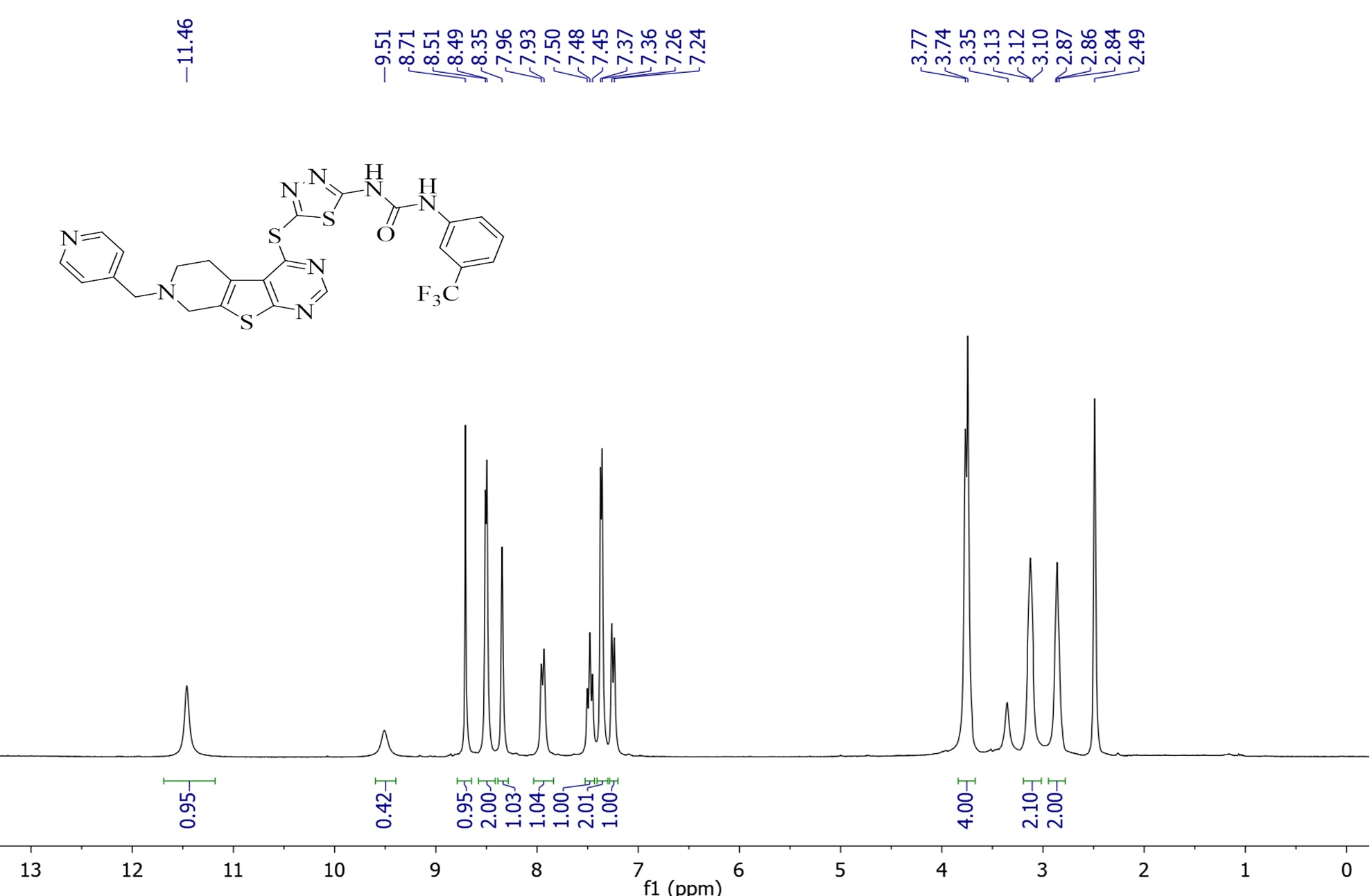
**^1^H NMR spectrum of 1-(5-((7-(pyridin-4-ylmethyl)-5,6,7,8-tetrahydropyrido[4',3':4,5]thieno[2,3-d]pyrimidin-4-yl)thio)-1,3,4-thiadiazol-2-yl)-3-(3-(trifluoromethyl)phenyl)urea (**11x**)

**
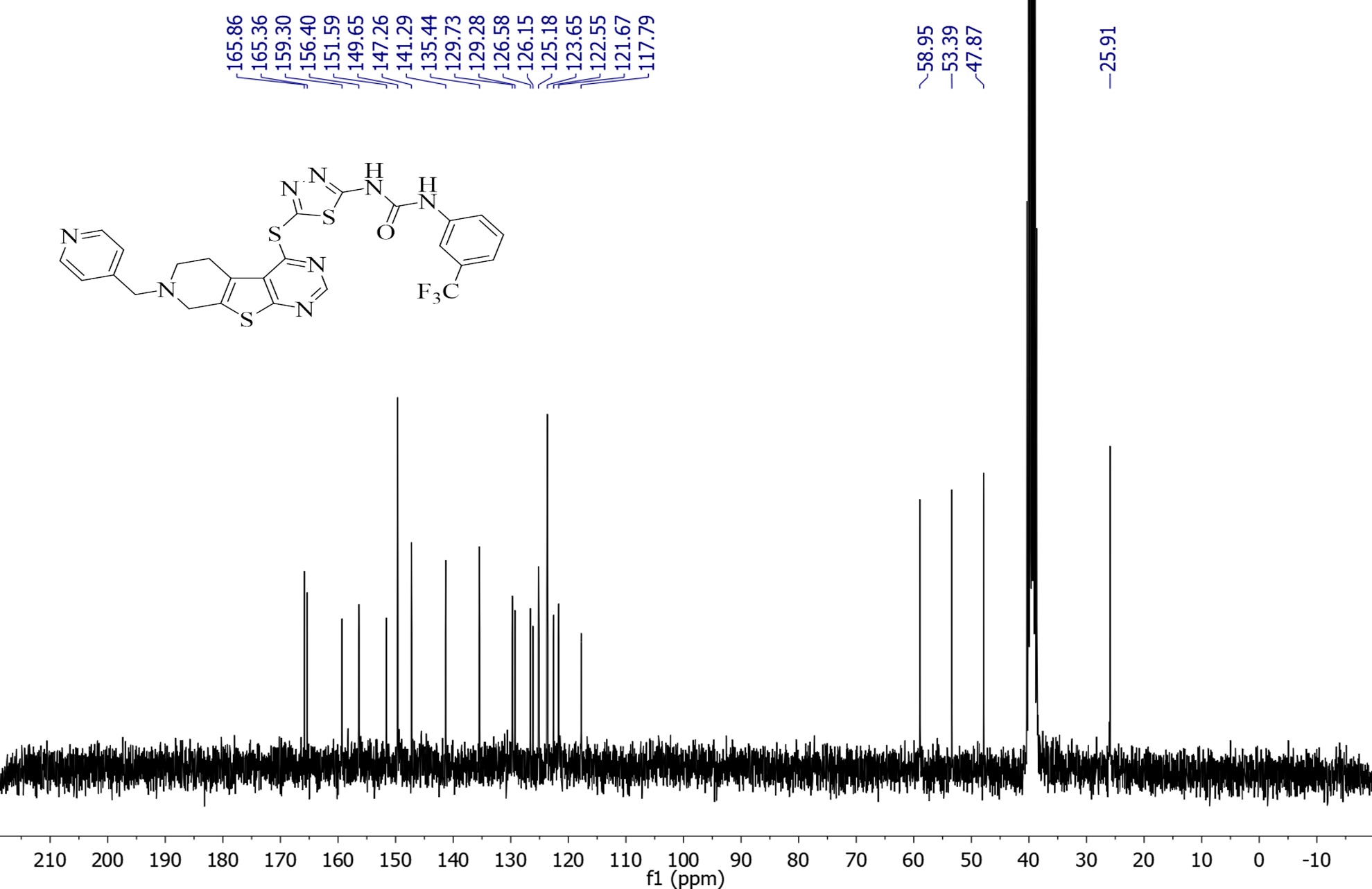
**^13^C NMR spectrum of 1-(5-((7-(pyridin-4-ylmethyl)-5,6,7,8-tetrahydropyrido[4',3':4,5]thieno[2,3-d]pyrimidin-4-yl)thio)-1,3,4-thiadiazol-2-yl)-3-(3-(trifluoromethyl)phenyl)urea (**11x**)

^1^H NMR spectrum of 1-(4-chloro-3-(trifluoromethyl)phenyl)-3-(5-((7-(pyridin-4-ylmethyl)-5,6,7,8-tetrahydropyrido[4',3':4,5]thieno[2,3-d]pyrimidin-4-yl)thio)-1,3,4-thiadiazol-2-yl)urea (**11y**)

**
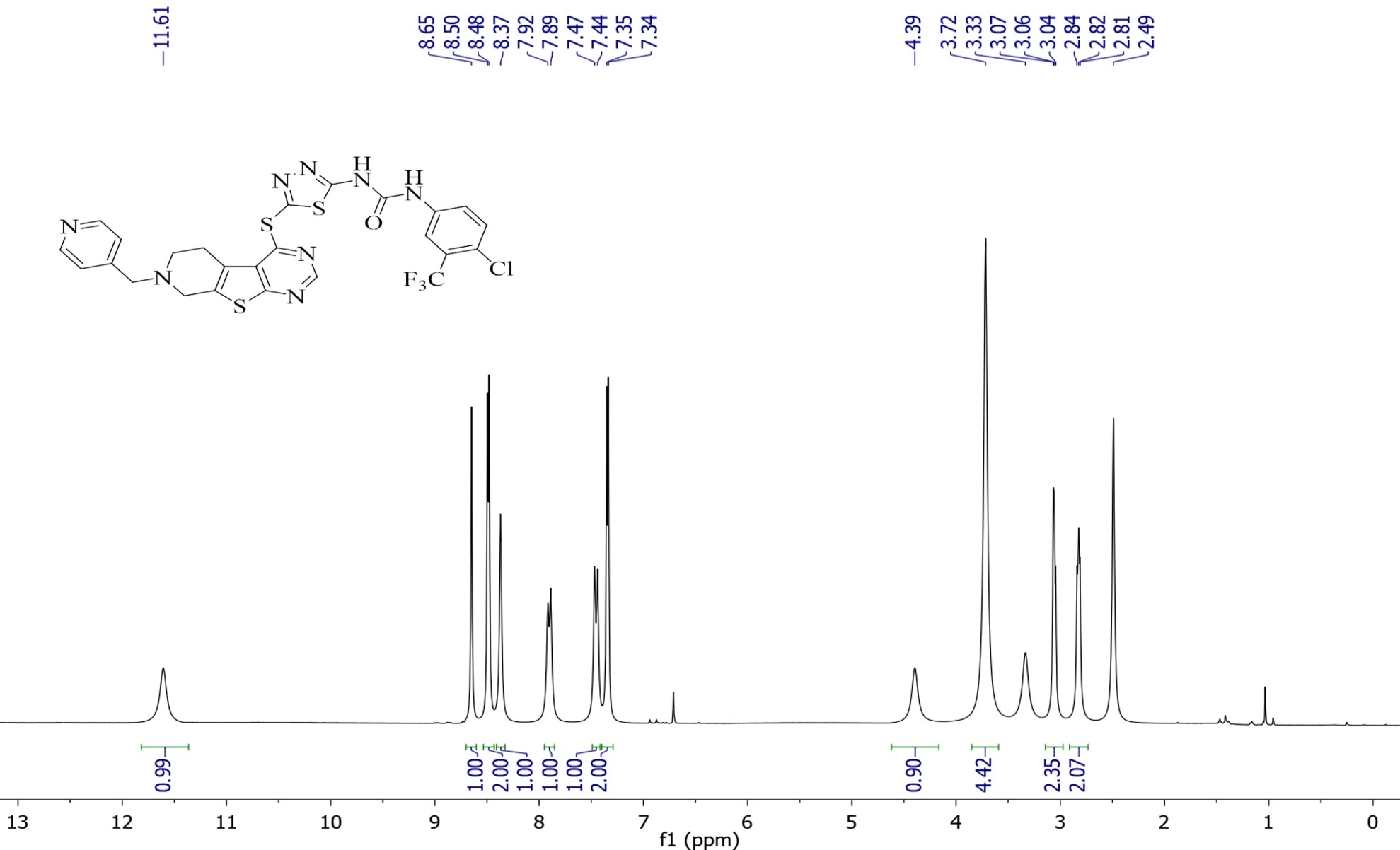
**

**
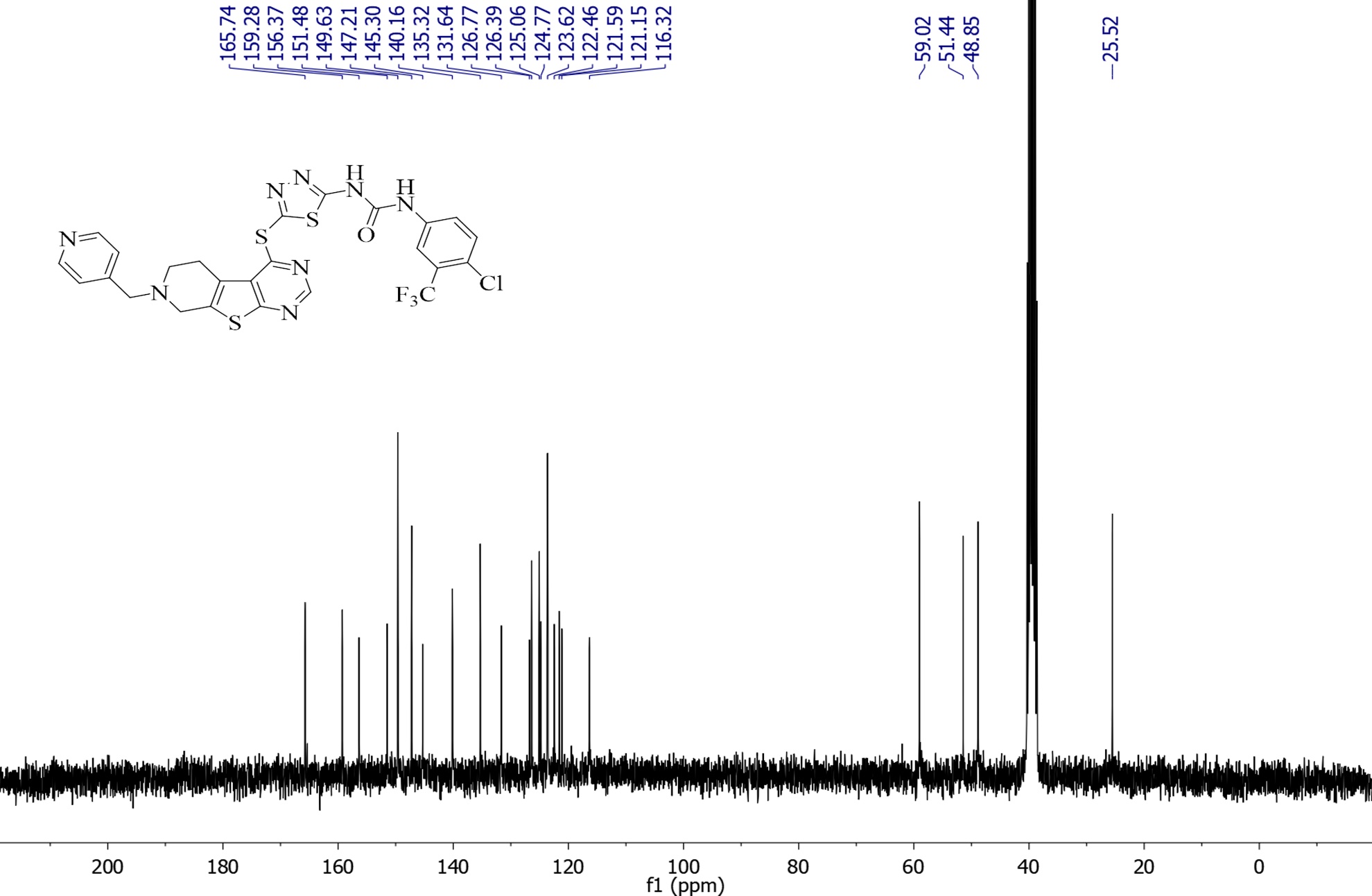
**^13^C NMR spectrum of 1-(4-chloro-3-(trifluoromethyl)phenyl)-3-(5-((7-(pyridin-4-ylmethyl)-5,6,7,8-tetrahydropyrido[4',3':4,5]thieno[2,3-d]pyrimidin-4-yl)thio)-1,3,4-thiadiazol-2-yl)urea (**11y**)
